# Supplementary figures and images for: Is multimodal occupational therapy in addition to usual care cost-effective in people with thumb carpometacarpal osteoarthritis? A cost-utility analysis of a randomised controlled trial
Source: BMJ Open. 2023 Jun 23;13(6):e063103. doi: 10.1136/bmjopen-2022-063103 (PMC10314563; doi:10.1136/bmjopen-2022-063103)

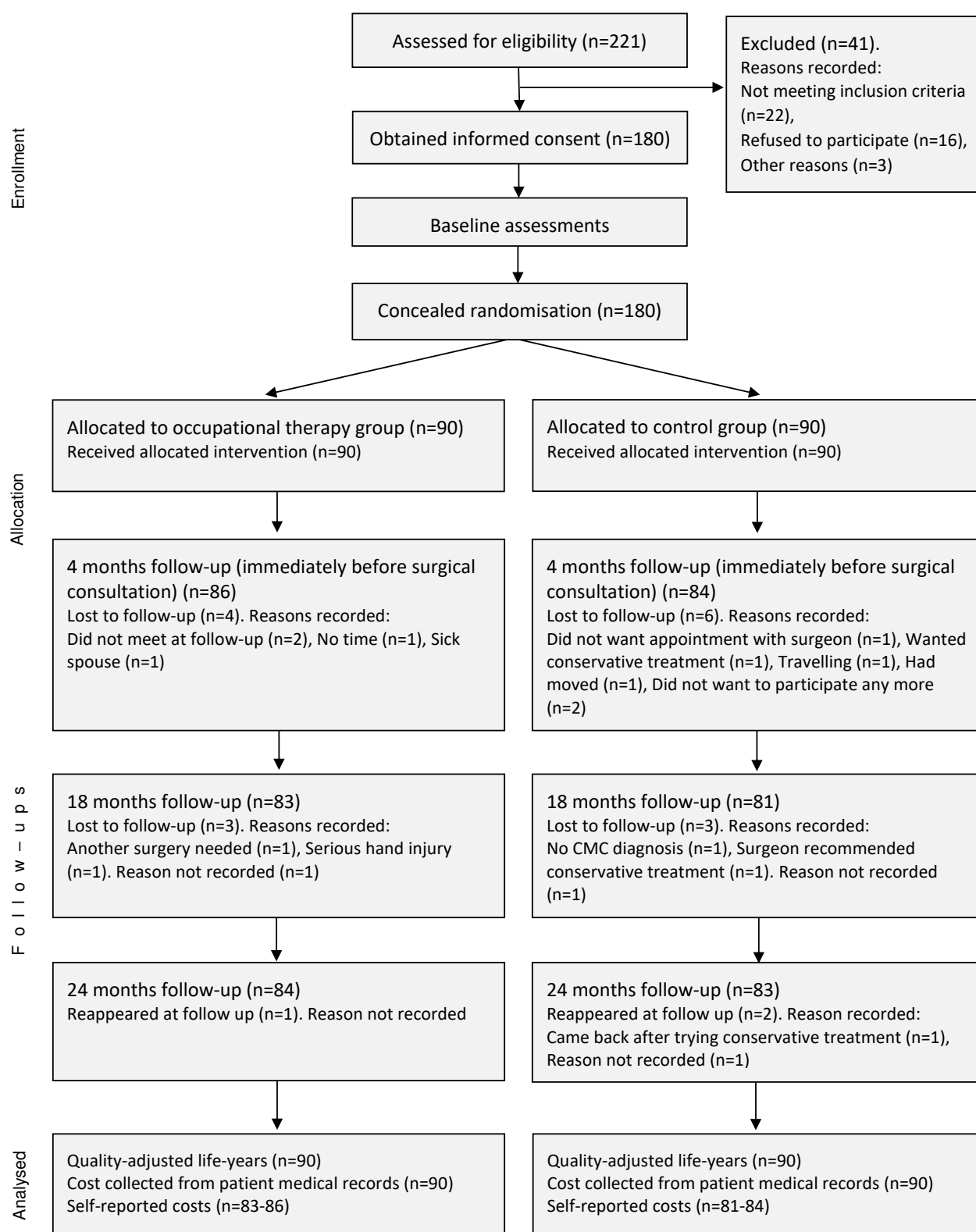

Supplementary figure A

Supplement: Supplementary data [file bmjopen-2022-063103supp001.pdf]

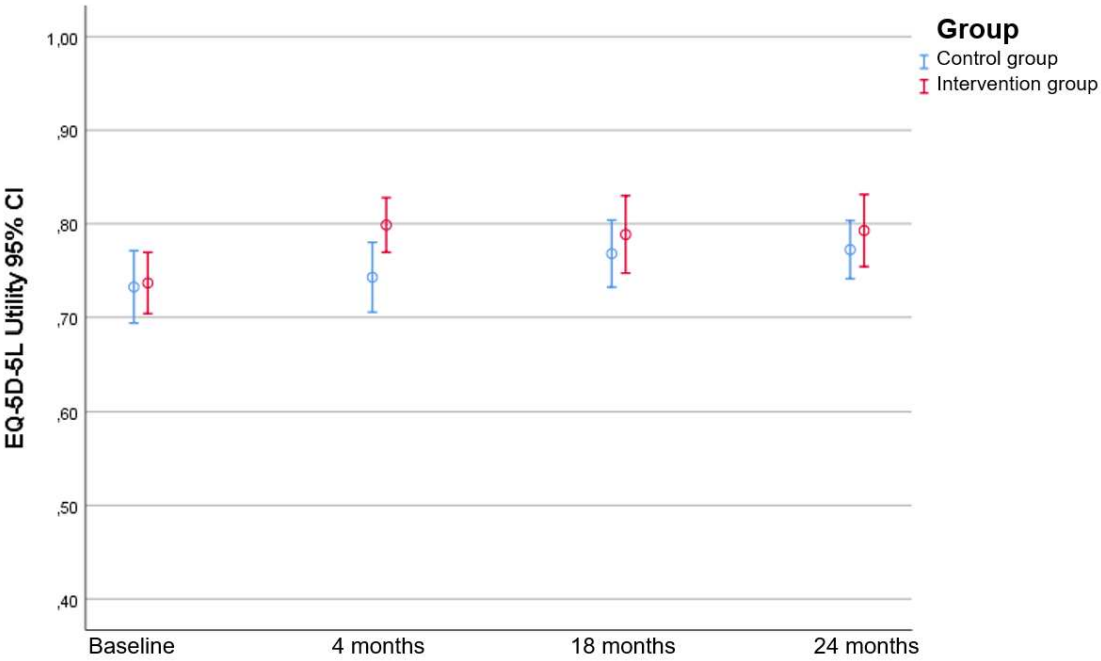

Supplementary figure B

Supplement: Supplementary data [file bmjopen-2022-063103supp002.pdf]
