# Supplementary material for: Is multimodal occupational therapy in addition to usual care cost-effective in people with thumb carpometacarpal osteoarthritis? A cost-utility analysis of a randomised controlled trial
Source: BMJ Open. 2023 Jun 23;13(6):e063103. doi: 10.1136/bmjopen-2022-063103 (PMC10314563; doi:10.1136/bmjopen-2022-063103)
Supplement: Author's manuscript [file bmjopen-2022-063103.draft_revisions.pdf]

BMJ Open is committed to open peer review. As part of this commitment we make the peer review history of every article we publish publicly available.

When an article is published we post the peer reviewers' comments and the authors' responses online. We also post the versions of the paper that were used during peer review. These are the versions that the peer review comments apply to.

The versions of the paper that follow are the versions that were submitted during the peer review process. They are not the versions of record or the final published versions. They should not be cited or distributed as the published version of this manuscript.

BMJ Open is an open access journal and the full, final, typeset and author-corrected version of record of the manuscript is available on our site with no access controls, subscription charges or pay-per-view fees (http://bmjopen.bmj.com).

If you have any questions on BMJ Open's open peer review process please email info.bmjopen@bmj.com

BMJ Open

A cost-utility analysis of multimodal occupational therapy compared to usual care in people with carpometacarpal osteoarthritis

Journal:	*BMJ Open*
Manuscript ID	bmjopen-2022-063103
Article Type:	Original research
Date Submitted by the Author:	11-Apr-2022

Complete List of Authors:	Tveter, Anne Therese; Diakonhjemmet Hospital, Department of Rheumatology and Research<br>Kleven, Linn; Norwegian Institute of Public Health<br>Osteras, Nina; Diakonhjemmet Hosp<br>Nossum, Randi; Trondheim University Hospital<br>Eide, Ruth Else Mehl; Haukeland University Hospital<br>Klokkeide, Åse; Haugesund Rheumatism Hospital AS<br>Matre, Karin Hoegh; Haukeland University Hospital<br>Olsen, Monika; Haugesund Rheumatism Hospital AS<br>Kjeken, Ingvild; Diakonhjemmet Sykehus, National advisory unit on rehabilitation in rheumatology, Department of rheumatology

Keywords:	HEALTH ECONOMICS, Musculoskeletal disorders < ORTHOPAEDIC & TRAUMA SURGERY, Hand & wrist < ORTHOPAEDIC & TRAUMA SURGERY, RHEUMATOLOGY

SCHOLARONE™
Manuscripts

BMJ

I, the Submitting Author has the right to grant and does grant on behalf of all authors of the Work (as defined in the below author licence), an exclusive licence and/or a non-exclusive licence for contributions from authors who are: i) UK Crown employees; ii) where BMJ has agreed a CC-BY licence shall apply, and/or iii) in accordance with the terms applicable for US Federal Government officers or employees acting as part of their official duties; on a worldwide, perpetual, irrevocable, royalty-free basis to BMJ Publishing Group Ltd ("BMJ") its licensees and where the relevant Journal is co-owned by BMJ to the co-owners of the Journal, to publish the Work in this journal and any other BMJ products and to exploit all rights, as set out in our licence.

The Submitting Author accepts and understands that any supply made under these terms is made by BMJ to the Submitting Author unless you are acting as an employee on behalf of your employer or a postgraduate student of an affiliated institution which is paying any applicable article publishing charge ("APC") for Open Access articles. Where the Submitting Author wishes to make the Work available on an Open Access basis (and intends to pay the relevant APC), the terms of reuse of such Open Access shall be governed by a Creative Commons licence – details of these licences and which Creative Commons licence will apply to this Work are set out in our licence referred to above.

Other than as permitted in any relevant BMJ Author's Self Archiving Policies, I confirm this Work has not been accepted for publication elsewhere, is not being considered for publication elsewhere and does not duplicate material already published. I confirm all authors consent to publication of this Work and authorise the granting of this licence.

For peer review only

A cost-utility analysis of multimodal occupational therapy compared to usual care in people with carpometacarpal osteoarthritis

Anne Therese Tveter[1], Linn Kleven[2], Nina Østerås[1], Randi Nossum[3], Ruth Else Mehl Eide[4], Åse Klokkeide[5], Karin Hoegh Matre[4], Monika Olsen[5], Ingvild Kjeken[1]

[1] National Advisory Unit on Rehabilitation in Rheumatology, Department of Rheumatology and Research, Diakonhjemmet Hospital, Oslo, Norway

[2] Norwegian Institute of Public Health, Oslo, Norway

[3] St.Olavs Hospital, Trondheim University Hospital, Trondheim, Norway

[4] Haukeland University Hospital, Bergen, Norway

[5] Haugesund Rheumatism Hospital AS, Haugesund, Norway

Corresponding author:

Anne Therese Tveter

National Advisory Unit on Rehabilitation in Rheumatology, Department of Rheumatology

Diakonhjemmet Hospital

Postboks 23 Vinderen, 0319 Oslo, Norway

AnneTherese.Tveter@diakonsyk.no, https://orcid.org/0000-0003-1701-9835

For peer review only

ABSTRACT

Objective: The aim was to evaluate the cost-effectiveness of a 3-month multimodal occupational therapy intervention compared to usual care in patients with carpometacarpal osteoarthritis (OA).

Methods: A cost-utility analysis was performed alongside a multicentre randomized controlled trial including three rheumatology departments in xxx. A total of 180 patients referred to surgical consultation in specialist healthcare due to carpometacarpal OA were randomized to either multimodal occupational therapy including patient education, hand exercises, assistive devices, and orthoses (n=90), or usual care receiving only information on OA (n=90). The outcome measure was quality-adjusted life-years (QALYs) derived from the generic questionnaire EQ-5D-5L in an intention-to-treat population over a 2-year period. Resource use and health-related quality of life of the patients were prospectively collected at baseline, 4, 18, and 24 months. Costs were estimated by taking a healthcare perspective. The results were expressed as incremental cost-effectiveness ratios (ICERs), and bootstrapping was done to account for uncertainty in the analysis.

Results: During the 2-year follow-up period, patients receiving multimodal occupational therapy gained 0.06 more QALYs than patients receiving usual care. The mean (SD) direct costs were € 3227 (3546) in the intervention group and € 4378 (5487) in the usual care group, mean difference €-1151 (95% CI -2564, 262). The intervention was the dominant treatment with a probability of 94.5% of being cost-effective given the willingness-to-pay threshold of € 27 500.

Conclusions: The within-trial analysis demonstrated that the multimodal occupational therapy was cost-effective compared to usual care in patients with carpometacarpal osteoarthritis.

Trial registration number: ClinicalTrials.gov (NCT01794754)

Keywords: hand osteoarthritis, occupational therapy, cost-effectiveness, cost-utility, quality-adjusted life-years (QALYs)

For peer review only

Strength and limitations of the study

- This is the first study assessing the cost-effectiveness of recommended multimodal treatment compared to usual care in patients with carpometacarpal osteoarthritis
- The results shows that multimodal therapy is cost-effective compared to usual care over a 2-year period
- Self-reported costs combined with several months between assessments may have biased the total costs of the study

INTRODUCTION

Hand osteoarthritis (OA) is the most common joint disease,[1] with the subcategory carpometacarpal osteoarthritis (CMC1 OA) affecting the base of the thumb.[2] Approximately 22% of people aged ≥50 years have symptomatic CMC1 OA[3]. Compared to OA in the other finger joints, CMC1 OA is associated with more pain and functional limitations, negatively affecting work ability and quality of life.[2,4,5]

There is a common misconception that hand OA is a normal part of aging, and that there is 'nothing to be done'.[6] Although there is no cure for OA, the European League Against Rheumatism (EULAR) recommends that all people with hand OA should be offered a multimodal intervention consisting of information, hand exercises, and assistive devices as first-line treatment.[7] In addition, orthoses should be provided for patients with CMC1 OA.[7] Pharmacological therapy is recommended as a symptom relieving supplement, whereas surgery should only be considered when other treatments have failed to provide sufficient pain relief.[7] The multimodal intervention is shown to have a significant short-term effect on pain and hand function compared to usual care.[8] However, there is an evidence-to-practice gap regarding this first-line treatment, with only 20% reporting to have received this treatment before being referred to surgical consultation.[9] This intervention also presents a tendency towards reduction and delay in surgery rate over a 2-year period.[10] Although not significant, this tendency may still be of clinical importance if shown to be cost-effective.

For peer review only

Oppong et al,[11] performed a trial-based economic evaluation on joint protection only, hand exercises only and a combination of joint protection and hand exercises compared to leaflet and advice over a 12-month period in patients with hand OA. They found hand exercises to be the most cost-effective alternative.[11] To the best of our knowledge, no studies have assessed the cost-effectiveness of a multimodal intervention following the EULAR recommendations for the treatment of hand OA or CMC1 OA.

The aim of this study was to perform a cost-utility analysis of a 3-month multimodal occupational therapy intervention compared to usual care in patients with CMC1 OA. The outcome measure was quality adjusted life-years (QALYs), and the costs were estimated both as direct and indirect costs and societal costs over a 2-year period taking a healthcare perspective.

METHODS

Study design

A cost-utility analysis (CUA) was performed alongside a multicentre RCT including rheumatology departments in three xxx hospitals (xxx in xxx, xxx in xxx, and xxx) and is described in the protocol article.[10,12] The trial was approved by the xxx Regional Ethical Committee (2012/2265/xxx).

Patient and Public Involvement

A patient research partner from the Patient Research Panel at xxx was involved throughout the project. She gave input to the project description and the conduct of the study and participated in seminars where she gave feedback on the interpretation and dissemination of the results.

Participants and randomization

Patients referred by their general practitioner for surgical consultation due to CMC1 OA at the three rheumatology departments between 2013 and 2015 were considered eligible for the study if they could speak Norwegian and had no cognitive deficits. Eligible patients received information about the study, a written consent form and a questionnaire by post. Patients who agreed to participate were

For peer review only

scheduled for an appointment with an occupational therapist (OT) at the hospital within a short period of time after referral. After a baseline assessment, the patients were randomized to either a 3-month multimodal occupational therapy intervention or usual care. In xxx, the waiting period between referral and the actual surgical consultation was at that time between 4 and 6 months, and the intervention was conducted during this waiting period. Patients were assessed again immediately before surgical consultation (approximately 4 months after baseline assessment), and 18 and 24 months after the baseline assessment (Supplementary figure A). The sample size of 180 participants was estimated based on an expected surgery rate of 70% during the 2-year follow-up, which was the primary endpoint of the RCT.[10,12]

Intervention

During the waiting period between referral and surgical consultation, the intervention group received a multimodal occupational therapy intervention in line with recommended first-line treatment.[7] At the baseline assessment, the patients received written and oral information on hand OA, ergonomic principals, and the use of assistive devices. They were instructed in a hand exercise program to be performed at home three times per week for 12 weeks. They all received five common assistive devices (a bread knife and vegetable knife with built-up handles, an enlarged grip for opening bottles, a key for opening jars [the Jar Key], and a self-opening pair of scissors) for use when needed, and daytime and nighttime orthoses that they were encouraged to use as much as possible.[12] The patients in the intervention group consulted the OT twice during this intervention period: at the baseline assessment and again after 2 weeks to adjust the orthoses and exercise program, implying that the intervention was based mostly on self-management. Patients referred for bilateral hand OA received treatment for both hands. At the follow-up assessments (4, 18, and 24 months), the patients in the intervention group could receive new orthoses, or the OTs could adjust the ones they already had.

The control group received usual care, which generally means no treatment. However, for the purpose of this study, the patients in the control group also received written and oral information on hand OA at the baseline assessment.

For peer review only

Both groups had a surgical consultation with a medical specialist approximately 4 months after the baseline assessment and were assessed by an OT at all follow-ups. If necessary, patients in both groups could seek additional treatment, such as a consultation with general practitioner or other healthcare personnel.

Data collection

Health outcome

The health-related quality of life (HRQoL) was measured at baseline, 4, 18, and 24 months using the generic instrument EQ-5D-5L (EuroQol 5-dimension, www.euroqol.org).[13] The EQ-5D-5L is a preference weighted measure of HRQoL based on five dimensions: mobility, self-care, daily activities, pain/discomfort, and anxiety/depression. For each dimensions the patient assesses five possible levels of problems, from no problems to severe.[13] Quality-adjusted life-years (QALYs) were derived from the EQ-5D-5L utility score, using the preference score from a UK population.[14] Since there is no xxx tariff, the recommendation in xxx is to use the British tariff.[15] QALYs are calculated by adjusting time (years of life) with an index that express the HRQoL (utilities) on a scale anchored at 0 (health state equivalent to death) and 1 QALY (full health).

Resource use and costs

Healthcare utilization was collected from different sources at baseline, 4, 18 and 24 months. The OT reported the number of consultations related to the intervention at each assessment, whereas surgical procedures and post-operative treatment were collected from patients' medical record. In addition, the use of primary and specialist healthcare was self-reported by the patient. We have included the costs related to the OT assessment at baseline, 4, and 18 months for both groups (OT assessment at 24 months were not included as the usual care group then received the intervention). Although the OT assessment was done for study purposes, it was included in the cost estimates because we cannot rule out a possible positive effect of an appointment with an OT in specialist healthcare for the control group. We have not included medication costs or travelling expenses.

Data on sickness absence related to surgery were collected from patient medical records. The number of hours of informal help at home due to OA (from spouse, family, or friends) and the productivity

For peer review only

loss (due to any reason of sickness absence and disability benefits, excluding sickness absence related to surgery) were self-reported. If not otherwise indicated, the productivity loss at any given assessment point was forwarded to the next assessment, i.e. reported sickness absence of 50% at 4 months was calculated as 50% sickness absence from 4 to 18 months.

The costs of specialist healthcare visits and surgery were calculated using DRG weights (diagnosis-related group) from 2019. DRG is a coding system used for administration of both clinical and financial activity in specialist healthcare. The DRG costs were estimated by multiplying the cost weighting for the specific DRG group of the patient with the unit cost of 2019.[16] Sickness absence and disability benefits were valued in the same way for all patients. Costs related to absence from work were estimated from official statistics on the average wage by sex and age group in 2019. The average daily productivity loss was valued as € 336 including societal costs of 40% and was adjusted for the percentage of sickness absence/disability benefits.[17] The number of hours of informal help at home was valued as a minimum wage of € 18 per hour.[18] All costs are estimated in Euros, using the exchange rate 0.10 (€ 1 = NOK 10) from 2019.

Direct costs included costs related to specialist healthcare (medical specialist, occupational therapist, physical therapist, nurse, radiographs, surgery, assistive devices) and primary healthcare (general practitioner, physical therapist, manual therapist, acupuncturist, blood samples). Indirect costs included productivity loss due to sickness absence (due to surgery or for other reasons) and costs related to help with chores at home.

Statistics

Differences in QALYs between the two groups were estimated using the trapezoidal method (area under the curve).[19] To illustrate the statistical uncertainty surrounding the incremental cost-effectiveness ratio (ICER), a probabilistic sensitivity analysis with 1 000 replications was performed using the bootstrap resampling method. The bootstrapped costs and effects can be illustrated on a cost-effectiveness plane (CE plane). The CE plane (Figure 1) are divided into four quadrants; where a new treatment that is more effective and less costly is plotted in the southeast quadrant, while one more costly and less effective is plotted in the northwest quadrant. Figure 2 shows the cost-effectiveness acceptability curve (CEAC), which

For peer review only

presents the probability that the intervention is cost-effective compared to usual care for hand OA. The willingness-to-pay threshold was set to € 27 500 due to the low severity of the condition.[20]

Between-group differences in mean costs and mean effects were analyzed using Independent sample t-test. Significance level was set to 0.05. Statistical analyses were performed using Excel, SPSS version 26 and Stata version 16.

Missing data

A total of 18 patients (7 in the intervention group and 11 in the control group) had missing HRQoL utility score at one or more timepoints. For these missing values we used 'last observation carried forward'. Sensitivity analyses showed that the average HRQoL utility scores remained the same for both groups without imputation of these missing values (Table 3). Missing self-reported costs were not imputed (n=10). The costs related to surgery were collected from patient medical records and were not encumbered by missing data.

RESULTS

Baseline characteristics

A total of 180 patients were randomized to either multimodal occupational therapy (n=90) or usual care (n=90). The mean age across both groups was 63 years (SD 7.6) and 79% of the patients were women. We found no between-group differences in any of the baseline characteristics including utility score, medication use, or percentage of patients working full- or part-time (Table 1).

For peer review only

Table 1 Demographic variables for patients referred to surgical consultation due to carpometacarpal osteoarthritis (n = 180)

	**Intervention group (n=90)**	**Control group (n=90)**
Age, years, mean (SD)	62.8 (7.5)	63.3 (7.8)
Female sex, *n* (%)	73 (81)	69 (77)
Referred hand, *n* (%)		
Right	31 (34)	17 (19)
Left	24 (27)	29 (32)
Both	35 (39)	44 (49)
Education level, *n* (%)		
Primary school	14 (16)	20 (22)
High school	44 (49)	39 (43)
University/college ≤ 4 y	17 (19)	19 (21)
University/college > 4 y	15 (16)	12 (13)
Living alone, *n* (%)	17 (19)	18 (20)
Working full/part-time, *n* (%)	48 (53)	43 (48)
Years with symptoms, median (IQR)‡	5 (2–10)	5 (2–10)
Presence of subluxation of the CMC joint	58 (64)	55 (61)
Comorbidity, *n* (%)	61 (68)	56 (62)
Analgesics use (anti-inflammatory or pain relief medication), *n* (%)	57 (63)	57 (63)
Pain at rest (NRS 0–10, 0 = no pain), median (IQR)	3 (1–4)	3 (2–5)
EQ-5D-5L utility score (1=best health), mean (SD)	0.74 (0.16)	0.73 (0.18)

SD= standard deviation; IQR= interquartile range (25th and 75th percentile); CMC= carpometacarpal joint; NRS= numeric rating scale; Pain at rest was reported for referred hand (the mean of both hands if bilateral referral)

Cost-utility analysis

The total between-group difference in QALYs was 0.06 (95% CI -0.02, 0.15) after 24 months, in favor of the intervention group. The mean healthcare costs and productivity loss for both treatment groups are presented in Table 2.

For peer review only

Table 2 Mean direct and indirect healthcare costs per patient for the intervention and control group (n=180)

		Unit	Unit price, €	Intervention group<br>Mean (SD) per patient, €	Control group<br>Mean (SD) per patient, €	Mean difference (95% CI), €
Direct costs	*Specialist healthcare*					
	Medical specialist	Per visit	205[a]	309 (268)	384 (365)	-75 (-169, 19)
	Occupational therapist including adaptation of orthoses	Per visit	246[a]	536 (318)	158 (367)	377 (275, 478)
	Occupational / Physical therapist	Per visit	205[a]	428 (350)	837 (866)	-355 (-549, -160)
	Nurse	Per visit	205[a]	16 (98)	48 (189)	-32 (-76, 13)
	Inpatient surgery	Per surgery	8 435[a]	187 (1 250)	562 (2 465)	-375 (-951, 201)
	Day-bed surgery	Per surgery	2 251[a]	500 (941)	675 (1 190)	-175 (-491, 141)
	Radiographs	Per visit	149[e]	149 (0)	149 (0)	-
	Assistive devices	Per person	67[e]	67 (0)	-	-
	Primary healthcare					
	General practitioner	Per visit	32[b]	32 (94)	46 (104)	-14 (-43, 15)
	Physical therapist	Per visit	31[c]	64 (288)	54 (160)	10 (-59, 79)
	Manual therapist	Per visit	52[c]	-	10 (90)	-
	Acupuncturist	Per visit	47[e]	11 (64)	23 (132)	-11 (-42, 19)
	Blood samples	Per test	7[b]	1 (6)	2 (6)	-1 (-2, 1)
Indirect costs	Sickness absence due to surgery	Days	336[d]	1 212 (3 815)	1 199 (3 817)	186 (-1 104, 1 141)
	Costs related to help with chores at home	Hours	18[d]	652 (1 722)	922 (3 089)	-270 (-1 006, 465)
	Sickness absence and disability benefits (not sickness absence due to surgery)	Days	336[d]	38 299 (63 029)	45 433 (66 320)	-7 133 (-26 167, 1 190)
				Total per group, €	***Total per group, €***	
Direct costs				290 391	394 004	
Indirect costs				3 905 107	4 673 370	
Total costs (direct + indirect costs)				4 195 498	5 067 374	

[a] DRG (diagnosis-related group system); [b] List of reimbursement codes; [c] Rates for physiotherapists; [d] Official statistics of average wage; [e] Based on real costs. All costs are given in Euro (€1=NOK10) and adjusted to 2019.

The mean cost of multimodal occupational therapy intervention (baseline information on hand OA, instruction in hand exercises, assistive devices, and customization of orthoses, and adjustment of hand exercises and orthoses after 14 days) was approximately € 500 per patient. Surgery accounted for the largest between-group difference with 22 surgical procedures in the intervention group and 33 surgical procedures in the control group, resulting in a €550 difference in surgical cost per patient between the two groups. The use of physiotherapy/occupational therapy also constituted a high cost. However, almost one-third of these costs were related to the predetermined assessment points (4, 18 and 24 months) that were part of the study but not the actual OT intervention (€375 per patient in the control group and €307 per patient in the intervention group).

Productivity loss due to sickness absence or disability benefits was the largest contributor to the total costs in both groups. However, at baseline, only 7% of the sickness absence in both groups was reported to be due to hand OA, and the percentages at 4, 18, and 24 months were 6%, 2% and 0.5%, respectively. Similar numbers of patients in the two groups were retired at baseline (34% in the control group versus 32% in the intervention group), 4 months (36% versus 34%), and 24 months (42% versus 43%). However, more patients in the intervention group (43%) retired between 4 and 18 months than in the control group (36%), indicating that fewer patients contributed to productivity loss in the intervention group during this period.

Table 3 shows the mean incremental costs and incremental effects associated with direct and indirect healthcare costs. In the case of a negative ICER, the evaluated treatment is either dominant (more effective and less costly) or dominated (less effective and more costly). The intervention in this study was the dominant treatment (Figure 1) with a 94.5% probability of being cost-effective given the willingness-to-pay threshold (Figure 2), indicating that the intervention was both less costly and more effective compared to usual care.

For peer review only - http://bmjopen.bmj.com/site/about/guidelines.xhtml

Table 3 Cost-utility analysis of mean costs, mean effects, incremental differences in costs and effects (95% CI), and incremental cost-effectiveness ratio (ICER) for direct and total direct and indirect costs (n=180)

	Intervention group (n=90)		Control group (n=90)				
	Mean cost (SD)	Mean effect* (SD)	Mean cost (SD)	Mean effect* (SD)	Incremental cost (95% CI)	Incremental effect (95% CI)	ICER
Direct costs	3 227 (3 546)	1.58 (0.28)	4 378 (5 487)	1.51 (0.28)	-1 151 (-2 564, 262)	0.06 (-0.02, 0.15)	Dominant
Total costs (direct and indirect costs)	46 617 (65 499)	1.58 (0.28)	56 304 (68 872)	1.51 (0.28)	-9 688 (-9 949, 29 324)	0.06 (-0.02, 0.15)	Dominant

Direct costs, **€**: self-reported use of primary and specialist healthcare, including surgery;

Indirect costs, **€**: productivity loss from sickness absence due to surgery, costs related to informal help at home, productivity loss from sickness absence and disability benefits excluding sickness absence due to surgery

Total cost, **€**: direct costs + indirect costs

* Mean effect without imputations; Intervention group 1.58 (0.28), Control group 1.51 (0.28)

For peer review only - http://bmjopen.bmj.com/site/about/guidelines.xhtml

DISCUSSION

The aim of this study was to evaluate the cost-effectiveness of a 3-month multimodal occupational therapy intervention in patients with CMC1 OA in a 2-year period. Our results demonstrate that the intervention, consisting of patient education, hand exercises, orthoses, and assistive devices, has the highest probability of being cost-effective, given the willingness-to-pay threshold (€ 27 500/QALY). This study is one of few studies to evaluate the cost-effectiveness of conservative treatment in hand OA, and the only study to evaluate the cost-effectiveness of the 2018 EULAR guidelines on first-line treatment for CMC1 OA. Our findings add to the evidence base by demonstrating that multimodal occupational therapy is a cost-effective alternative for the management of CMC1 OA.

The results of this study are in line with a previous study showing that hand exercises were more cost-effective than leaflet and advice in patients with hand OA.[11] In contrast, the study by Oppong et al. failed to show that the combination of hand exercises and use of assistive devices (joint protection classes[21]) were more cost-effective than hand exercises alone.[11] In our study, we did not separately evaluate the different parts of the recommended first-line treatment. It is possible that we would have found the same results as Oppong et al.,[11] given that hand exercises only entail costs related to the time used by health personnel to educate the patient, whereas both orthoses and assistive devices require an additional cost. On the other hand, the combination of the recommended first-line treatment was found to have a significant short-term effect on reducing pain and improving hand function in these patients,[8] and should be offered to all patients with CMC1 OA as an effective and cost-effective alternative. Due to the self-managing nature of the intervention, the mean cost related to the multimodal occupational therapy intervention was low, approximately €500 per patient. However, the current study was conducted in specialist healthcare, and there are other options for delivering this intervention that may be even more cost-effective. The treatment of patients with OA should mainly be conducted in primary healthcare, possibly reducing the costs to some extent. In addition, more cost-effective options could be to provide the intervention by utilizing a smart phone application as shown in a Swedish study on digital first-line treatment for hip and knee OA.[22] Thus, different ways of delivering this first-line hand OA treatment should be further explored.

For peer review only - http://bmjopen.bmj.com/site/about/guidelines.xhtml

For peer review only

Surgery constituted a large cost in this within-trial. Even if we found a tendency towards reduction in surgical procedures in the intervention group compared to the control group, this result was not statistically significant.[10] Still, the between-group difference in mean surgical costs (€ 550) was higher than the mean cost of the multimodal occupational therapy intervention alone (€ 500). Thus, although the difference in surgical rates between the two groups were found to be non-significant,[10] the difference may still be regarded as important in a healthcare perspective as surgical procedures may be more prone to more adverse events compared to multimodal occupational therapy.[10]

The patients reported to have OA complaints for approximately 5 years, with more than 60% showing radiographic signs of subluxation of the carpometacarpal joint. Only 21% of the patients had visited a physiotherapist or an OT before referral to surgical consultation.[10] Thus, referral to a multimodal occupational therapy intervention at an earlier stage in the disease course may be a possible step towards reducing the costs related to healthcare use.

The major strengths of the current study are the large sample size and to be the first study assessing the cost-effectiveness of recommended multimodal treatment in patients with CMC1 OA. The study also has some limitations. Almost 80% of the sample were women, which represents a higher proportion of women than the usual gender distribution in hand OA. The results may therefore not be representative for men with hand OA. Most of the costs reported in this study were self-reported costs and, combined with several months between assessments, the results may be influenced by recall bias. This has most likely contributed to lower overall costs, as patients may not remember to report all consultations with healthcare personnel, especially in the time period between 4 and 18 months. On the other hand, the costs related to surgery were collected from patient medical records and will not be encumbered by the same bias. A total of 10 patients had missing data throughout the study. There may have been systematic reasons for these patients being missing, which could have affected the outcome. However, there were more missing data in the control group than the intervention group, indicating that the between-group difference could have been larger if all patients had reported direct and indirect costs. The results may have been biased the method of imputation of missing HRQoL utility values. However, due to low number of missing values and sensitivity analyses showing no difference in mean score whether using 'Last

For peer review only - http://bmjopen.bmj.com/site/about/guidelines.xhtml

observation carried forward' or not, we did not go any further with more advanced methods like multiple imputation. We were unable to include medication costs and travel expenses in the analyses. This is not in line with the most recent recommendations for cost-effectiveness analyses[23] and may have biased the results by possibly underestimating the total costs. However, the equal distribution of medication use across the two groups imply that this do not constitute a large bias in the comparison of results. Although the follow-up sessions were similar for both groups, the results may have been influenced by the fact that patients in the intervention group could receive adjustment of orthoses or new orthoses at these sessions. This may have influenced the results. Future studies should evaluate the cost-effectiveness of an even longer follow-up time as there is a lack of knowledge on surgical rate of these patients in the long term.

CONCLUSION

The within-trial analysis demonstrated that multimodal occupational therapy was cost-effective compared to usual care in patients with CMC1 OA, when taking a healthcare perspective. The intervention was the dominant treatment with a 94.5%, probability of being cost-effective, given the willingness-to-pay threshold of € 27 500/QALY. The low cost related to the intervention supports the use of this first-line treatment in all patients with CMC1 OA.

Acknowledgements

We would like to acknowledge patient research partner Øyvor Andreassen for her contribution throughout the project. We would also like to thank professor Tore K. Kvien for reading and commenting on the manuscript. The project has been acknowledged at the EULAR Congress with the HPR Abstract Award in 2019 for the primary outcome results[24] and the HPR Abstract Award in 2020 for the results presented in this article.[25]

Author contribution

For peer review only - http://bmjopen.bmj.com/site/about/guidelines.xhtml

For peer review only

Ingvild Kjeken was responsible for obtaining funding. Randi Nossum, Ruth Else Mehl Eide, Åse Klokkeide, Karin Hoegh Matre, and Monika Olsen contributed to data acquisition. Anne Therese Tveter, Linn Kleven, Nina Østerås, and Ingvild Kjeken were responsible for data analysis and interpretation. Further, all authors have made substantial contributions to the study conception and design, drafting of the article, critical revision of the article for important intellectual content and final approval of the submitted version.

Funding statement

This work was supported by the South-Eastern Norway Regional Health Authority (2015109). The funding authorities had otherwise no involvement in the study. This research received no specific grant from any funding agency in the public, commercial or not-for-profit sector.

Conflict of interest

None of the authors have any financial or personal conflicts of interest.

Data sharing statement

Data may be made available upon reasonable request to the project manager (Ingvild Kjeken, Ingvild. kjeken@diakonsyk.no)

Ethics Statement

The trial was approved by the Norwegian Regional Ethical Committee (2012/2265/REK sør-øst C). All participants gave a written informed consent before taking part in the study.

For peer review only

References

1. Marshall M, Watt FE, Vincent TL, Dziedzic K. Hand osteoarthritis: clinical phenotypes, molecular mechanisms and disease management. *Nature Reviews Rheumatology.* 2018;14(11):641-656:10.1038/s41584-018-0095-4
2. Bijsterbosch J, Visser W, Kroon HM, Stamm T, Meulenbelt I, Huizinga TW, et al. Thumb base involvement in symptomatic hand osteoarthritis is associated with more pain and functional disability. *Annals of the rheumatic diseases.* 2010;69(3):585-587:10.1136/ard.2009.104562
3. Marshall M, Peat G, Nicholls E, van der Windt D, Myers H, Dziedzic K. Subsets of symptomatic hand osteoarthritis in community-dwelling older adults in the United Kingdom: prevalence, inter-relationships, risk factor profiles and clinical characteristics at baseline and 3-years. *Osteoarthritis and cartilage.* 2013;21(11):1674-1684:10.1016/j.joca.2013.08.004
4. Spacek E, Poiraudeau S, Fayad F, Lefevre-Colau MM, Beaudreuil J, Rannou F, et al. Disability induced by hand osteoarthritis: are patients with more symptoms at digits 2-5 interphalangeal joints different from those with more symptoms at the base of the thumb? *Osteoarthritis and cartilage.* 2004;12(5):366-373:10.1016/j.joca.2004.01.005
5. Kwok WY, Vliet Vlieland TP, Rosendaal FR, Huizinga TW, Kloppenburg M. Limitations in daily activities are the major determinant of reduced health-related quality of life in patients with hand osteoarthritis. *Annals of the rheumatic diseases.* 2011;70(2):334-336:https://doi.org/10.1136/ard.2010.133603
6. Hill S, Dziedzic KS, Ong BN. The functional and psychological impact of hand osteoarthritis. *Chronic Illn.* 2010;6(2):101-110:10.1177/1742395309345614
7. Kloppenburg M, Kroon FP, Blanco FJ, Doherty M, Dziedzic KS, Greibrokk E, et al. 2018 update of the EULAR recommendations for the management of hand osteoarthritis. *Ann Rheum Dis.* 2018:10.1136/annrheumdis-2018-213826
8. Tveter AT, Nossum R, Eide REM, Klokkeide Å, Matre KH, Olsen M, et al. OP0161 HPR Short-term effect of occupational therapy intervention on hand function and pain in patients with thumb based osteoarthritis - secondary analyses of a randomized controlled trial. *Ann Rheum Dis.* 2019;78(Suppl 2):155-156:10.1136/annrheumdis-2019-eular.5051
9. Gravas EMH, Tveter AT, Nossum R, Eide REM, Klokkeide A, Matre KH, et al. Non-pharmacological treatment gap preceding surgical consultation in thumb carpometacarpal osteoarthritis - a cross-sectional study. *BMC musculoskeletal disorders.* 2019;20(1):180:10.1186/s12891-019-2567-3
10. Gravås EMH, Østerås N, Nossum R, Eide REM, Klokkeide Å, Matre KH, et al. Does occupational therapy delay or reduce the proportion of patients that receives thumb carpometacarpal joint surgery? A multicentre randomised controlled trial. *RMD Open.* 2019;5(2):e001046:10.1136/rmdopen-2019-001046
11. Oppong R, Jowett S, Nicholls E, Whitehurst DG, Hill S, Hammond A, et al. Joint protection and hand exercises for hand osteoarthritis: an economic evaluation comparing methods for the analysis of factorial trials. *Rheumatology (Oxford, England).* 2015;54(5):876-883:10.1093/rheumatology/keu389
12. Kjeken I, Eide RE, Klokkeide A, Matre KH, Olsen M, Mowinckel P, et al. Does occupational therapy reduce the need for surgery in carpometacarpal osteoarthritis? Protocol for a randomized controlled trial. *BMC musculoskeletal disorders.* 2016;17(1):473:10.1186/s12891-016-1321-3
13. Herdman M, Gudex C, Lloyd A, Janssen M, Kind P, Parkin D, et al. Development and preliminary testing of the new five-level version of EQ-5D (EQ-5D-5L). *Qual Life Res.* 2011;20(10):1727-1736:10.1007/s11136-011-9903-x
14. Dolan P, Gudex C, Kind P, Williams A. The time trade-off method: results from a general population study. *Health Econ.* 1996;5(2):141-154:10.1002/(sici)1099-1050(199603)5:2<141::Aid-hec189>3.0.Co;2-n
15. Statens legemiddelverk. Guidelines for the submission of documentation for single technology assessment (STA) of pharmaceuticals.

For peer review only - http://bmjopen.bmj.com/site/about/guidelines.xhtml

https://legemiddelverket.no/Documents/English/Public%20funding%20and%20pricing/Documentation%20for%20STA/Guidelines%2020.05.2020.pdf. Published 2018. Accessed 8.6.21, 2021.

16. Helsedirektoratet. [DRG-systemet]. https://www.helsedirektoratet.no/tema/finansiering/innsatsstyrt-finansiering-og-drg-systemet/drg-systemet#omdrgsystemet%E2%80%8B. Accessed 8.6.21, 2021.

17. Statistics Norway. [*Salary*]. https://www.ssb.no/arbeid-og-lonn/statistikker/lonnansatt. Accessed 20.08.2020.

18. Arbeidstilsynet. [*Minimum wage*]. https://www.arbeidstilsynet.no/arbeidsforhold/lonn/minstelonn/. Accessed 20.08.2020.

19. Drummond MF, Sculpher MJ, Claxton K, Stoddart GL, Torrance GW. *Methods for the Economic Evaluation of Health Care Programmes.* Fourth edition ed. Oxford: Oxford University Press; 2015.

20. Norwegian Ministry of Health and Care Services. Principles for priority setting in health care. Summary of a white paper on priority setting in the Norwegian health care sector. In. https://www.regjeringen.no/contentassets/439a420e01914a18b21f351143ccc6af/en-gb/pdfs/stm201520160034000engpdfs.pdf2017.

21. Dziedzic KS, Hill S, Nicholls E, Hammond A, Myers H, Whitehurst T, et al. Self management, joint protection and exercises in hand osteoarthritis: a randomised controlled trial with cost effectiveness analyses. *BMC musculoskeletal disorders.* 2011;12:156:https://doi.org/10.1186/1471-2474-12-156

22. Ekman B, Nero H, Lohmander LS, Dahlberg LE. Costing analysis of a digital first-line treatment platform for patients with knee and hip osteoarthritis in Sweden. *PLoS One.* 2020;15(8):e0236342:10.1371/journal.pone.0236342

23. Sanders GD, Neumann PJ, Basu A, Brock DW, Feeny D, Krahn M, et al. Recommendations for Conduct, Methodological Practices, and Reporting of Cost-effectiveness Analyses: Second Panel on Cost-Effectiveness in Health and Medicine. *JAMA.* 2016;316(10):1093-1103:10.1001/jama.2016.12195

24. Gravås EMH, Østerås N, Nossum R, Mehl Eide RE, Klokkeide Å, Matre KH, et al. OP0160 HPR Does occupational therapy delay or reduce the proportion of patients who receive thumb carpometacarpal surgery? A randomized controlled trial. *Ann Rheum Dis.* 2019;78(Suppl 2):155-155:10.1136/annrheumdis-2019-eular.986

25. Tveter AT, Kleven L, Osteras N, Nossum R, Mehl Eide RE, Klokkeide Å, et al. OP0152-HPR a cost-utility analysis of multimodal occupational therapy in patients with thumb base osteoarthritis. *Ann Rheum Dis.* 2020;79(Suppl 1):96-96:10.1136/annrheumdis-2020-eular.3345

For peer review only

For peer review only - http://bmjopen.bmj.com/site/about/guidelines.xhtml

Figure legends

Supplementary figure A Study flow diagram of a 2-year study period in patients referred to surgical consultation due to carpometacarpal osteoarthritis.

Figure 1 Cost-effectiveness (CE) plane of the ICERs of multimodal occupational therapy (intervention group) compared to usual care (control group) were recalculated with 1 000 replications of the study data using the bootstrap resampling method. The intervention is at the origin.

Figure 2 shows a cost-effectiveness acceptability (CEA) curve for direct costs, representing the probability that the multimodal intervention is cost-effective compared to usual care. At a willingness-to-pay threshold of € 27 500/QALY, the probability of multimodal intervention being cost-effective was 94.5%.

For peer review only

For peer review only - http://bmjopen.bmj.com/site/about/guidelines.xhtml

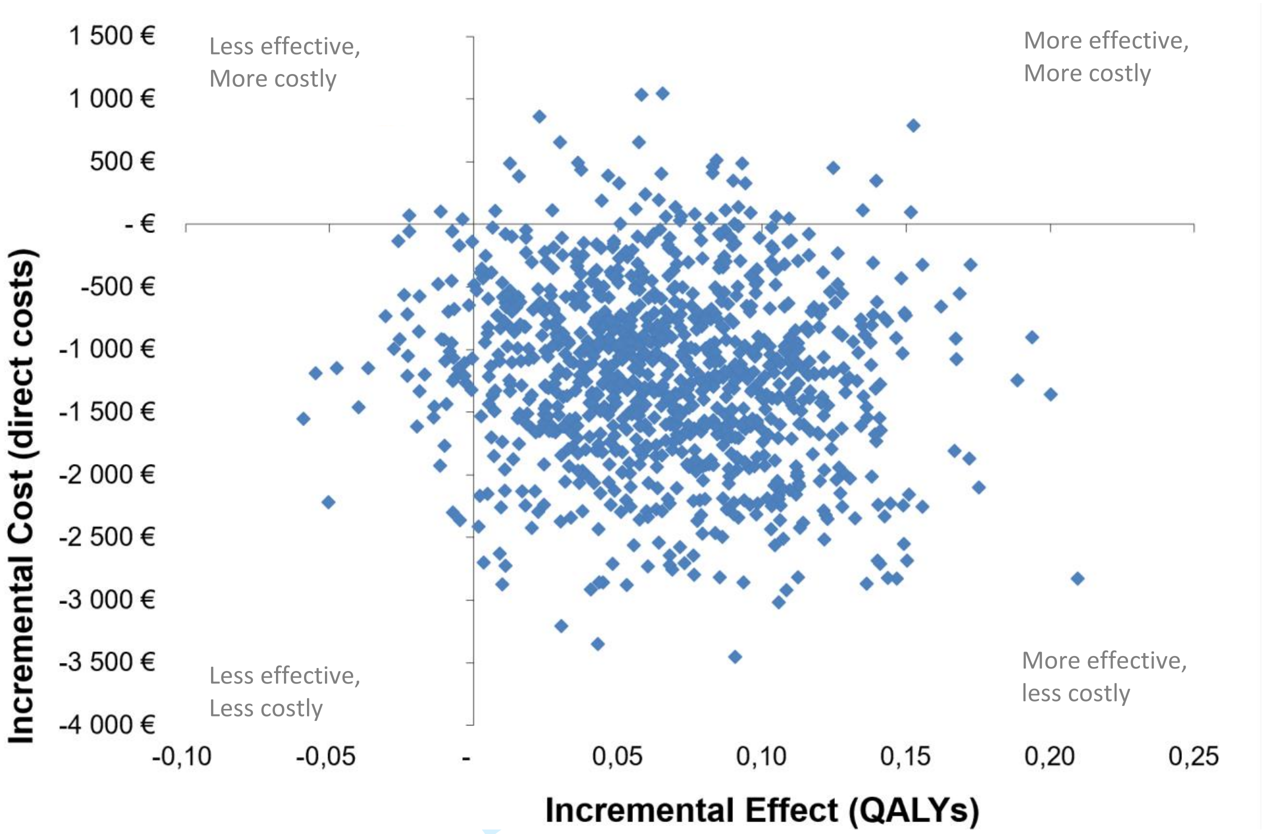
Less effective,
More costly
More effective,
More costly
Less effective,
Less costly
More effective,
less costly
1 500 €
1 000 €
500 €
- €
-500 €
-1 000 €
-1 500 €
-2 000 €
-2 500 €
-3 000 €
-3 500 €
-4 000 €
-0,10
-0,05
-
0,05
0,10
0,15
0,20
0,25
Incremental Cost (direct costs)
Incremental Effect (QALYs)

Figure 1

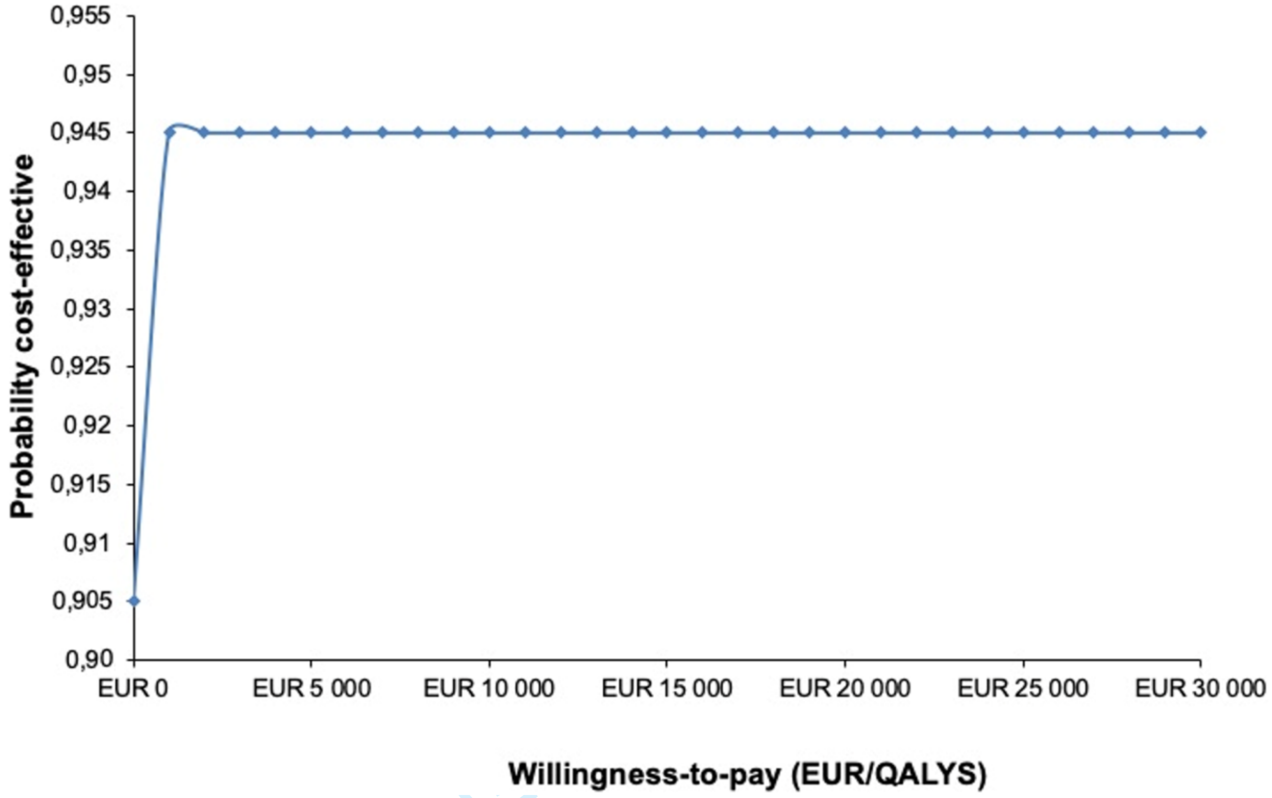

Figure 2

For peer review only

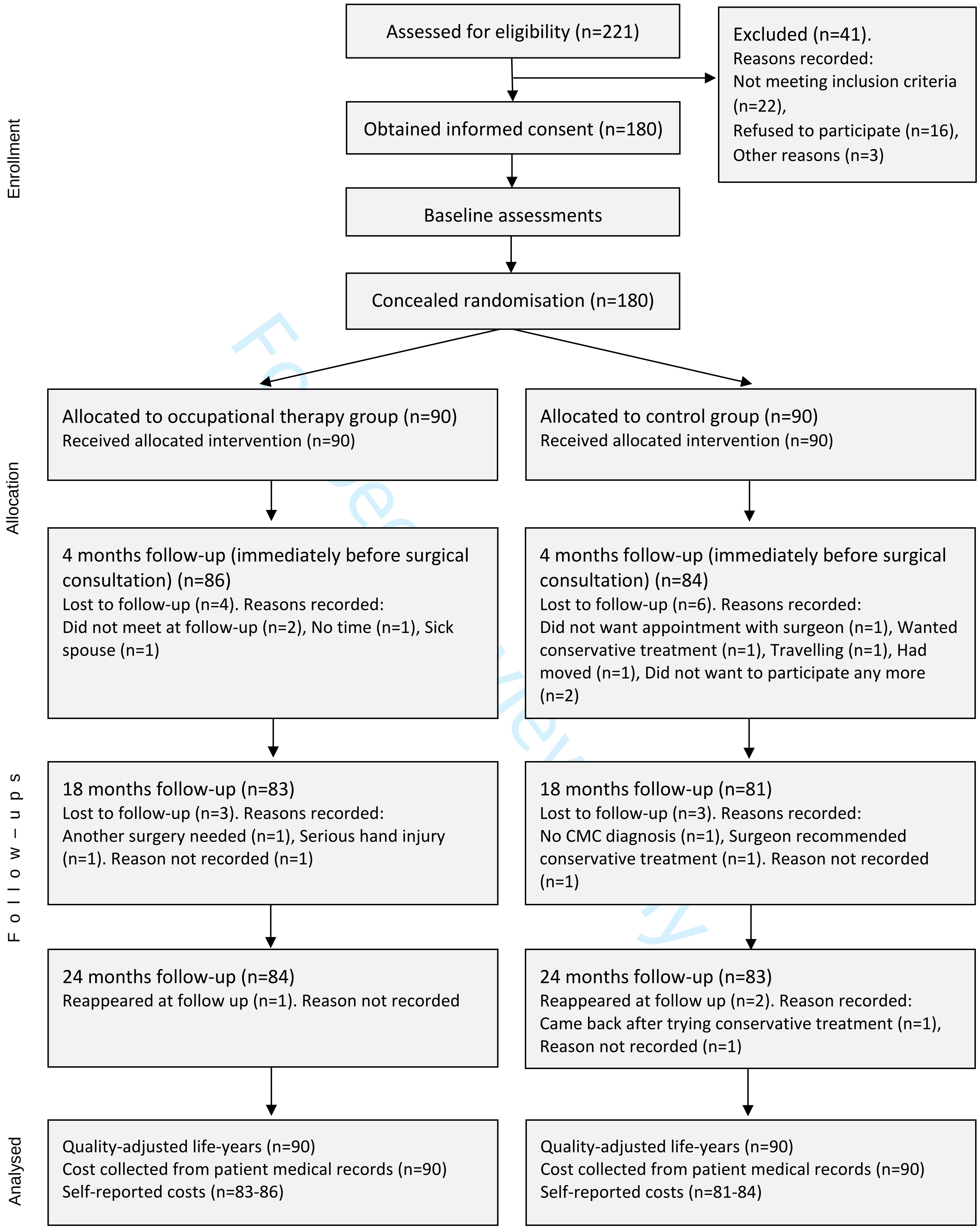

Supplementary figure A

CHEERS 2022 Checklist

**Title**			
Title	1	Identify the study as an economic evaluation and specify the interventions being compared.	page 1
**Abstract**			
Abstract	2	Provide a structured summary that highlights context, key methods, results, and alternative analyses.	pag 2
**Introduction**			
Background and objectives	3	Give the context for the study, the study question, and its practical relevance for decision making in policy or practice.	page 3-4
Methods			
**Health economic analysis plan**	**4**	**Indicate whether a health economic analysis plan was developed and where available.**	**page 3**
Study population	5	Describe characteristics of the study population (such as age range, demographics, socioeconomic, or clinical characteristics).	table 1
Setting and location	6	Provide relevant contextual information that may influence findings.	page 3-4
Comparators	7	Describe the interventions or strategies being compared and why chosen.	page 5
Perspective	8	State the perspective(s) adopted by the study and why chosen.	page 4
Time horizon	9	State the time horizon for the study and why appropriate.	page 4

For peer review only - http://bmjopen.bmj.com/site/about/guidelines.xhtml
For peer review only

(continued)

Discount rate	10	Report the discount rate(s) and reason chosen.	page 6-7
Selection of outcomes	11	Describe what outcomes were used as the measure(s) of benefit(s) and harm(s).	page 6-7
Measurement of outcomes	12	Describe how outcomes used to capture benefit(s) and harm(s) were measured.	page 6-7
Valuation of outcomes	13	Describe the population and methods used to measure and value outcomes.	page 4, page 6-7
Measurement and valuation of resources and costs	14	Describe how costs were valued.	page 7
Currency, price date, and conversion	15	Report the dates of the estimated resource quantities and unit costs, plus the currency and year of conversion.	table 2
Rationale and description of model	16	If modelling is used, describe in detail and why used. Report if the model is publicly available and where it can be accessed.	ua
Analytics and assumptions	17	Describe any methods for analysing or statistically transforming data, any extrapolation methods, and approaches for validating any model used.	page 7-8
Characterising heterogeneity	18	Describe any methods used for estimating how the results of the study vary for subgroups.	ua
Characterising distributional effects	19	Describe how impacts are distributed across different individuals or adjustments made to reflect priority populations.	ua

For peer review only - http://bmjopen.bmj.com/site/about/guidelines.xhtml

(continued)

Characterising uncertainty	20	Describe methods to characterise any sources of uncertainty in the analysis.	page 7
Approach to engagement with patients and others affected by the study	21	Describe any approaches to engage patients or service recipients, the general public, communities, or stakeholders (such as clinicians or payers) in the design of the study.	page 4
**Results**			
Study parameters	22	Report all analytic inputs (such as values, ranges, references) including uncertainty or distributional assumptions.	table 3, figure 1
Summary of main results	23	Report the mean values for the main categories of costs and outcomes of interest and summarise them in the most appropriate overall measure.	page 8-9, table 3
Effect of uncertainty	24	Describe how uncertainty about analytic judgments, inputs, or projections affect findings. Report the effect of choice of discount rate and time horizon, if applicable.	page 9-11
Effect of engagement with patients and others affected by the study	25	Report on any difference patient/service recipient, general public, community, or stakeholder involvement made to the approach or findings of the study	page 4
Discussion			

For peer review only - http://bmjopen.bmj.com/site/about/guidelines.xhtml

(continued)

Study findings, limitations, generalisability, and current knowledge	26	Report key findings, limitations, ethical or equity considerations not captured, and how these could affect patients, policy, or practice.	page 9-11
Other relevant information			
Source of funding	27	Describe how the study was funded and any role of the funder in the identification, design, conduct, and reporting of the analysis	page 13
Conflicts of interest	28	Report authors conflicts of interest according to journal or International Committee of Medical Journal Editors requirements.	page 13

From: Husereau D, Drummond M, Augustovski F, et al. Consolidated Health Economic Evaluation Reporting Standards 2022 (CHEERS 2022) Explanation and Elaboration: A Report of the ISPOR CHEERS II Good Practices Task Force. Value Health 2022;25. doi:10.1016/j.jval.2021.10.008

For peer review only

BMJ Open

Is multimodal occupational therapy cost-effective in addition to usual care only in people with carpometacarpal osteoarthritis: A cost-utility analysis of a randomized controlled trial

Journal:	*BMJ Open*
Manuscript ID	bmjopen-2022-063103.R1
Article Type:	Original research
Date Submitted by the Author:	20-Dec-2022
Complete List of Authors:	TVETER, Anne Therese; Diakonhjemmet Hospital, Center for treatment in rheumatic and musculoskeletal diseases (REMEDY), Department of Rheumatology and Research<br>Kleven, Linn; Norwegian Institute of Public Health<br>Osteras, Nina; Diakonhjemmet Hosp, Center for treatment in rheumatic and musculoskeletal diseases (REMEDY), Department of Rheumatology and Research<br>Nossum, Randi; Trondheim University Hospital<br>Eide, Ruth Else Mehl; Haukeland University Hospital<br>Klokkeide, Åse; Haugesund Rheumatism Hospital AS<br>Matre, Karin Hoegh; Haukeland University Hospital<br>Olsen, Monika; Haugesund Rheumatism Hospital AS<br>Kjeken, Ingvild; Diakonhjemmet Sykehus, Center for treatment in rheumatic and musculoskeletal diseases (REMEDY), Department of Rheumatology and Research
**Primary Subject Heading**:	Health economics
Secondary Subject Heading:	Rehabilitation medicine, Rheumatology
Keywords:	HEALTH ECONOMICS, Musculoskeletal disorders < ORTHOPAEDIC & TRAUMA SURGERY, Hand & wrist < ORTHOPAEDIC & TRAUMA SURGERY, RHEUMATOLOGY

SCHOLARONE™
Manuscripts

I, the Submitting Author has the right to grant and does grant on behalf of all authors of the Work (as defined in the below author licence), an exclusive licence and/or a non-exclusive licence for contributions from authors who are: i) UK Crown employees; ii) where BMJ has agreed a CC-BY licence shall apply, and/or iii) in accordance with the terms applicable for US Federal Government officers or employees acting as part of their official duties; on a worldwide, perpetual, irrevocable, royalty-free basis to BMJ Publishing Group Ltd ("BMJ") its licensees and where the relevant Journal is co-owned by BMJ to the co-owners of the Journal, to publish the Work in this journal and any other BMJ products and to exploit all rights, as set out in our licence.

The Submitting Author accepts and understands that any supply made under these terms is made by BMJ to the Submitting Author unless you are acting as an employee on behalf of your employer or a postgraduate student of an affiliated institution which is paying any applicable article publishing charge ("APC") for Open Access articles. Where the Submitting Author wishes to make the Work available on an Open Access basis (and intends to pay the relevant APC), the terms of reuse of such Open Access shall be governed by a Creative Commons licence – details of these licences and which Creative Commons licence will apply to this Work are set out in our licence referred to above.

Other than as permitted in any relevant BMJ Author's Self Archiving Policies, I confirm this Work has not been accepted for publication elsewhere, is not being considered for publication elsewhere and does not duplicate material already published. I confirm all authors consent to publication of this Work and authorise the granting of this licence.

For peer review only

Is multimodal occupational therapy cost-effective in addition to usual care only in people with carpometacarpal osteoarthritis: A cost-utility analysis of a randomized controlled trial

Anne Therese Tveter[1], Linn Kleven[2], Nina Østerås[1], Randi Nossum[3], Ruth Else Mehl Eide[4], Åse Klokkeide[5], Karin Hoegh Matre[4], Monika Olsen[5], Ingvild Kjeken[1]

[1] Center for treatment of rheumatic and musculoskeletal diseases (REMEDY), Diakonhjemmet Hospital, Oslo, Norway

[2] Norwegian Institute of Public Health, Oslo, Norway

[3] St.Olavs Hospital, Trondheim University Hospital, Trondheim, Norway

[4] Haukeland University Hospital, Bergen, Norway

[5] Haugesund Rheumatism Hospital AS, Haugesund, Norway

Corresponding author:

Anne Therese Tveter

Center for treatment of rheumatic and musculoskeletal diseases (REMEDY)

Diakonhjemmet Hospital

Postboks 23 Vinderen, 0319 Oslo, Norway

a.t.tveter@medisin.uio.no, https://orcid.org/0000-0003-1701-9835

For peer review only - http://bmjopen.bmj.com/site/about/guidelines.xhtml

ABSTRACT

Objective: The aim was to evaluate the cost-utility of a 3-month multimodal occupational therapy intervention in addition to usual care in patients with carpometacarpal osteoarthritis (OA).

Methods: A cost-utility analysis was performed alongside a multicentre randomized controlled trial including three rheumatology departments in Norway. A total of 180 patients referred to surgical consultation in specialist healthcare due to carpometacarpal OA were randomized to either multimodal occupational therapy including patient education, hand exercises, assistive devices, and orthoses (n=90), or usual care receiving only information on OA (n=90). The outcome measure was quality-adjusted life-years (QALYs) derived from the generic questionnaire EQ-5D-5L in an intention-to-treat population over a 2-year period. Resource use and health-related quality of life of the patients were prospectively collected at baseline, 4, 18, and 24 months. Costs were estimated by taking a healthcare and societal perspective. The results were expressed as incremental cost-effectiveness ratios (ICERs), and a probabilistic sensitivity analysis with 1 000 replications was done to account for uncertainty in the analysis.

Results: During the 2-year follow-up period, patients receiving multimodal occupational therapy gained 0.06 more QALYs than patients receiving usual care. The mean (SD) direct costs were € 3227 (3546) in the intervention group and € 4378 (5487) in the usual care group, mean difference €-1151 (95% CI -2564, 262). The intervention was the dominant treatment with a probability of 94.5% of being cost-effective given the willingness-to-pay threshold of € 27 500.

Conclusions: The within-trial analysis demonstrated that the multimodal occupational therapy was cost-effective in addition to usual care in patients with carpometacarpal osteoarthritis.

Trial registration number: ClinicalTrials.gov (NCT01794754)

For peer review only - http://bmjopen.bmj.com/site/about/guidelines.xhtml

Keywords: hand osteoarthritis, occupational therapy, cost-effectiveness, cost-utility, quality-adjusted life-years (QALYs)

Strength and limitations of the study

- A strength of the study is the involvement of a patient research partner throughout the conduct of the study
- Self-reported costs combined with several months between assessments may have biased the total costs of the study
- We were unable to include medication costs, travel expenses or purchase of technical or medical equipment in the analyses due to lack of or imprecise reporting.

INTRODUCTION

Hand osteoarthritis (OA) is the most common joint disease,[1] with the subcategory carpometacarpal osteoarthritis (CMC1 OA) affecting the base of the thumb.[2] Approximately 22% of people aged ≥50 years have symptomatic CMC1 OA.[3] Compared to OA in the other finger joints, CMC1 OA is associated with more pain and functional limitations, negatively affecting work ability and quality of life.[2,4,5]

There is a common misconception that hand OA is a normal part of aging, and that there is 'nothing to be done'.[6] Although there is no cure for OA, the European League Against Rheumatism (EULAR) recommends that all people with hand OA should be offered a multimodal intervention consisting of information, hand exercises, and assistive devices as first-line treatment.[7] In addition, orthoses should be provided for patients with CMC1 OA.[7] Pharmacological therapy is recommended as a symptom relieving supplement, whereas surgery should only be considered when other treatments have failed to provide sufficient pain relief.[7] The multimodal intervention is shown to have a significant short-term effect on pain[8,9] and hand function[9] compared to or in addition to usual care. However, there is an evidence-to-practice gap regarding this first-line treatment, with only 20% reporting to have received this treatment before being referred to surgical consultation.[10] This intervention also presents a tendency towards

For peer review only - http://bmjopen.bmj.com/site/about/guidelines.xhtml

For peer review only

reduction and delay in surgery rate over a 2-year period.[11] Although not significant, this tendency may still be of clinical importance if shown to be cost-effective.

Oppong et al,[12] performed a trial-based economic evaluation on joint protection only, hand exercises only and a combination of joint protection and hand exercises compared to leaflet and advice over a 12-month period in patients with hand OA. They found hand exercises to be the most cost-effective alternative.[12] In the study by Adams however, providing orthoses only was not found to be neither effective nor cost-effective.[13] To the best of our knowledge, no studies have assessed the cost-utility of a multimodal intervention following the EULAR recommendations for the treatment of hand OA or CMC1 OA.

The aim of this study was to perform a cost-utility analysis of a 3-month multimodal occupational therapy intervention in addition to usual care in patients with CMC1 OA referred to surgical consultation in specialist healthcare. The outcome measure was quality adjusted life-years (QALYs), and the costs were estimated both as direct and indirect costs and societal costs over a 2-year period taking a healthcare and societal perspective.

METHODS

Study design and setting

A cost-utility analysis (CUA) was performed alongside a multicentre RCT including rheumatology departments in three Norwegian hospitals (St. Olav's Hospital in Trondheim, Haukeland University Hospital in Bergen, and Haugesund Rheumatism Hospital) and is described in the protocol article.[11,14] In Norway, most hospitals (including the three hospitals involved in this trial) are public and owned by the Norwegian government. The trial was approved by the Norwegian Regional Ethical Committee (2012/2265/REK sør-øst C).

Patient and Public Involvement

A patient research partner from the Patient Research Panel at Diakonhjemmet Hospital with experienced knowledge on hand osteoarthritis was involved throughout the project. She participated in

For peer review only - http://bmjopen.bmj.com/site/about/guidelines.xhtml

project meetings where she gave input to the project plan, recruitment procedure, and information material as well as feedback on the interpretation and dissemination of the results.

Participants and randomization

Patients referred by their general practitioner for surgical consultation due to CMC1 OA at the three rheumatology departments between 2013 and 2015 were considered eligible for the study if they could speak Norwegian and had no cognitive deficits. Eligible patients received information about the study, a written consent form and a questionnaire by post. Patients who agreed to participate were scheduled for an appointment with an occupational therapist (OT) at the hospital within 2 weeks after referral. After a baseline assessment, the patients were randomized to either a 3-month multimodal occupational therapy intervention or usual care. In Norway, the waiting period between referral and the actual surgical consultation was at the time this study was conducted between 4 and 6 months, and the intervention was conducted during this waiting period. Patients were assessed again immediately before surgical consultation (approximately 4 months after baseline assessment), and 18 and 24 months after the baseline assessment (Supplementary figure A). The sample size of 180 participants was estimated based on an expected surgery rate of 70% during the 2-year follow-up, which was the primary endpoint of the RCT.[11,14]

Intervention

During the waiting period between referral and surgical consultation, the intervention group received a multimodal occupational therapy intervention in line with recommended first-line treatment.[7] At the baseline assessment, the patients received written and oral information on hand OA, ergonomic principals, and the use of assistive devices. They were instructed in a hand exercise program to be performed at home three times per week for 12 weeks. They all received five common assistive devices (a bread knife and vegetable knife with built-up handles, an enlarged grip for opening bottles, a key for opening jars [the Jar Key], and a self-opening pair of scissors) for use when needed, and daytime and nighttime orthoses that they were encouraged to use as much as possible.[14] The patients in the

For peer review only - http://bmjopen.bmj.com/site/about/guidelines.xhtml

intervention group consulted the OT twice during this intervention period: at the baseline assessment and again after 2 weeks to adjust the orthoses and exercise program, implying that the intervention was based mostly on self-management. Patients referred for bilateral hand OA received treatment for both hands. At the follow-up assessments (4, 18, and 24 months), the patients in the intervention group could receive new orthoses, or the OTs could adjust the ones they already had.

The control group received usual care, which generally means staying on the waiting list for consultation in specialist healthcare, i.e., receiving no treatment in specialist healthcare. They may however have been seeking treatment by themselves in primary healthcare (this information was collected as part of this study).

For the purpose of this study, the patients in both groups also received written and oral information on hand OA at the baseline assessment. Both groups had a surgical consultation with a medical specialist approximately 4 months after the baseline assessment and were assessed by an OT at all follow-ups. If necessary, patients in both groups could seek additional treatment, such as a consultation with general practitioner or other healthcare personnel. The initial assessment and the 2-week follow-up in the intervention group were regarded as part of the care plan, while the subsequent follow-up assessments were performed as part of study data collection.

Randomization and blinding

Patients were randomized using a computer-generated list with a block size of 10, stratified by hospital. Envelopes were opened by the patients after receiving information on hand OA and completing baseline assessment. The group affiliation was known to both the patients and the OTs.

Data collection

Health outcome

The health-related quality of life (HRQoL) was measured at baseline, 4, 18, and 24 months using the generic instrument EQ-5D-5L (EuroQol 5-dimension, www.euroqol.org).[15] The EQ-5D-5L is a preference weighted measure of HRQoL based on five dimensions: mobility, self-care, daily activities, pain/discomfort,

For peer review only - http://bmjopen.bmj.com/site/about/guidelines.xhtml

and anxiety/depression. For each dimensions the patient assesses five possible levels of problems, from no problems to severe.[15] Quality-adjusted life-years (QALYs) were derived from the EQ-5D-5L utility score, using the preference score from a UK population. Since there is no Norwegian tariff, the recommendation in Norway is to use the British tariff.[16] QALYs are weighted for time (years of life) with an index that express the HRQoL (utilities) on a scale anchored at 0 (health state equivalent to death) and 1 QALY (full health).

Resource use and costs

Healthcare utilization was collected from different sources at baseline, 4, 18 and 24 months. The OT reported the number of consultations related to the intervention at each assessment, whereas surgical procedures and post-operative treatment were collected from patients' medical record. In addition, the use of primary and specialist healthcare was self-reported by the patient. We have included the costs related to the OT assessment at baseline, 4, and 18 months for both groups (OT assessment at 24 months were not included as the usual care group then received the intervention). Although the OT assessment was done for study purposes, it was included in the cost estimates because we cannot rule out a possible positive effect of an appointment with an OT in specialist healthcare for the control group. We have not included medication costs, travelling expenses or purchase of technical or medical equipment (except assistive devices provided to patients in the intervention group) due to lack of or imprecise reporting.

Data on sickness absence related to surgery were collected from patient medical records. The number of hours of informal help at home due to OA (from spouse, family, or friends) and the productivity loss (due to any reason of sickness absence and disability benefits, excluding sickness absence related to surgery) were self-reported. If not explicitly indicated by the patient, we expected that the productivity loss remained the same between assessment points, thus, if the patient reported sickness absence of 50% at 4 months and 100% at 18 months, this was calculated as 50% productivity loss between 4 and 18 months, and 100% between 18 and 24 months.

The costs of specialist healthcare visits and surgery were calculated using DRG weights (diagnosis-related group) from 2019. DRG is a coding system used for administration of both clinical and financial activity in specialist healthcare. The DRG costs were estimated by multiplying the cost weighting for the

For peer review only - http://bmjopen.bmj.com/site/about/guidelines.xhtml

specific DRG group of the patient with the unit cost of 2019.[17] Sickness absence and disability benefits were valued in the same way for all patients. Costs related to absence from work were estimated from official statistics on the average wage by sex and age group in 2019. The average daily productivity loss was valued as € 336 including societal costs of 40% and was adjusted for the percentage of sickness absence/disability benefits.[18] The number of hours of informal help at home was valued as a minimum wage of € 18 per hour.[19] All costs are estimated in Euros, using the exchange rate 0.10 (€ 1 = NOK 10) from 2019.

Direct costs included costs related to specialist healthcare (medical specialist, occupational therapist, physical therapist, nurse, radiographs, surgery, assistive devices) and primary healthcare (general practitioner, physical therapist, manual therapist, acupuncturist, blood samples). Indirect costs included productivity loss due to sickness absence (due to surgery or for other reasons) and costs related to help with chores at home.

Statistics

Differences in QALYs between the two groups were estimated using the trapezoidal method (area under the curve).[20] To illustrate the statistical uncertainty surrounding the incremental cost-effectiveness ratio (ICER), a probabilistic sensitivity analysis with 1 000 replications was performed using the bootstrap resampling method. The bootstrapped costs and effects can be illustrated on a cost-effectiveness plane (CE plane). The CE plane (Figure 1) are divided into four quadrants; where a new treatment that is more effective and less costly is plotted in the southeast quadrant, while one more costly and less effective is plotted in the northwest quadrant. Figure 2 shows the cost-effectiveness acceptability curve (CEAC), which presents the probability that the intervention is cost-effective in addition to usual care for hand OA. The willingness-to-pay threshold was set to € 27 500 (275 000 NOK) due to the low severity of the condition.[21]

Between-group differences in mean costs and mean effects were analyzed using Independent sample t-test. Significance level was set to 0.05. Statistical analyses were performed using Excel, SPSS version 26 and Stata version 16.

Missing data

For peer review only

A total of 18 patients (7 in the intervention group and 11 in the control group) had missing HRQoL utility score at one or more timepoints. For these missing values we used 'last observation carried forward'. Sensitivity analyses showed that the average HRQoL utility scores remained the same for both groups without imputation of these missing values. Missing self-reported costs were not imputed (n=16). The costs related to surgery were collected from patient medical records and were not encumbered by missing data.

RESULTS

Baseline characteristics

A total of 180 patients were randomized to either multimodal occupational therapy (n=90) or usual care (n=90). The mean age across both groups was 63 years (SD 7.6) and 79% of the patients were women. We found no between-group differences in any of the baseline characteristics including utility score, medication use, or percentage of patients working full- or part-time (Table 1).

For peer review only

Table 1 Demographic variables for patients referred to surgical consultation due to carpometacarpal osteoarthritis (n = 180). All information was collected at the baseline assessment

		Intervention group (n=90)	Control group (n=90)
Age, years, mean (SD)		62.8 (7.5)	63.3 (7.8)
Female sex, *n* (%)		73 (81)	69 (77)
Referred hand, *n* (%)			
	Right	31 (34)	17 (19)
	Left	24 (27)	29 (32)
	Both	35 (39)	44 (49)
Education level, *n* (%)			
	Primary school	14 (16)	20 (22)
	High school	44 (49)	39 (43)
	University/college ≤ 4 y	17 (19)	19 (21)
	University/college > 4 y	15 (16)	12 (13)
Living alone, *n* (%)		17 (19)	18 (20)
Working full/part-time, *n* (%)		48 (53)	43 (48)
Years with symptoms, median (IQR)‡		5 (2–10)	5 (2–10)
Presence of subluxation of the CMC joint		58 (64)	55 (61)
Comorbidity, *n* (%)		61 (68)	56 (62)
Analgesics use (anti-inflammatory or pain relief medication), *n* (%)		57 (63)	57 (63)
Pain at rest (NRS 0–10, 0 = no pain), median (IQR)		3 (1–4)	3 (2–5)
EQ-5D-5L utility score (1=best health), mean (SD)		0.74 (0.16)	0.73 (0.18)

SD= standard deviation; IQR= interquartile range (25th and 75th percentile); CMC= carpometacarpal joint; NRS= numeric rating scale; Pain at rest was reported for referred hand (the mean of both hands if bilateral referral)

Cost-utility analysis

The total between-group difference in QALYs was 0.06 (95% CI -0.02, 0.15) after 24 months, in favor of the intervention group. The distribution of EQ-5D-5L utility score across the different time points for the two groups are shown in Supplementary figure B. The mean healthcare costs and productivity loss for both treatment groups are presented in Table 2.

For peer review only - http://bmjopen.bmj.com/site/about/guidelines.xhtml
For peer review only

Table 2 Mean direct and indirect healthcare costs (mean and standard deviation) per patient for the intervention and control group (n=180). The table also show unit price and the resource use (n).

		Unit	Unit price, €	n	Intervention group Mean (SD) per patient, €	n	Control group Mean (SD) per patient, €	Mean difference (95% CI), €
Direct costs	*Specialist healthcare*							
	Medical specialist	Per visit	205[a]	136	309 (268)	169	384 (365)	-75 (-169, 19)
	Occupational therapist including adaptation of orthoses	Per visit	246[a]	161	536 (318)	5	158 (367)	377 (275, 478)
	Occupational / Physical therapist	Per visit	205[a]	169	428 (350)	152	837 (866)	-355 (-549, -160)
	Nurse	Per visit	205[a]	1	16 (98)	9	48 (189)	-32 (-76, 13)
	Inpatient surgery	Per surgery	8 435[a]	2	187 (1 250)	6	562 (2 465)	-375 (-951, 201)
	Day-bed surgery	Per surgery	2 251[a]	20	500 (941)	27	675 (1 190)	-175 (-491, 141)
	Radiographs	Per visit	149 [e]	90	149 (0)	90	149 (0)	-
	Assistive devices	Per person	67 [e]	90	67 (0)	0	-	-
	Primary healthcare							
	General practitioner	Per visit	32[b]	91	32 (94)	130	46 (104)	-14 (-43, 15)
	Physical therapist	Per visit	31[c]	187	64 (288)	158	54 (160)	10 (-59, 79)
	Manual therapist	Per visit	52[c]	0	-	16	10 (90)	-
	Acupuncturist	Per visit	47[e]	22	11 (64)	44	23 (132)	-11 (-42, 19)
	Blood samples	Per test	7[b]	14	1 (6)	24	2 (6)	-1 (-2, 1)
Indirect costs	Sickness absence due to surgery	Days	336[d]	65	1 212 (3 815)	64	1 199 (3 817)	186 (-1 104, 1 141)
	Costs related to help with chores at home	Hours	18[d]	3261	652 (1 722)	4612	922 (3 089)	-270 (-1 006, 465)
	Sickness absence and disability benefits (not sickness absence due to surgery)	Days	336[d]	10351	38 299 (63 029)	12 249	45 433 (66 320)	-7 133 (-26 167, 1 190)

For peer review only

[a] DRG (diagnosis-related group system); [b] List of reimbursement codes; [c] Rates for physiotherapists; [d] Official statistics of average wage; [e] Based on real costs. All costs are given in Euro (€1=NOK10) and adjusted to 2019.

For peer review only - http://bmjopen.bmj.com/site/about/guidelines.xhtml

The mean cost of multimodal occupational therapy intervention (baseline information on hand OA, instruction in hand exercises, assistive devices, and customization of orthoses, and adjustment of hand exercises and orthoses after 14 days) was approximately € 500 per patient. Surgery accounted for the largest between-group difference with 22 surgical procedures in the intervention group and 33 surgical procedures in the control group, resulting in a €550 difference in surgical cost per patient between the two groups. The use of physiotherapy/occupational therapy also constituted a high cost. However, almost one-third of these costs were related to the predetermined assessment points (4, 18 and 24 months) that were part of the study but not the actual OT intervention (€375 per patient in the control group and €307 per patient in the intervention group).

Productivity loss due to sickness absence or disability benefits was the largest contributor to the total costs in both groups. However, at baseline, only 7% of the sickness absence in both groups was reported to be due to hand OA, and the percentages at 4, 18, and 24 months were 6%, 2% and 0.5%, respectively. Similar numbers of patients in the two groups were retired at baseline (34% in the control group versus 32% in the intervention group), 4 months (36% versus 34%), and 24 months (42% versus 43%). However, more patients in the intervention group (43%) retired between 4 and 18 months than in the control group (36%), indicating that fewer patients contributed to productivity loss in the intervention group during this period.

Table 3 shows the mean incremental costs and incremental effects associated with direct and indirect healthcare costs. In the case of a negative ICER, the evaluated treatment is either dominant (more effective and less costly) or dominated (less effective and more costly). The intervention in this study was the dominant treatment (Figure 1) with a 94.5% probability of being cost-effective given the willingness-to-pay threshold (Figure 2), indicating that the intervention was both less costly and more effective in addition to usual care.

For peer review only - http://bmjopen.bmj.com/site/about/guidelines.xhtml

Table 3 Cost-utility analysis of mean costs (SD), mean effects (SD), incremental differences in costs and effects (95% CI), and incremental cost-effectiveness ratio (ICER) for direct and total direct and indirect costs (n=180)

	**Intervention group (n=90)**		**Control group (n=90)**				
	**Mean cost (SD)**	**Mean effect* (SD)**	**Mean cost (SD)**	**Mean effect* (SD)**	**Incremental cost (95% CI)**	**Incremental effect (95% CI)**	**ICER**
Direct costs	3 227 (3 546)	1.58 (0.28)	4 378 (5 487)	1.51 (0.28)	-1 151 (-2 564, 262)	0.06 (-0.02, 0.15)	Dominant
Total costs (direct and indirect costs)	46 617 (65 499)	1.58 (0.28)	56 304 (68 872)	1.51 (0.28)	-9 688 (-9 949, 29 324)	0.06 (-0.02, 0.15)	Dominant

Direct costs, **€**: self-reported use of primary and specialist healthcare, including surgery;

Indirect costs, **€**: productivity loss from sickness absence due to surgery, costs related to informal help at home, productivity loss from sickness absence and disability benefits excluding sickness absence due to surgery

Total cost, **€**: direct costs + indirect costs

* Mean effect without imputations; Intervention group 1.58 (0.28), Control group 1.51 (0.28)

SD= standard deviation

For peer review only

For peer review only - http://bmjopen.bmj.com/site/about/guidelines.xhtml

DISCUSSION

The aim of this study was to evaluate the cost-utility of a 3-month multimodal occupational therapy intervention in patients with CMC1 OA in a 2-year period. Our results demonstrate that the intervention, consisting of patient education, hand exercises, orthoses, and assistive devices, has the highest probability of being cost-effective, given the willingness-to-pay threshold (€ 27 500/QALY). This study is one of few studies to evaluate the cost-utility of conservative treatment in hand OA, and the only study to evaluate the cost-utility of the 2018 EULAR guidelines on first-line treatment for CMC1 OA. Our findings add to the evidence base by demonstrating that multimodal occupational therapy is a cost-effective alternative for the management of CMC1 OA.

The results of this study are in line with a previous study showing that hand exercises were more cost-effective than leaflet and advice in patients with hand OA.[12] In contrast, the study by Oppong et al. failed to show that the combination of hand exercises and use of assistive devices (joint protection classes[22]) were more cost-effective than hand exercises alone.[12] Similarly, the use of orthoses alone was not shown to be neither effective nor cost-effective.[13] In our study, we did not separately evaluate the different parts of the recommended first-line treatment. It is possible that we would have found the same results as Oppong et al.,[12] given that hand exercises only entail costs related to the time used by health personnel to educate the patient, whereas both orthoses and assistive devices require an additional cost. On the other hand, the combination of the recommended first-line treatment was found to have a significant short-term effect on reducing pain and improving hand function in these patients,[8] and should be offered to all patients with CMC1 OA as an effective and cost-effective alternative. Due to the self-managing nature of the intervention, the mean cost related to the multimodal occupational therapy intervention was low, approximately €500 per patient. However, the current study was conducted in specialist healthcare, and there are other options for delivering this intervention that may be even more cost-effective. The treatment of patients with OA should mainly be conducted in primary healthcare, possibly reducing the costs to some extent. However, due to the lack of availability of treatment options and long travel distances in parts of the country, more cost-effective options for delivery of treatment could be further explored, e.g.

For peer review only

For peer review only - http://bmjopen.bmj.com/site/about/guidelines.xhtml

using a smart phone application as shown in a Swedish study on digital first-line treatment for hip and knee OA.[23] We are currently evaluating the effect and cost-utility of a newly developed smartphone app (the Happy Hands app) which is designed to make recommended treatment available to people with hand OA.[24]

Surgery constituted a large cost in this within-trial. Even if we found a tendency towards reduction in surgical procedures in the intervention group compared to the control group, this result was not statistically significant.[11] Still, the between-group difference in mean surgical costs (€ 550) was higher than the mean cost of the multimodal occupational therapy intervention alone (€ 500). Thus, although the difference in surgical rates between the two groups were found to be non-significant,[11] the difference may still be regarded as important in a healthcare and societal perspective as surgical procedures may be prone to more adverse events compared to multimodal occupational therapy.[11] Most likely, the avoidance of surgery has the highest impact on the cost-effectiveness of the intervention.

The patients reported to have OA complaints for a median of 5 years, and more than 60% showed radiographic signs of subluxation of the carpometacarpal joint, indicating advanced disease progression. However, only 21% of the patients had visited a physiotherapist or an OT before referral to surgical consultation.[10] Thus, referral to a multimodal occupational therapy intervention at an earlier stage in the disease course may be a possible step towards reducing the costs related to healthcare use.

The current study is the first study assessing the cost-utility of recommended multimodal treatment in patients with CMC1 OA. It is considered a strength that the study was conducted alongside a well-designed RCT. The study also has some limitations. Almost 80% of the sample were women, which represents a higher proportion of women than the usual gender distribution in hand OA. The results may therefore not be representative for men with hand OA. The self-reported costs and the 14-month time frame between the reporting may have biased the total costs. This has most likely contributed to lower overall costs, as patients may not remember to report all consultations with healthcare personnel, especially in the time period between 4 and 18 months. On the other hand, the costs related to surgery were collected from patient medical records and will not be encumbered by the same bias. A total of 16 patients had missing data throughout the study. There may have been systematic reasons for these

For peer review only - http://bmjopen.bmj.com/site/about/guidelines.xhtml

patients being missing, which could have affected the outcome. However, there were more missing data in the control group than the intervention group, indicating that the between-group difference could have been larger if all patients had reported direct and indirect costs. The results may have been biased the method of imputation of missing HRQoL utility values. However, due to low number of missing values and sensitivity analyses showing no difference in mean score whether using 'Last observation carried forward' or not, we did not go any further with more advanced methods like multiple imputation. We were unable to include medication costs, travel expenses or purchase of technical or medical equipment in the analyses. This is not in line with the most recent recommendations for cost-utility analyses[25] and may have biased the results by possibly underestimating the total costs. However, the equal distribution of medication use across the two groups imply that this do not constitute a large bias in the comparison of results. As with all conservative treatments, the patients knew that they received the intervention, and this may be a potential bias improving the effect measure. Measurement properties of the EQ-5D-5L has not been assessed in patients with CMC OA. The HRQoL measure might also capture QoL related to other conditions than only CMC OA. Although the follow-up sessions were similar for both groups, the results may have been influenced by the fact that patients in the intervention group could receive adjustment of orthoses or new orthoses at these sessions. This may have influenced the results. The involvement of a patient research partner with lived experience on hand osteoarthritis is a strength, however, the study would have benefited from input from more than one partner. Future studies should evaluate the cost-utility of an even longer follow-up time as there is a lack of knowledge on surgical rate of these patients in the long term.

CONCLUSION

The within-trial analysis demonstrated that multimodal occupational therapy was cost-effective in addition to usual care in patients with CMC1 OA, when taking a healthcare and societal perspective. The intervention was the dominant treatment with a 94.5%, probability of being cost-effective, given the

For peer review only

willingness-to-pay threshold of € 27 500/QALY. The low cost related to the intervention supports the use of this first-line treatment in all patients with CMC1 OA.

Acknowledgements

We would like to acknowledge patient research partner Øyvor Andreassen for her contribution throughout the project. We would also like to thank professor Tore K. Kvien for reading and commenting on the manuscript. The project has been acknowledged at the EULAR Congress with the HPR Abstract Award in 2019 for the primary outcome results and the HPR Abstract Award in 2020 for the results presented in this article.

Author contribution

Ingvild Kjeken was responsible for obtaining funding. Randi Nossum, Ruth Else Mehl Eide, Åse Klokkeide, Karin Hoegh Matre, and Monika Olsen contributed to data acquisition. Anne Therese Tveter, Linn Kleven, Nina Østerås, and Ingvild Kjeken were responsible for data analysis and interpretation. Further, all authors have made substantial contributions to the study conception and design, drafting of the article, critical revision of the article for important intellectual content and final approval of the submitted version.

Funding statement

This work was supported by the South-Eastern Norway Regional Health Authority (2015109) and Norges Forskningsråd (328657). The funding authorities had otherwise no involvement in the study. This research received no specific grant from any funding agency in the public, commercial or not-for-profit sector.

Conflict of interest

None of the authors have any financial or personal conflicts of interest.

Data sharing statement

For peer review only

For peer review only - http://bmjopen.bmj.com/site/about/guidelines.xhtml

Data may be made available upon reasonable request to the project manager (Ingvild Kjeken, Ingvild.kjeken@diakonsyk.no)

Ethics Statement

The trial was approved by the Norwegian Regional Ethical Committee (2012/2265/REK sør-øst C). All participants gave a written informed consent before taking part in the study.

For peer review only

For peer review only - http://bmjopen.bmj.com/site/about/guidelines.xhtml

Figure legends

Supplementary figure A Study flow diagram of a 2-year study period in patients referred to surgical consultation due to carpometacarpal osteoarthritis.

Supplementary figure B EQ-5D-5L utility score across the different timepoints, divided by group.

Figure 1 Cost-effectiveness (CE) plane of the ICERs of multimodal occupational therapy (intervention group) in addition to usual care (control group) were recalculated with 1 000 replications of the study data using the bootstrap resampling method. The intervention is at the origin.

Figure 2 shows a cost-effectiveness acceptability (CEA) curve for direct costs, representing the probability that the multimodal intervention is cost-effective in addition to usual care. At a willingness-to-pay threshold of € 27 500/QALY, the probability of multimodal intervention being cost-effective was 94.5%.

For peer review only

References

1. Marshall M, Watt FE, Vincent TL, Dziedzic K. Hand osteoarthritis: clinical phenotypes, molecular mechanisms and disease management. *Nature Reviews Rheumatology.* 2018;14(11):641-656:10.1038/s41584-018-0095-4
2. Bijsterbosch J, Visser W, Kroon HM, Stamm T, Meulenbelt I, Huizinga TW, et al. Thumb base involvement in symptomatic hand osteoarthritis is associated with more pain and functional disability. *Annals of the rheumatic diseases.* 2010;69(3):585-587:10.1136/ard.2009.104562
3. Marshall M, Peat G, Nicholls E, van der Windt D, Myers H, Dziedzic K. Subsets of symptomatic hand osteoarthritis in community-dwelling older adults in the United Kingdom: prevalence, inter-relationships, risk factor profiles and clinical characteristics at baseline and 3-years. *Osteoarthritis and cartilage.* 2013;21(11):1674-1684:10.1016/j.joca.2013.08.004
4. Spacek E, Poiraudeau S, Fayad F, Lefevre-Colau MM, Beaudreuil J, Rannou F, et al. Disability induced by hand osteoarthritis: are patients with more symptoms at digits 2-5 interphalangeal joints different from those with more symptoms at the base of the thumb? *Osteoarthritis and cartilage.* 2004;12(5):366-373:10.1016/j.joca.2004.01.005
5. Kwok WY, Vliet Vlieland TP, Rosendaal FR, Huizinga TW, Kloppenburg M. Limitations in daily activities are the major determinant of reduced health-related quality of life in patients with hand osteoarthritis. *Annals of the rheumatic diseases.* 2011;70(2):334-336:https://doi.org/10.1136/ard.2010.133603
6. Hill S, Dziedzic KS, Ong BN. The functional and psychological impact of hand osteoarthritis. *Chronic Illn.* 2010;6(2):101-110:10.1177/1742395309345614
7. Kloppenburg M, Kroon FP, Blanco FJ, Doherty M, Dziedzic KS, Greibrokk E, et al. 2018 update of the EULAR recommendations for the management of hand osteoarthritis. *Ann Rheum Dis.* 2018:10.1136/annrheumdis-2018-213826
8. Tveter AT, Østerås N, Nossum R, Eide REM, Klokkeide Å, Hoegh Matre K, et al. Short-term effects of occupational therapy on hand function and pain in patients with carpometacarpal osteoarthritis: secondary analyses from a randomized controlled trial. *Arthritis care & research.* 2020:10.1002/acr.24543
9. Deveza LA, Robbins SR, Duong V, Bennell KL, Vicenzino B, Hodges PW, et al. Efficacy of a Combination of Conservative Therapies vs an Education Comparator on Clinical Outcomes in Thumb Base Osteoarthritis: A Randomized Clinical Trial. *JAMA Intern Med.* 2021;181(4):429-438:10.1001/jamainternmed.2020.7101
10. Gravas EMH, Tveter AT, Nossum R, Eide REM, Klokkeide A, Matre KH, et al. Non-pharmacological treatment gap preceding surgical consultation in thumb carpometacarpal osteoarthritis - a cross-sectional study. *BMC musculoskeletal disorders.* 2019;20(1):180:10.1186/s12891-019-2567-3
11. Gravås EMH, Østerås N, Nossum R, Eide REM, Klokkeide Å, Matre KH, et al. Does occupational therapy delay or reduce the proportion of patients that receives thumb carpometacarpal joint surgery? A multicentre randomised controlled trial. *RMD Open.* 2019;5(2):e001046:10.1136/rmdopen-2019-001046
12. Oppong R, Jowett S, Nicholls E, Whitehurst DG, Hill S, Hammond A, et al. Joint protection and hand exercises for hand osteoarthritis: an economic evaluation comparing methods for the analysis of factorial trials. *Rheumatology (Oxford, England).* 2015;54(5):876-883:10.1093/rheumatology/keu389
13. Adams J, Barratt P, Rombach I, Arden N, Barbosa Bouças S, Bradley S, et al. The clinical and cost effectiveness of splints for thumb base osteoarthritis: a randomized controlled clinical trial. *Rheumatology (Oxford, England).* 2021;60(6):2862-2877:10.1093/rheumatology/keaa726
14. Kjeken I, Eide RE, Klokkeide A, Matre KH, Olsen M, Mowinckel P, et al. Does occupational therapy reduce the need for surgery in carpometacarpal osteoarthritis? Protocol for a randomized controlled trial. *BMC musculoskeletal disorders.* 2016;17(1):473:10.1186/s12891-016-1321-3
15. Herdman M, Gudex C, Lloyd A, Janssen M, Kind P, Parkin D, et al. Development and preliminary testing of the new five-level version of EQ-5D (EQ-5D-5L). *Qual Life Res.* 2011;20(10):1727-1736:10.1007/s11136-011-9903-x

For peer review only - http://bmjopen.bmj.com/site/about/guidelines.xhtml

16. Statens legemiddelverk. Guidelines for the submission of documentation for single technology assessment (STA) of pharmaceuticals. https://legemiddelverket.no/Documents/English/Public%20funding%20and%20pricing/Documentation%20for%20STA/Guidelines%2020.05.2020.pdf. Published 2018. Accessed 8.6.21, 2021.
17. Helsedirektoratet. [DRG-systemet]. https://www.helsedirektoratet.no/tema/finansiering/innsatsstyrt-finansiering-og-drg-systemet/drg-systemet#omdrgsystemet%E2%80%8B. Accessed 8.6.21, 2021.
18. Statistics Norway. [*Salary*]. https://www.ssb.no/arbeid-og-lonn/statistikker/lonnansatt. Accessed 20.08.2020.
19. Arbeidstilsynet. [*Minimum wage*]. https://www.arbeidstilsynet.no/arbeidsforhold/lonn/minstelonn/. Accessed 20.08.2020.
20. Drummond MF, Sculpher MJ, Claxton K, Stoddart GL, Torrance GW. *Methods for the Economic Evaluation of Health Care Programmes.* Fourth edition ed. Oxford: Oxford University Press; 2015.
21. Norwegian Ministry of Health and Care Services. Principles for priority setting in health care. Summary of a white paper on priority setting in the Norwegian health care sector. In. https://www.regjeringen.no/contentassets/439a420e01914a18b21f351143ccc6af/en-gb/pdfs/stm201520160034000engpdfs.pdf2017.
22. Dziedzic K, Nicholls E, Hill S, Hammond A, Handy J, Thomas E, et al. Self-management approaches for osteoarthritis in the hand: a 2x2 factorial randomised trial. *Annals of the rheumatic diseases.* 2015;74(1):108-118:10.1136/annrheumdis-2013-203938
23. Ekman B, Nero H, Lohmander LS, Dahlberg LE. Costing analysis of a digital first-line treatment platform for patients with knee and hip osteoarthritis in Sweden. *PLoS One.* 2020;15(8):e0236342:10.1371/journal.pone.0236342
24. Tveter A, Blanck T, Nyheim S, Maarnes M, Christensen B, Pedersen S, et al. OP0122-HPR DEVELOPMENT OF A SMARTPHONE APPLICATION FOR TREATMENT OF HAND OSTEOARTHRITIS – HAPPY HANDS *Ann Rheum Dis.* 2022;81:79:DOI: 10.1136/annrheumdis-2022-eular.4343
25. Sanders GD, Neumann PJ, Basu A, Brock DW, Feeny D, Krahn M, et al. Recommendations for Conduct, Methodological Practices, and Reporting of Cost-effectiveness Analyses: Second Panel on Cost-Effectiveness in Health and Medicine. *JAMA.* 2016;316(10):1093-1103:10.1001/jama.2016.12195

For peer review only

For peer review only - http://bmjopen.bmj.com/site/about/guidelines.xhtml

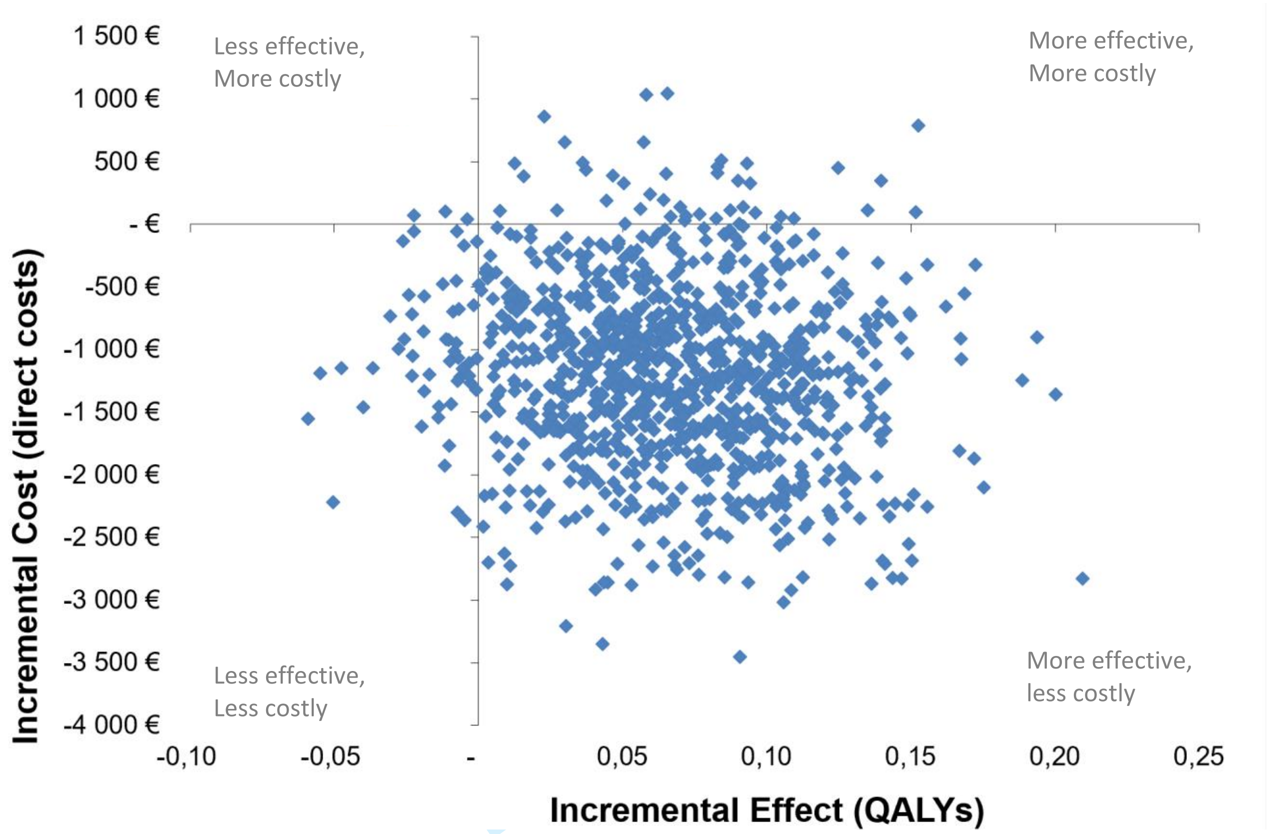

Figure 1

For peer review only

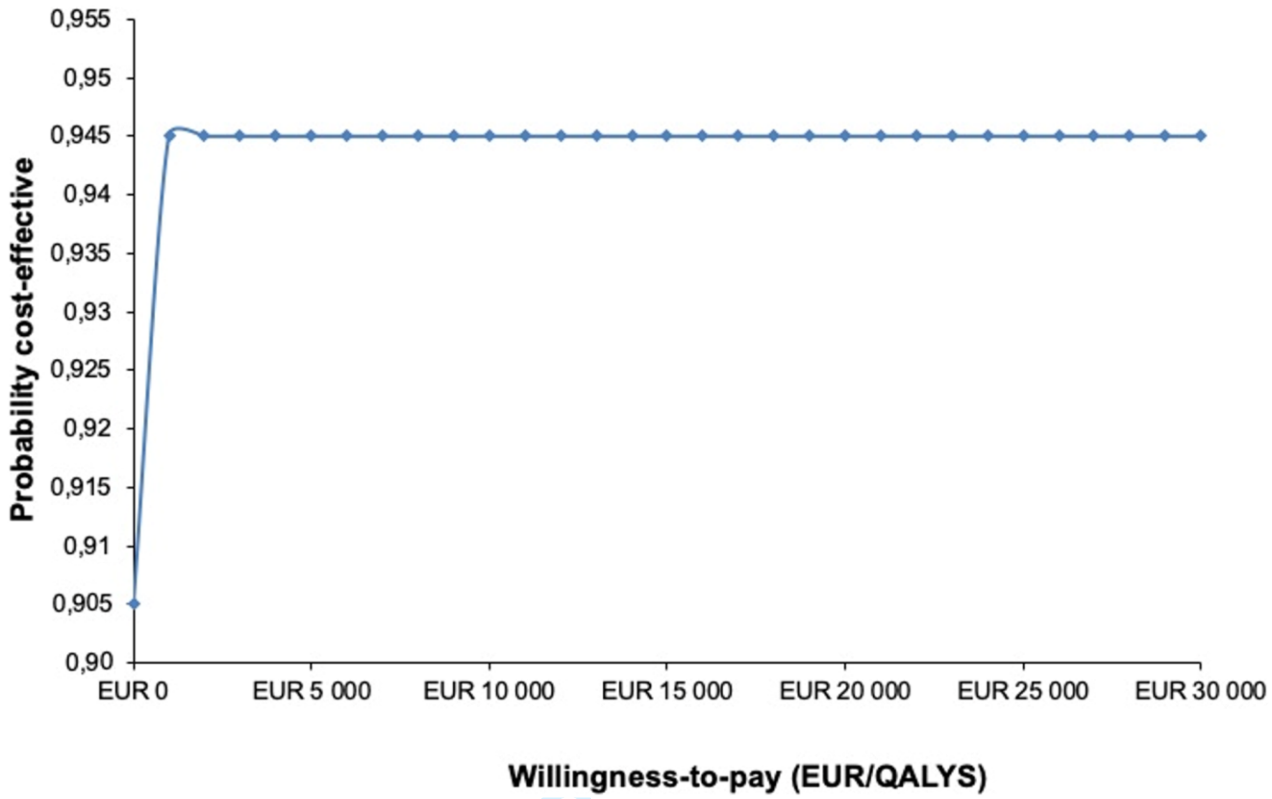

Figure 2

For peer review only

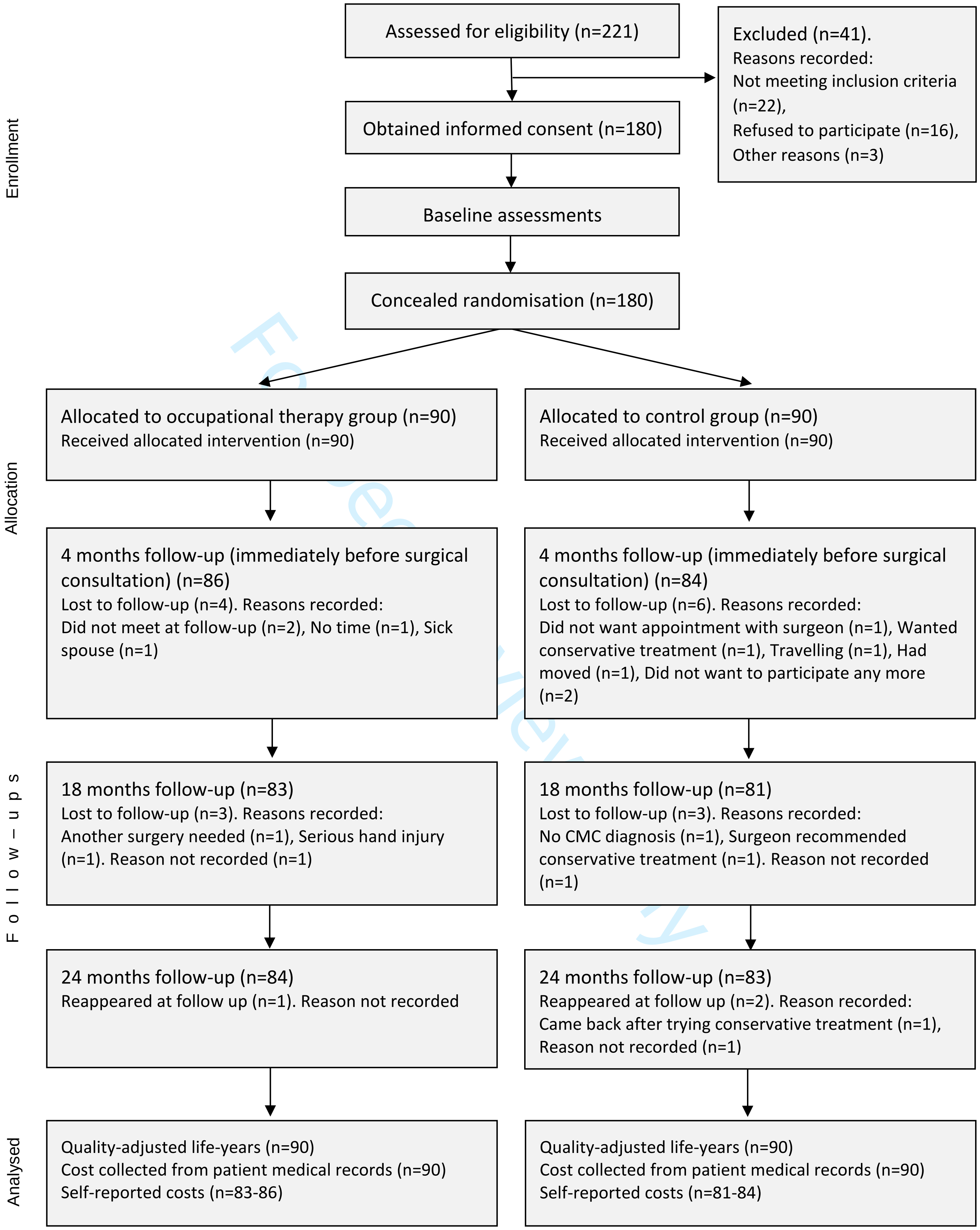

Supplementary figure A

For peer review only - http://bmjopen.bmj.com/site/about/guidelines.xhtml

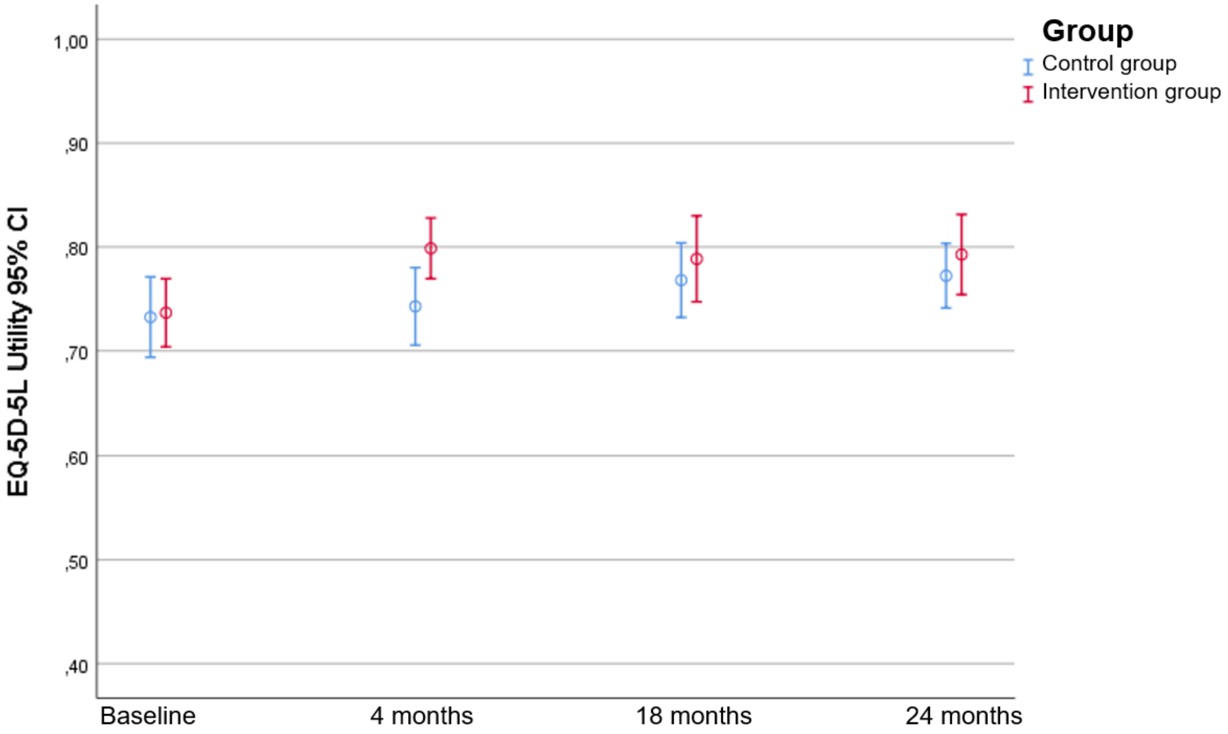

Supplementary figure B

For peer review only

CHEERS 2022 Checklist

**Title**			
Title	1	Identify the study as an economic evaluation and specify the interventions being compared.	page 1
**Abstract**			
Abstract	2	Provide a structured summary that highlights context, key methods, results, and alternative analyses.	pag 2
**Introduction**			
Background and objectives	3	Give the context for the study, the study question, and its practical relevance for decision making in policy or practice.	page 3-4
Methods			
**Health economic analysis plan**	**4**	**Indicate whether a health economic analysis plan was developed and where available.**	**page 3**
Study population	5	Describe characteristics of the study population (such as age range, demographics, socioeconomic, or clinical characteristics).	table 1
Setting and location	6	Provide relevant contextual information that may influence findings.	page 3-4
Comparators	7	Describe the interventions or strategies being compared and why chosen.	page 5
Perspective	8	State the perspective(s) adopted by the study and why chosen.	page 4
Time horizon	9	State the time horizon for the study and why appropriate.	page 4

For peer review only

(continued)

Discount rate	10	Report the discount rate(s) and reason chosen.	page 6-7
Selection of outcomes	11	Describe what outcomes were used as the measure(s) of benefit(s) and harm(s).	page 6-7
Measurement of outcomes	12	Describe how outcomes used to capture benefit(s) and harm(s) were measured.	page 6-7
Valuation of outcomes	13	Describe the population and methods used to measure and value outcomes.	page 4, page 6-7
Measurement and valuation of resources and costs	14	Describe how costs were valued.	page 7
Currency, price date, and conversion	15	Report the dates of the estimated resource quantities and unit costs, plus the currency and year of conversion.	table 2
Rationale and description of model	16	If modelling is used, describe in detail and why used. Report if the model is publicly available and where it can be accessed.	ua
Analytics and assumptions	17	Describe any methods for analysing or statistically transforming data, any extrapolation methods, and approaches for validating any model used.	page 7-8
Characterising heterogeneity	18	Describe any methods used for estimating how the results of the study vary for subgroups.	ua
Characterising distributional effects	19	Describe how impacts are distributed across different individuals or adjustments made to reflect priority populations.	ua

For peer review only - http://bmjopen.bmj.com/site/about/guidelines.xhtml

(continued)

Characterising uncertainty	20	Describe methods to characterise any sources of uncertainty in the analysis.	page 7
Approach to engagement with patients and others affected by the study	21	Describe any approaches to engage patients or service recipients, the general public, communities, or stakeholders (such as clinicians or payers) in the design of the study.	page 4
**Results**			
Study parameters	22	Report all analytic inputs (such as values, ranges, references) including uncertainty or distributional assumptions.	table 3, figure 1
Summary of main results	23	Report the mean values for the main categories of costs and outcomes of interest and summarise them in the most appropriate overall measure.	page 8-9, table 3
Effect of uncertainty	24	Describe how uncertainty about analytic judgments, inputs, or projections affect findings. Report the effect of choice of discount rate and time horizon, if applicable.	page 9-11
Effect of engagement with patients and others affected by the study	25	Report on any difference patient/service recipient, general public, community, or stakeholder involvement made to the approach or findings of the study	page 4
Discussion			

For peer review only - http://bmjopen.bmj.com/site/about/guidelines.xhtml

(continued)

Study findings, limitations, generalisability, and current knowledge	26	Report key findings, limitations, ethical or equity considerations not captured, and how these could affect patients, policy, or practice.	page 9-11
Other relevant information			
Source of funding	27	Describe how the study was funded and any role of the funder in the identification, design, conduct, and reporting of the analysis	page 13
Conflicts of interest	28	Report authors conflicts of interest according to journal or International Committee of Medical Journal Editors requirements.	page 13

From: Husereau D, Drummond M, Augustovski F, et al. Consolidated Health Economic Evaluation Reporting Standards 2022 (CHEERS 2022) Explanation and Elaboration: A Report of the ISPOR CHEERS II Good Practices Task Force. Value Health 2022;25. doi:10.1016/j.jval.2021.10.008

For peer review only

BMJ Open

Is multimodal occupational therapy in addition to usual care cost-effective in people with carpometacarpal osteoarthritis? A cost-utility analysis of a randomized controlled trial

Journal:	*BMJ Open*
Manuscript ID	bmjopen-2022-063103.R2
Article Type:	Original research
Date Submitted by the Author:	23-Mar-2023
Complete List of Authors:	TVETER, Anne Therese; Diakonhjemmet Hospital Norwegian National Advisory Unit on Rehabilitation in Rheumatology, Center for treatment in rheumatic and musculoskeletal diseases (REMEDY), Department of Rheumatology and Research<br>Kleven, Linn; Norwegian Institute of Public Health<br>Osteras, Nina; Diakonhjemmet Hospital Norwegian National Advisory Unit on Rehabilitation in Rheumatology, Center for treatment in rheumatic and musculoskeletal diseases (REMEDY), Department of Rheumatology and Research<br>Nossum, Randi; St Olavs Hospital Trondheim University Hospital<br>Eide, Ruth Else Mehl; Haukeland University Hospital<br>Klokkeide, Åse; Haugesund Sanitary Association Rheumatism Hospital<br>Matre, Karin Hoegh; Haukeland University Hospital<br>Olsen, Monika; Haugesund Sanitary Association Rheumatism Hospital<br>Kjeken, Ingvild; Diakonhjemmet Hospital Norwegian National Advisory Unit on Rehabilitation in Rheumatology, Center for treatment in rheumatic and musculoskeletal diseases (REMEDY), Department of Rheumatology and Research
**Primary Subject Heading**:	Health economics
Secondary Subject Heading:	Rehabilitation medicine, Rheumatology
Keywords:	HEALTH ECONOMICS, Musculoskeletal disorders < ORTHOPAEDIC & TRAUMA SURGERY, Hand & wrist < ORTHOPAEDIC & TRAUMA SURGERY, RHEUMATOLOGY

SCHOLARONE™
Manuscripts

I, the Submitting Author has the right to grant and does grant on behalf of all authors of the Work (as defined in the below author licence), an exclusive licence and/or a non-exclusive licence for contributions from authors who are: i) UK Crown employees; ii) where BMJ has agreed a CC-BY licence shall apply, and/or iii) in accordance with the terms applicable for US Federal Government officers or employees acting as part of their official duties; on a worldwide, perpetual, irrevocable, royalty-free basis to BMJ Publishing Group Ltd ("BMJ") its licensees and where the relevant Journal is co-owned by BMJ to the co-owners of the Journal, to publish the Work in this journal and any other BMJ products and to exploit all rights, as set out in our licence.

The Submitting Author accepts and understands that any supply made under these terms is made by BMJ to the Submitting Author unless you are acting as an employee on behalf of your employer or a postgraduate student of an affiliated institution which is paying any applicable article publishing charge ("APC") for Open Access articles. Where the Submitting Author wishes to make the Work available on an Open Access basis (and intends to pay the relevant APC), the terms of reuse of such Open Access shall be governed by a Creative Commons licence – details of these licences and which Creative Commons licence will apply to this Work are set out in our licence referred to above.

Other than as permitted in any relevant BMJ Author's Self Archiving Policies, I confirm this Work has not been accepted for publication elsewhere, is not being considered for publication elsewhere and does not duplicate material already published. I confirm all authors consent to publication of this Work and authorise the granting of this licence.

For peer review only

Is multimodal occupational therapy in addition to usual care cost-effective in people with carpometacarpal osteoarthritis? A cost-utility analysis of a randomized controlled trial

Anne Therese Tveter[1], Linn Kleven[2], Nina Østerås[1], Randi Nossum[3], Ruth Else Mehl Eide[4], Åse Klokkeide[5], Karin Hoegh Matre[4], Monika Olsen[5], Ingvild Kjeken[1]

[1] Norwegian National Advisory Unit on Rehabilitation in Rheumatology, Center for treatment in rheumatic and musculoskeletal diseases (REMEDY), Department of Rheumatology and Research, Diakonhjemmet Hospital, Oslo, Norway

[2] Norwegian Institute of Public Health, Oslo, Norway

[3] St.Olavs Hospital, Trondheim University Hospital, Trondheim, Norway

[4] Haukeland University Hospital, Bergen, Norway

[5] Haugesund Sanitary Association Rheumatism Hospital, Haugesund, Norway

Corresponding author:

Anne Therese Tveter

Center for treatment of rheumatic and musculoskeletal diseases (REMEDY)

Diakonhjemmet Hospital

Postboks 23 Vinderen, 0319 Oslo, Norway

a.t.tveter@medisin.uio.no, https://orcid.org/0000-0003-1701-9835

For peer review only - http://bmjopen.bmj.com/site/about/guidelines.xhtml

ABSTRACT

Objective: The aim was to evaluate the cost-utility of a 3-month multimodal occupational therapy intervention in addition to usual care in patients with carpometacarpal osteoarthritis (OA).

Methods: A cost-utility analysis was performed alongside a multicentre randomized controlled trial including three rheumatology departments in Norway. A total of 180 patients referred to surgical consultation due to carpometacarpal OA were randomized to either multimodal occupational therapy including patient education, hand exercises, assistive devices, and orthoses (n=90), or usual care receiving only information on OA (n=90). The outcome measure was quality-adjusted life-years (QALYs) derived from the generic questionnaire EQ-5D-5L over a 2-year period. Resource use and health-related quality of life of the patients were prospectively collected at baseline, 4, 18, and 24 months. Costs were estimated by taking a healthcare and societal perspective. The results were expressed as incremental cost-effectiveness ratios (ICERs), and a probabilistic sensitivity analysis with 1 000 replications following intention-to-treat principle was done to account for uncertainty in the analysis.

Results: During the 2-year follow-up period, patients receiving multimodal occupational therapy gained 0.06 more QALYs than patients receiving usual care. The mean (SD) direct costs were € 3227 (3546) in the intervention group and € 4378 (5487) in the usual care group, mean difference €-1151 (95% CI -2564, 262). The intervention was the dominant treatment with a probability of 94.5% of being cost-effective given the willingness-to-pay threshold of € 27 500.

Conclusions: The within-trial analysis demonstrated that the multimodal occupational therapy in addition to usual care was cost-effective at 2 years in patients with carpometacarpal osteoarthritis.

Trial registration number: ClinicalTrials.gov (NCT01794754)

For peer review only - http://bmjopen.bmj.com/site/about/guidelines.xhtml

Keywords: hand osteoarthritis, occupational therapy, cost-effectiveness, cost-utility, quality-adjusted life-years (QALYs)

Strength and limitations of the study

- A strength of the study is the involvement of a patient research partner throughout the conduct of the study, although it may be considered a limitation that only one partner was involved.
- Self-reported costs combined with several months between assessments may have biased the total costs of the study.
- We were unable to include medication costs, travel expenses or purchase of technical or medical equipment in the analyses due to imprecise reporting.

INTRODUCTION

Hand osteoarthritis (OA) is the most common joint disease,[1] with the subcategory carpometacarpal osteoarthritis (CMC1 OA) affecting the base of the thumb.[2] Approximately 22% of people aged ≥50 years have symptomatic CMC1 OA.[3] Compared to OA in the other finger joints, CMC1 OA is associated with more pain and functional limitations, negatively affecting work ability and quality of life.[2,4,5]

There is a common misconception that hand OA is a normal part of aging, and that there is 'nothing to be done'.[6] Although there is no cure for OA, the European League Against Rheumatism (EULAR) recommends that all people with hand OA should be offered a multimodal intervention consisting of information, hand exercises, and assistive devices as first-line treatment.[7] In addition, orthoses should be provided for patients with CMC1 OA.[7] Pharmacological therapy is recommended as a symptom relieving supplemental intervention, whereas surgery should only be considered when other treatments have failed to provide sufficient pain relief.[7] The multimodal intervention is shown to have a statistically significant beneficial short-term effect on pain[8,9] and hand function[9] compared to or in addition to usual care. However, there is an evidence-to-practice gap regarding this first-line multimodal intervention, with only 20% reporting to have received this treatment before being referred to surgical consultation.[10] This

For peer review only - http://bmjopen.bmj.com/site/about/guidelines.xhtml

intervention also presents a tendency towards reduction and delay in surgery rate over a 2-year period.[11] Although not significant, this tendency may still be of clinical importance if shown to be cost-effective.

Oppong et al performed a trial-based economic evaluation on joint protection only, hand exercises only and a combination of joint protection and hand exercises compared to leaflet and advice over a 12-month period in patients with hand OA.[12] They found hand exercises to be the most cost-effective alternative.[12] In the study by Adams however, providing orthoses only was found to be neither effective nor cost-effective.[13] Thus, the results are regarding the different components of recommended treatment are not conclusive. To the best of our knowledge, no studies have assessed the cost-utility of the different components combined in a multimodal intervention following the EULAR recommendations for the treatment of hand OA or CMC1 OA.

The aim of this study was to perform a cost-utility analysis of a 3-month multimodal occupational therapy intervention in addition to usual care in patients with CMC1 OA referred to surgical consultation. The outcome measure was quality adjusted life-years (QALYs), and the costs were estimated both as direct and indirect costs and societal costs over a 2-year period taking a healthcare and societal perspective.

METHODS

Study design and setting

A cost-utility analysis (CUA) was performed alongside a multicentre RCT including rheumatology departments in three Norwegian hospitals (St. Olav's Hospital in Trondheim, Haukeland University Hospital in Bergen, and Haugesund Rheumatism Hospital) and is described in the protocol article.[11,14] In Norway, most hospitals (including the three hospitals involved in this trial) are public and owned by the Norwegian government. The trial was approved by the Norwegian Regional Ethical Committee (2012/2265/REK sør-øst C).

Patient and Public Involvement

For peer review only - http://bmjopen.bmj.com/site/about/guidelines.xhtml

For peer review only

A patient research partner from the Patient Research Panel at Diakonhjemmet Hospital, who had lived with hand osteoarthritis for several years, was involved throughout the project. She participated in project meetings where she gave input to the project plan, recruitment procedure, and information material as well as feedback on the interpretation and dissemination of the results.

Participants and randomization

Patients referred by their general practitioner for surgical consultation due to CMC1 OA at the three rheumatology departments between 2013 and 2015 were considered eligible for the study if they could speak Norwegian and had no cognitive deficits. Eligible patients received information about the study, a written consent form and a questionnaire by post. Patients who agreed to participate were scheduled for an appointment with an occupational therapist (OT) at the hospital within 2 weeks after referral. After a baseline assessment, the patients were randomized to either a 3-month multimodal occupational therapy intervention or usual care. In Norway, the waiting period between referral and the actual surgical consultation was at the time this study was conducted between 4 and 6 months, and the intervention was conducted during this waiting period. Patients were assessed again immediately before surgical consultation (approximately 4 months after baseline assessment), and 18 and 24 months after the baseline assessment (Supplementary figure A). The sample size of 180 participants was estimated based on an expected surgery rate of 70% during the 2-year follow-up, which was the primary endpoint of the RCT.[11,14]

Intervention

During the waiting period between referral and surgical consultation, the intervention group received a multimodal occupational therapy intervention in line with recommended first-line treatment.[7] In short, at the baseline assessment, the patients received written and oral information on hand OA, ergonomic principals, and the use of assistive devices by an OT. They were instructed in a hand exercise program to be performed at home three times per week for 12 weeks. At no cost, they all received five common assistive devices (a bread knife and vegetable knife with built-up handles, an enlarged grip for

For peer review only - http://bmjopen.bmj.com/site/about/guidelines.xhtml

opening bottles, a key for opening jars [the Jar Key], and a self-opening pair of scissors) for use when needed, and daytime and nighttime orthoses that they were encouraged to use as much as possible.[14] The patients in the intervention group consulted the OT twice during this intervention period: at the baseline assessment and again after 2 weeks to adjust the orthoses and exercise program, implying that the intervention was based mostly on self-management. Patients referred for bilateral hand OA received treatment for both hands. At the follow-up assessments (4, 18, and 24 months), the patients in the intervention group could receive new orthoses, or the OTs could adjust the ones they already had.

The control group received usual care, which generally means staying on the waiting list for consultation in specialist healthcare, i.e., receiving no treatment in specialist healthcare. For the purpose of this study, the control group were assessed at the hospital at baseline and all follow-up time points by an OT. They received general written and oral information about hand OA. The control group had the same possibility as the intervention group to ask questions and be given advice.

If necessary, patients in both groups could independently seeking treatment in primary healthcare, such as a consultation with general practitioner or other healthcare personnel (this information was collected as part of this study). Both groups had a surgical consultation with a medical specialist approximately 4 months after the baseline assessment. All patients paid a deductible for the assessment consultations (a maximum of €259 per year).

Randomization and blinding

Patients were randomized using a computer-generated list with a block size of 10, stratified by hospital. Envelopes were opened by the patients after receiving information on hand OA and completing baseline assessment. The group affiliation was known to both the patients and the OTs.

Data collection

Health outcome

The health-related quality of life (HRQoL) was measured at baseline, 4, 18, and 24 months using the generic instrument EQ-5D-5L (EuroQol 5-dimension, www.euroqol.org).[15] The EQ-5D-5L is a preference

For peer review only

weighted measure of HRQoL based on five dimensions: mobility, self-care, daily activities, pain/discomfort, and anxiety/depression. For each dimensions the patient assesses five possible levels of problems, from no problems to severe.[15] Quality-adjusted life-years (QALYs) were derived from the EQ-5D-5L utility score, using the preference score from a UK population. Since there is no Norwegian tariff, the recommendation in Norway is to use the British tariff.[16] QALYs are weighted for time (years of life) with an index that express the HRQoL (utilities) on a scale anchored at 0 (health state equivalent to death) and 1 QALY (full health).

Resource use and costs

Healthcare utilization was collected from different sources at baseline, 4, 18 and 24 months. The OT reported the number of consultations related to the intervention at each assessment, whereas surgical procedures and post-operative treatment were collected from patients' medical record. In addition, the use of primary and specialist healthcare was self-reported by the patient. We have included the costs related to the OT assessment at baseline, 4, and 18 months for both groups (OT assessment at 24 months were not included as the usual care group then received the intervention). Although the OT assessment was done for study purposes, it was included in the cost estimates because we cannot rule out a possible positive effect of an appointment with an OT in specialist healthcare for the control group. We have not included medication costs, travelling expenses or purchase of technical or medical equipment (except assistive devices provided to patients in the intervention group) due to lack of or imprecise reporting. Many patients reported using medication when needed, making it impossible to calculate costs. Travelling expenses were not collected, and with regard to technical or medical equipment, it was not possible to determine if the patients had bought the equipment before or during the trial.

Data on sickness absence related to surgery were collected from patient medical records. The number of hours of informal help at home due to OA (from spouse, family, or friends) and the productivity loss (due to any reason of sickness absence and disability benefits, excluding sickness absence related to surgery) were self-reported. If not explicitly indicated by the patient, we expected that the productivity loss remained the same between assessment points, thus, if the patient reported sickness absence of 50% at 4

For peer review only - http://bmjopen.bmj.com/site/about/guidelines.xhtml

months and 100% at 18 months, this was calculated as 50% productivity loss between 4 and 18 months, and 100% between 18 and 24 months.

The costs of specialist healthcare visits and surgery were calculated using diagnosis-related group (DRG) weights from 2019. DRG is a coding system used for administration of both clinical and financial activity in specialist healthcare. The DRG costs were estimated by multiplying the cost weighting for the specific DRG group of the patient with the unit cost of 2019.[17] Sickness absence and disability benefits were valued in the same way for all patients. Costs related to absence from work were estimated from official statistics on the average wage by sex and age group in 2019. The average daily productivity loss was valued as € 336 including societal costs of 40% and was adjusted for the percentage of sickness absence/disability benefits.[18] The number of hours of informal help at home was valued as a minimum wage of € 18 per hour.[19] All costs are estimated in Euros, using the exchange rate 0.10 (€ 1 = NOK 10) from 2019.

Direct costs included costs related to specialist healthcare (medical specialist, occupational therapist, physical therapist, nurse, radiographs, surgery, assistive devices) and primary healthcare (general practitioner, physical therapist, manual therapist, acupuncturist, blood samples). Indirect costs included productivity loss due to sickness absence (due to surgery or for other reasons) and costs related to help with chores at home.

Statistics

Differences in QALYs between the two groups were estimated using the trapezoidal method (area under the curve).[20] To illustrate the statistical uncertainty surrounding the incremental cost-effectiveness ratio (ICER), a probabilistic sensitivity analysis with 1 000 replications was performed using the bootstrap resampling method. The bootstrapped costs and effects can be illustrated on a cost-effectiveness plane (CE plane). The CE plane (Figure 1) are divided into four quadrants; where a new treatment that is more effective and less costly is plotted in the southeast quadrant, while one more costly and less effective is plotted in the northwest quadrant. Figure 2 shows the cost-effectiveness acceptability curve (CEAC), which presents the probability that the intervention is cost-effective in addition to usual care for hand OA. The willingness-to-pay threshold was set to € 27 500 (275 000 NOK) due to the low severity of the condition.[21]

For peer review only - http://bmjopen.bmj.com/site/about/guidelines.xhtml

Between-group differences in mean costs and mean effects were analyzed using Independent sample t-test. Significance level was set to 0.05. Statistical analyses were performed using Excel, SPSS version 26 and Stata version 16.

Missing data

A total of 18 patients (7 in the intervention group and 11 in the control group) had missing HRQoL utility score at one or more timepoints. For these missing values we used 'last observation carried forward'. Sensitivity analyses showed that the average HRQoL utility scores remained the same for both groups without imputation of these missing values. Missing self-reported costs were not imputed (n=16). The costs related to surgery were collected from patient medical records and were not encumbered by missing data.

RESULTS

Baseline characteristics

A total of 180 patients were randomized to either multimodal occupational therapy (n=90) or usual care (n=90). The mean age across both groups was 63 years (SD 7.6) and 79% of the patients were women. We found no between-group differences in any of the baseline characteristics including utility score, medication use, or percentage of patients working full- or part-time (Table 1).

For peer review only - http://bmjopen.bmj.com/site/about/guidelines.xhtml

Table 1 Demographic variables for patients referred to surgical consultation due to carpometacarpal osteoarthritis (n = 180). All information was collected at the baseline assessment

		Intervention group (n=90)	Control group (n=90)
Age, years, mean (SD)		62.8 (7.5)	63.3 (7.8)
Female sex, *n* (%)		73 (81)	69 (77)
Referred hand, *n* (%)			
	Right	31 (34)	17 (19)
	Left	24 (27)	29 (32)
	Both	35 (39)	44 (49)
Education level, *n* (%)			
	Primary school	14 (16)	20 (22)
	High school	44 (49)	39 (43)
	University/college ≤ 4 y	17 (19)	19 (21)
	University/college > 4 y	15 (16)	12 (13)
Living alone, *n* (%)		17 (19)	18 (20)
Working full/part-time, *n* (%)		48 (53)	43 (48)
Years with symptoms, median (IQR)		5 (2–10)	5 (2–10)
Presence of radiographic subluxation of the CMC joint		58 (64)	55 (61)
Comorbidity‡, *n* (%)		61 (68)	56 (62)
Analgesics use (anti-inflammatory or pain relief medication), *n* (%)		57 (63)	57 (63)
Pain at rest (NRS 0–10, 0 = no pain), median (IQR)		3 (1–4)	3 (2–5)
EQ-5D-5L utility score (1=best health), mean (SD)		0.74 (0.16)	0.73 (0.18)

SD= standard deviation; IQR= interquartile range (25th and 75th percentile); CMC= carpometacarpal joint; NRS= numeric rating scale; Pain at rest was reported for referred hand (the mean of both hands if bilateral referral); ‡ Comorbidities was categorized as present if they had any of 16 predefined comorbidities[14].

Cost-utility analysis

The total between-group difference in QALYs was 0.06 (95% CI -0.02, 0.15) after 24 months, in favor of the intervention group. The distribution of EQ-5D-5L utility score across the different time points for the two groups are shown in Supplementary figure B. The mean healthcare costs and productivity loss for both treatment groups are presented in Table 2.

For peer review only - http://bmjopen.bmj.com/site/about/guidelines.xhtml

Table 2 Mean direct and indirect healthcare costs (mean and standard deviation) per patient for the intervention and control group (n=180). The table also show unit price and the resource use (n).

		Unit	Unit price, €	n	Intervention group Mean (SD) per patient, €	n	Control group Mean (SD) per patient, €	Mean difference (95% CI), €
**Direct costs**	*Specialist healthcare*							
	Medical specialist	Per visit	205[a]	136	309 (268)	169	384 (365)	-75 (-169, 19)
	Occupational therapist including adaptation of orthoses	Per visit	246[a]	161	536 (318)	65	158 (367)	377 (275, 478)
	Occupational / Physical therapist	Per visit	205[a]	169	428 (350)	152	837 (866)	-355 (-549, -160)
	Nurse	Per visit	205[a]	1	16 (98)	9	48 (189)	-32 (-76, 13)
	Inpatient surgery	Per surgery	8 435[a]	2	187 (1 250)	6	562 (2 465)	-375 (-951, 201)
	Day-bed surgery	Per surgery	2 251[a]	20	500 (941)	27	675 (1 190)	-175 (-491, 141)
	Radiographs	Per visit	149 [e]	90	149 (0)	90	149 (0)	-
	Assistive devices	Per person	67 [e]	90	67 (0)	0	-	-
	Primary healthcare							
	General practitioner	Per visit	32[b]	91	32 (94)	130	46 (104)	-14 (-43, 15)
	Physical therapist	Per visit	31[c]	187	64 (288)	158	54 (160)	10 (-59, 79)
	Manual therapist	Per visit	52[c]	0	-	16	10 (90)	-
	Acupuncturist	Per visit	47[e]	22	11 (64)	44	23 (132)	-11 (-42, 19)
	Blood samples	Per test	7[b]	14	1 (6)	24	2 (6)	-1 (-2, 1)
**Indirect costs**								
	Sickness absence due to surgery	Days	336[d]	65	1 212 (3 815)	64	1 199 (3 817)	186 (-1 104, 1 141)
	Costs related to help with chores at home	Hours	18[d]	3261	652 (1 722)	4612	922 (3 089)	-270 (-1 006, 465)
	Sickness absence and disability benefits (not sickness absence due to surgery)	Days	336[d]	10351	38 299 (63 029)	12 249	45 433 (66 320)	-7 133 (-26 167, 1 190)

[a] DRG (diagnosis-related group system); [b] List of reimbursement codes; [c] Rates for physiotherapists; [d] Official statistics of average wage; [e] Based on real costs. All costs are given in Euro (€1=NOK10) and adjusted to 2019.

For peer review only - http://bmjopen.bmj.com/site/about/guidelines.xhtml

The mean cost of multimodal occupational therapy intervention (baseline information on hand OA, instruction in hand exercises, assistive devices, and customization of orthoses, and adjustment of hand exercises and orthoses after 14 days) was approximately € 500 per patient. Surgery accounted for the largest between-group difference with 22 surgical procedures in the intervention group and 33 surgical procedures in the control group, resulting in a €550 difference in surgical cost per patient between the two groups. The use of physiotherapy/occupational therapy also constituted a high cost. However, almost one-third of these costs were related to the predetermined assessment points (4, 18 and 24 months) that were part of the study but not the actual OT intervention (€375 per patient in the control group and €307 per patient in the intervention group).

Productivity loss due to sickness absence or disability benefits was the largest contributor to the total costs in both groups. However, at baseline, only 7% of the sickness absence in both groups was reported to be due to hand OA, and the percentages at 4, 18, and 24 months were 6%, 2% and 0.5%, respectively. Similar numbers of patients in the two groups were retired at baseline (34% in the control group versus 32% in the intervention group), 4 months (36% versus 34%), and 24 months (42% versus 43%). However, more patients in the intervention group (43%) retired between 4 and 18 months than in the control group (36%), indicating that fewer patients contributed to productivity loss in the intervention group during this period.

Table 3 shows the mean incremental costs and incremental effects associated with direct and indirect healthcare costs. In the case of a negative ICER, the evaluated treatment is either dominant (more effective and less costly) or dominated (less effective and more costly). The intervention in this study was the dominant treatment (Figure 1) with a 94.5% probability of being cost-effective given the willingness-to-pay threshold (Figure 2).

For peer review only - http://bmjopen.bmj.com/site/about/guidelines.xhtml

Table 3 Cost-utility analysis of mean costs (SD), mean effects (SD), incremental differences in costs and effects (95% CI), and incremental cost-effectiveness ratio (ICER) for direct and total direct and indirect costs (n=180)

	Intervention group (n=90)		Control group (n=90)				
	**Mean cost (SD)**	**Mean effect* (SD)**	**Mean cost (SD)**	**Mean effect* (SD)**	**Incremental cost (95% CI)**	**Incremental effect (95% CI)**	**ICER**
Direct costs	3 227 (3 546)	1.58 (0.28)	4 378 (5 487)	1.51 (0.28)	-1 151 (-2 564, 262)	0.06 (-0.02, 0.15)	Dominant
Total costs (direct and indirect costs)	46 617 (65 499)	1.58 (0.28)	56 304 (68 872)	1.51 (0.28)	-9 688 (-9 949, 29 324)	0.06 (-0.02, 0.15)	Dominant

Direct costs, **€**: self-reported use of primary and specialist healthcare, including surgery;

Indirect costs, **€**: productivity loss from sickness absence due to surgery, costs related to informal help at home, productivity loss from sickness absence and disability benefits excluding sickness absence due to surgery

Total cost, **€**: direct costs + indirect costs

* Mean effect without imputations; Intervention group 1.58 (0.28), Control group 1.51 (0.28)

SD= standard deviation

For peer review only

For peer review only - http://bmjopen.bmj.com/site/about/guidelines.xhtml

DISCUSSION

The aim of this study was to evaluate the cost-utility of a 3-month multimodal occupational therapy intervention in patients with CMC1 OA in a 2-year period. Our results demonstrate that the intervention, consisting of patient education, hand exercises, orthoses, and assistive devices, has the highest probability of being cost-effective, given the willingness-to-pay threshold (€ 27 500/QALY), indicating that the intervention is both less costly and more effective in addition to usual care. This study is one of few studies to evaluate the cost-utility of conservative treatment in hand OA, and the only study to evaluate the cost-utility of the 2018 EULAR guidelines on first-line treatment for CMC1 OA. Our findings add to the evidence base by demonstrating that multimodal occupational therapy is a cost-effective alternative for the management of CMC1 OA.

The results of this study are in line with a previous study showing that hand exercises were more cost-effective than leaflet and advice in patients with hand OA.[12] In contrast, the study by Oppong et al. failed to show that the combination of hand exercises and use of assistive devices (joint protection classes[22]) were more cost-effective than hand exercises alone.[12] Similarly, the use of orthoses alone was shown to be neither effective nor cost-effective.[13] The results from the current study should, however, be replicated in new studies taking into account also travelling cost, medication, and technical and medical equipment.

In our study, we did not separately evaluate the different parts of the recommended first-line treatment. It is possible that we would have found the same results as Oppong et al.,[12] given that hand exercises only entail costs related to the time used by health personnel to educate the patient, whereas both orthoses and assistive devices require an additional cost. On the other hand, the combination of the recommended first-line treatment was found to have a significant 3-month short-term effect on reducing pain and improving hand function in these patients,[8] and should be offered to all patients with CMC1 OA as an effective and cost-effective alternative. Due to the self-managing nature of the intervention, the mean cost related to the multimodal occupational therapy intervention was low, approximately €500 per patient. However, the current study was conducted in specialist healthcare, and there are other options for

For peer review only - http://bmjopen.bmj.com/site/about/guidelines.xhtml

delivering this intervention that may be even more cost-effective. The treatment of patients with OA should mainly be conducted in primary healthcare, possibly reducing the costs to some extent. However, due to the lack of availability of treatment options and long travel distances in parts of the country, more cost-effective options for delivery of treatment could be further explored, e.g. using a smart phone application as shown in a Swedish study on digital first-line treatment for hip and knee OA.[23] We are currently evaluating the effect and cost-utility of a newly developed smartphone app (the Happy Hands app) which is designed to make recommended treatment available to people with hand OA.[24]

Surgery constituted a large cost in this within-trial. Although we found a tendency towards reduction in surgical procedures in the intervention group compared to the control group, this result was not statistically significant.[11] Still, the between-group difference in mean surgical costs (€ 550) was higher than the mean cost of the multimodal occupational therapy intervention alone (€ 500). Thus, although the difference in surgical rates between the two groups were found to be non-significant,[11] the difference may still be regarded as important in a healthcare and societal perspective as surgical procedures may be prone to more adverse events compared to multimodal occupational therapy.[11] Most likely, the avoidance of surgery has the highest impact on the cost-effectiveness of the intervention.

The patients reported to have OA complaints for a median of 5 years, and more than 60% showed radiographic signs of subluxation of the carpometacarpal joint, indicating advanced disease progression. However, only 21% of the patients had visited a physiotherapist or an OT before referral to surgical consultation.[10] Thus, referral to a multimodal occupational therapy intervention at an earlier stage in the disease course may be a possible step towards reducing the costs related to healthcare use.

The current study is the first study assessing the cost-utility of recommended multimodal treatment in patients with CMC1 OA. It is considered a strength that the study was conducted alongside a well-designed RCT. The study also has some limitations. Almost 80% of the sample were women, which represents a higher proportion of women than the usual gender distribution in hand OA. The results may therefore not be representative for men with hand OA. The self-reported costs and the 14-month time frame between the reporting may have biased the total costs. This has most likely contributed to lower

For peer review only - http://bmjopen.bmj.com/site/about/guidelines.xhtml

overall costs, as patients may not remember to report all consultations with healthcare personnel, especially in the time period between 4 and 18 months. On the other hand, the costs related to surgery were collected from patient medical records and will not be encumbered by the same bias. A total of 16 patients had missing data throughout the study. There may have been systematic reasons for these patients being missing, which could have affected the outcome. However, there were more missing data in the control group than the intervention group, indicating that the between-group difference could have been larger if all patients had reported direct and indirect costs. The results may have been biased by the method of imputation of missing HRQoL utility values. However, due to low number of missing values and sensitivity analyses showing no difference in mean score whether using 'Last observation carried forward' or not, we did not go any further with more advanced methods like multiple imputation. We were unable to include medication costs, travel expenses or purchase of technical or medical equipment, except for the assistive devices provided as part on the intervention, in the analyses. This is not in line with the most recent recommendations for cost-utility analyses[25] and may have biased the results by possibly underestimating the total costs. However, the equal distribution of medication use across the two groups imply that this do not constitute a large bias in the comparison of results. As with all conservative treatments, the patients knew that they received the intervention, and this may be a potential bias improving the effect measure. Measurement properties of the EQ-5D-5L has not been assessed in patients with CMC OA. The HRQoL measure might also capture QoL related to other conditions than only CMC OA. Although the follow-up sessions were similar for both groups, the results may have been influenced by the fact that patients in the intervention group could receive adjustment of orthoses or new orthoses at these sessions. This may have influenced the results. The involvement of a patient research partner with lived experience on hand osteoarthritis is a strength, however, the study would have benefited from input from more than one partner. Future studies should evaluate the cost-utility of an even longer follow-up time as there is a lack of knowledge on surgical rate of these patients in the long term.

CONCLUSION

For peer review only

For peer review only - http://bmjopen.bmj.com/site/about/guidelines.xhtml

The within-trial analysis demonstrated that multimodal occupational therapy was cost-effective in addition to usual care in patients with CMC1 OA, when taking a healthcare and societal perspective. The intervention was the dominant treatment with a 94.5% probability of being cost-effective at 2 years, given the willingness-to-pay threshold of € 27 500/QALY. The low cost related to the intervention supports the use of this first-line treatment in all patients with CMC1 OA.

Acknowledgements

We would like to acknowledge patient research partner Øyvor Andreassen for her contribution throughout the project. We would also like to thank professor Tore K. Kvien for reading and commenting on the manuscript. The project has been acknowledged at the EULAR Congress with the HPR Abstract Award in 2019 for the primary outcome results and the HPR Abstract Award in 2020 for the results presented in this article.

Author contribution

Ingvild Kjeken was responsible for obtaining funding. Randi Nossum, Ruth Else Mehl Eide, Åse Klokkeide, Karin Hoegh Matre, and Monika Olsen contributed to data acquisition. Anne Therese Tveter, Linn Kleven, Nina Østerås, and Ingvild Kjeken were responsible for data analysis and interpretation. Further, all authors have made substantial contributions to the study conception and design, drafting of the article, critical revision of the article for important intellectual content and final approval of the submitted version.

Funding statement

This work was supported by the South-Eastern Norway Regional Health Authority (2015109) and Norges Forskningsråd (328657). The funding authorities had otherwise no involvement in the study. This research received no specific grant from any funding agency in the public, commercial or not-for-profit sector.

Conflict of interest

None of the authors have any financial or personal conflicts of interest.

For peer review only - http://bmjopen.bmj.com/site/about/guidelines.xhtml

Data sharing statement

Data are available upon reasonable request to the project manager (Ingvild Kjeken, Ingvild. kjeken@diakonsyk.no)

Ethics Statement

The trial was approved by the Norwegian Regional Ethical Committee (2012/2265/REK sør-øst C). All participants gave a written informed consent before taking part in the study.

For peer review only

For peer review only - http://bmjopen.bmj.com/site/about/guidelines.xhtml

Figure legends

Supplementary figure A Study flow diagram of a 2-year study period in patients referred to surgical consultation due to carpometacarpal osteoarthritis.

Supplementary figure B EQ-5D-5L utility score across the different timepoints, divided by group.

Figure 1 Cost-effectiveness (CE) plane of the ICERs of multimodal occupational therapy (intervention group) in addition to usual care (control group) were recalculated with 1 000 replications of the study data using the bootstrap resampling method. The intervention is at the origin.

Figure 2 shows a cost-effectiveness acceptability (CEA) curve for direct costs, representing the probability that the multimodal intervention is cost-effective in addition to usual care. At a willingness-to-pay threshold of € 27 500/QALY, the probability of multimodal intervention being cost-effective was 94.5%.

For peer review only - http://bmjopen.bmj.com/site/about/guidelines.xhtml

For peer review only

References

1. Marshall M, Watt FE, Vincent TL, Dziedzic K. Hand osteoarthritis: clinical phenotypes, molecular mechanisms and disease management. *Nature Reviews Rheumatology.* 2018;14(11):641-656:10.1038/s41584-018-0095-4
2. Bijsterbosch J, Visser W, Kroon HM, Stamm T, Meulenbelt I, Huizinga TW, et al. Thumb base involvement in symptomatic hand osteoarthritis is associated with more pain and functional disability. *Annals of the rheumatic diseases.* 2010;69(3):585-587:10.1136/ard.2009.104562
3. Marshall M, Peat G, Nicholls E, van der Windt D, Myers H, Dziedzic K. Subsets of symptomatic hand osteoarthritis in community-dwelling older adults in the United Kingdom: prevalence, inter-relationships, risk factor profiles and clinical characteristics at baseline and 3-years. *Osteoarthritis and cartilage.* 2013;21(11):1674-1684:10.1016/j.joca.2013.08.004
4. Spacek E, Poiraudeau S, Fayad F, Lefevre-Colau MM, Beaudreuil J, Rannou F, et al. Disability induced by hand osteoarthritis: are patients with more symptoms at digits 2-5 interphalangeal joints different from those with more symptoms at the base of the thumb? *Osteoarthritis and cartilage.* 2004;12(5):366-373:10.1016/j.joca.2004.01.005
5. Kwok WY, Vliet Vlieland TP, Rosendaal FR, Huizinga TW, Kloppenburg M. Limitations in daily activities are the major determinant of reduced health-related quality of life in patients with hand osteoarthritis. *Annals of the rheumatic diseases.* 2011;70(2):334-336:https://doi.org/10.1136/ard.2010.133603
6. Hill S, Dziedzic KS, Ong BN. The functional and psychological impact of hand osteoarthritis. *Chronic Illn.* 2010;6(2):101-110:10.1177/1742395309345614
7. Kloppenburg M, Kroon FP, Blanco FJ, Doherty M, Dziedzic KS, Greibrokk E, et al. 2018 update of the EULAR recommendations for the management of hand osteoarthritis. *Ann Rheum Dis.* 2018:10.1136/annrheumdis-2018-213826
8. Tveter AT, Østerås N, Nossum R, Eide REM, Klokkeide Å, Hoegh Matre K, et al. Short-term effects of occupational therapy on hand function and pain in patients with carpometacarpal osteoarthritis: secondary analyses from a randomized controlled trial. *Arthritis care & research.* 2020:10.1002/acr.24543
9. Deveza LA, Robbins SR, Duong V, Bennell KL, Vicenzino B, Hodges PW, et al. Efficacy of a Combination of Conservative Therapies vs an Education Comparator on Clinical Outcomes in Thumb Base Osteoarthritis: A Randomized Clinical Trial. *JAMA Intern Med.* 2021;181(4):429-438:10.1001/jamainternmed.2020.7101
10. Gravas EMH, Tveter AT, Nossum R, Eide REM, Klokkeide A, Matre KH, et al. Non-pharmacological treatment gap preceding surgical consultation in thumb carpometacarpal osteoarthritis - a cross-sectional study. *BMC musculoskeletal disorders.* 2019;20(1):180:10.1186/s12891-019-2567-3
11. Gravås EMH, Østerås N, Nossum R, Eide REM, Klokkeide Å, Matre KH, et al. Does occupational therapy delay or reduce the proportion of patients that receives thumb carpometacarpal joint surgery? A multicentre randomised controlled trial. *RMD Open.* 2019;5(2):e001046:10.1136/rmdopen-2019-001046
12. Oppong R, Jowett S, Nicholls E, Whitehurst DG, Hill S, Hammond A, et al. Joint protection and hand exercises for hand osteoarthritis: an economic evaluation comparing methods for the analysis of factorial trials. *Rheumatology (Oxford, England).* 2015;54(5):876-883:10.1093/rheumatology/keu389
13. Adams J, Barratt P, Rombach I, Arden N, Barbosa Bouças S, Bradley S, et al. The clinical and cost effectiveness of splints for thumb base osteoarthritis: a randomized controlled clinical trial. *Rheumatology (Oxford, England).* 2021;60(6):2862-2877:10.1093/rheumatology/keaa726

For peer review only - http://bmjopen.bmj.com/site/about/guidelines.xhtml

14. Kjeken I, Eide RE, Klokkeide A, Matre KH, Olsen M, Mowinckel P, et al. Does occupational therapy reduce the need for surgery in carpometacarpal osteoarthritis? Protocol for a randomized controlled trial. *BMC musculoskeletal disorders.* 2016;17(1):473:10.1186/s12891-016-1321-3
15. Herdman M, Gudex C, Lloyd A, Janssen M, Kind P, Parkin D, et al. Development and preliminary testing of the new five-level version of EQ-5D (EQ-5D-5L). *Qual Life Res.* 2011;20(10):1727-1736:10.1007/s11136-011-9903-x
16. Statens legemiddelverk. Guidelines for the submission of documentation for single technology assessment (STA) of pharmaceuticals. https://legemiddelverket.no/Documents/English/Public%20funding%20and%20pricing/Documentation%20for%20STA/Guidelines%2020.05.2020.pdf. Published 2018. Accessed 8.6.21, 2021.
17. Helsedirektoratet. [DRG-systemet]. https://www.helsedirektoratet.no/tema/finansiering/innsatsstyrt-finansiering-og-drg-systemet/drg-systemet#omdrgsystemet%E2%80%8B. Accessed 8.6.21, 2021.
18. Statistics Norway. [*Salary*]. https://www.ssb.no/arbeid-og-lonn/statistikker/lonnansatt. Accessed 20.08.2020.
19. Arbeidstilsynet. [*Minimum wage*]. https://www.arbeidstilsynet.no/arbeidsforhold/lonn/minstelonn/. Accessed 20.08.2020.
20. Drummond MF, Sculpher MJ, Claxton K, Stoddart GL, Torrance GW. *Methods for the Economic Evaluation of Health Care Programmes.* Fourth edition ed. Oxford: Oxford University Press; 2015.
21. Norwegian Ministry of Health and Care Services. Principles for priority setting in health care. Summary of a white paper on priority setting in the Norwegian health care sector. In. https://www.regjeringen.no/contentassets/439a420e01914a18b21f351143ccc6af/en-gb/pdfs/stm201520160034000engpdfs.pdf2017.
22. Dziedzic K, Nicholls E, Hill S, Hammond A, Handy J, Thomas E, et al. Self-management approaches for osteoarthritis in the hand: a 2x2 factorial randomised trial. *Annals of the rheumatic diseases.* 2015;74(1):108-118:10.1136/annrheumdis-2013-203938
23. Ekman B, Nero H, Lohmander LS, Dahlberg LE. Costing analysis of a digital first-line treatment platform for patients with knee and hip osteoarthritis in Sweden. *PLoS One.* 2020;15(8):e0236342:10.1371/journal.pone.0236342
24. Tveter A, Blanck T, Nyheim S, Maarnes M, Christensen B, Pedersen S, et al. OP0122-HPR DEVELOPMENT OF A SMARTPHONE APPLICATION FOR TREATMENT OF HAND OSTEOARTHRITIS – HAPPY HANDS *Ann Rheum Dis.* 2022;81:79:DOI: 10.1136/annrheumdis-2022-eular.4343
25. Sanders GD, Neumann PJ, Basu A, Brock DW, Feeny D, Krahn M, et al. Recommendations for Conduct, Methodological Practices, and Reporting of Cost-effectiveness Analyses: Second Panel on Cost-Effectiveness in Health and Medicine. *JAMA.* 2016;316(10):1093-1103:10.1001/jama.2016.12195

For peer review only

For peer review only - http://bmjopen.bmj.com/site/about/guidelines.xhtml

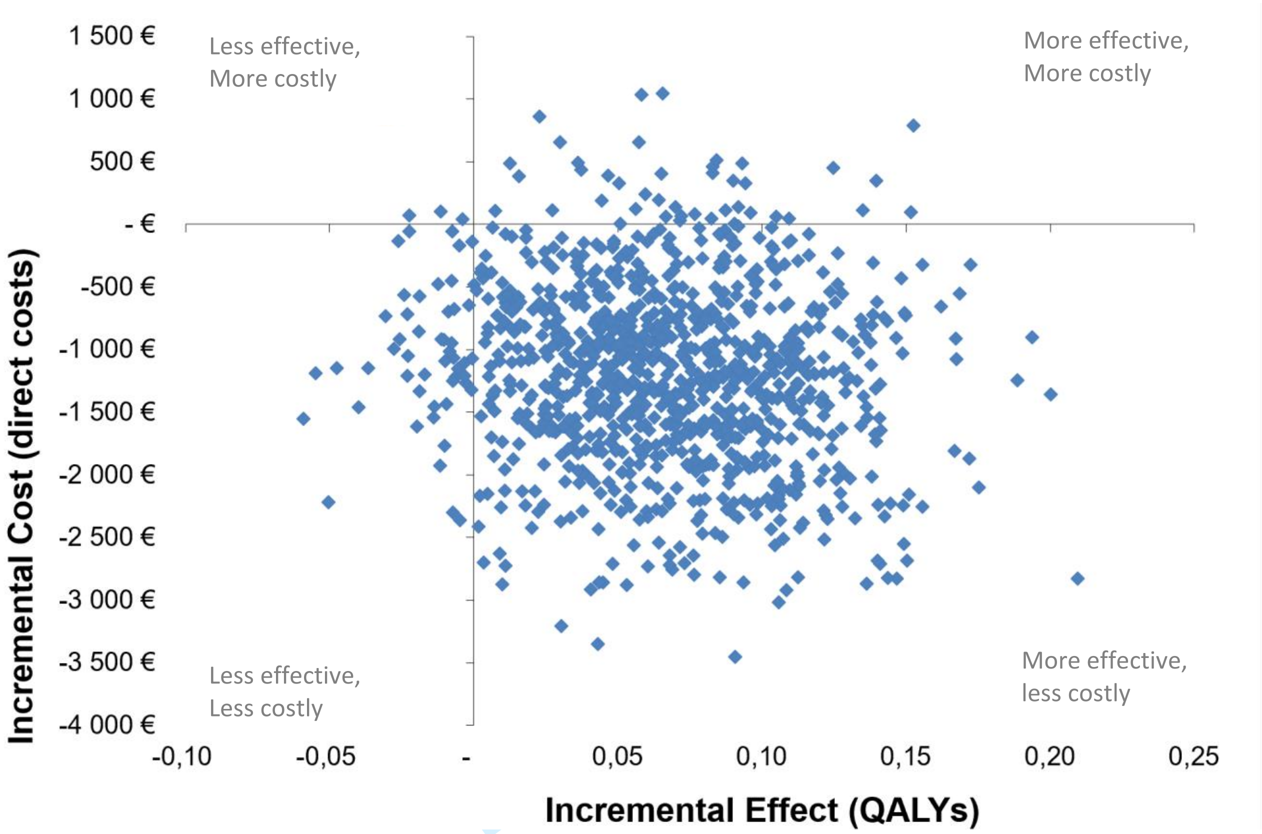

Figure 1

For peer review only

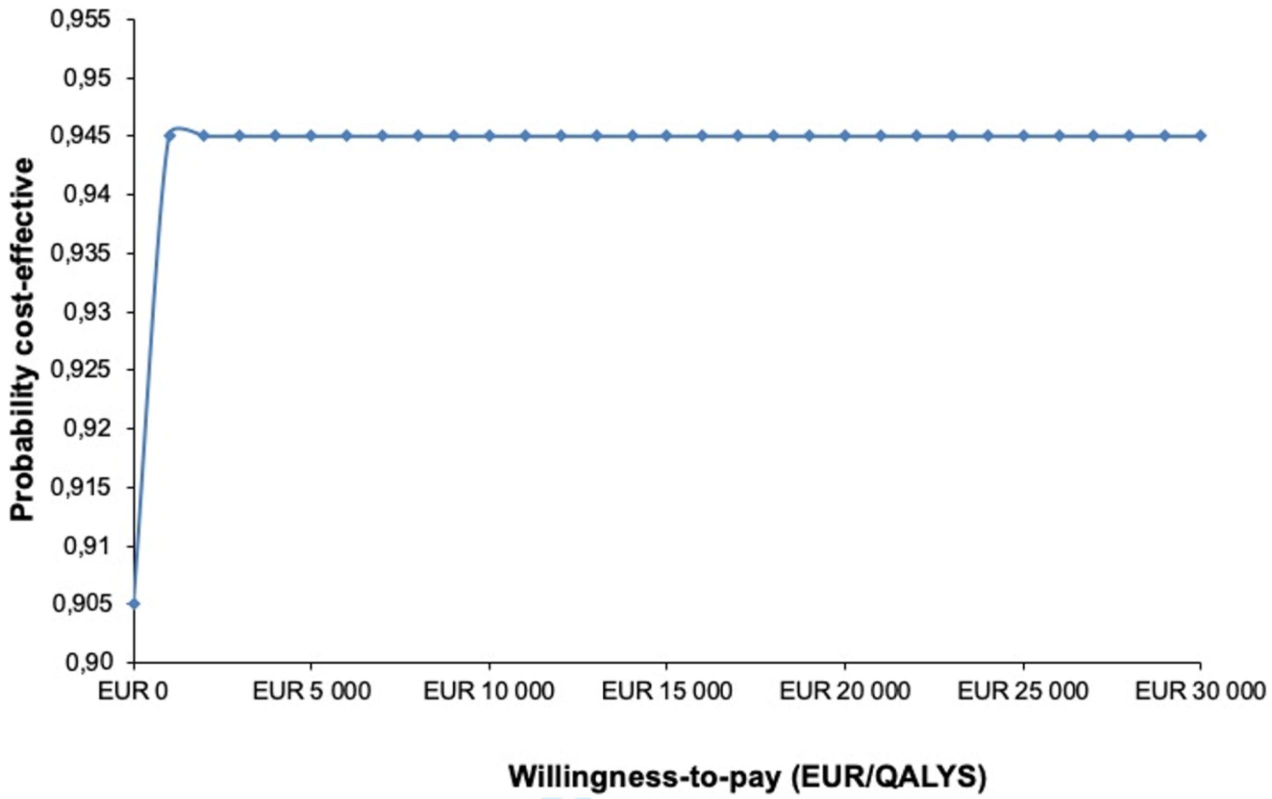
0,955
0,95
0,945
0,94
0,935
0,93
0,925
0,92
0,915
0,91
0,905
0,90
Probability cost-effective
EUR 0
EUR 5 000
EUR 10 000
EUR 15 000
EUR 20 000
EUR 25 000
EUR 30 000
Willingness-to-pay (EUR/QALYS)

Figure 2

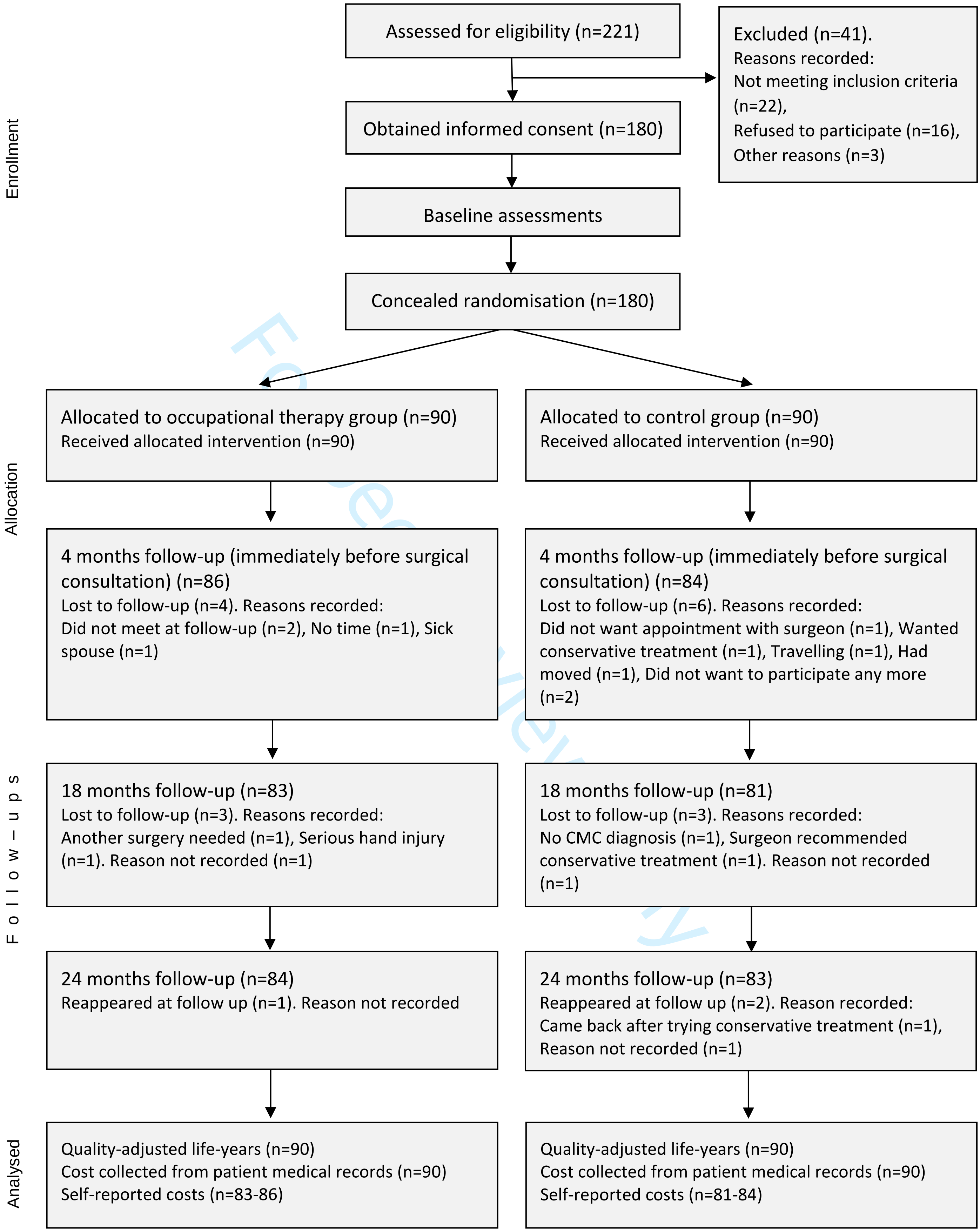

Supplementary figure A

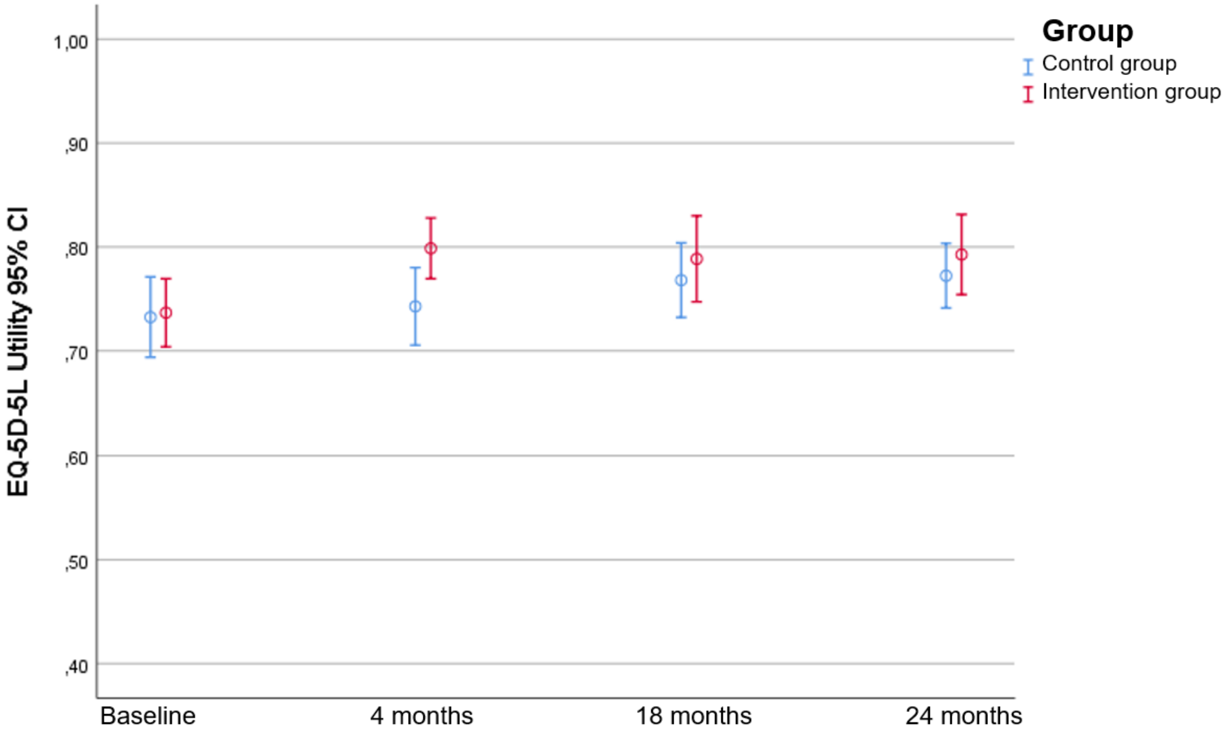

Supplementary figure B

For peer review only

CHEERS 2022 Checklist

**Title**			
Title	1	Identify the study as an economic evaluation and specify the interventions being compared.	page 1
**Abstract**			
Abstract	2	Provide a structured summary that highlights context, key methods, results, and alternative analyses.	pag 2
**Introduction**			
Background and objectives	3	Give the context for the study, the study question, and its practical relevance for decision making in policy or practice.	page 3-4
Methods			
**Health economic analysis plan**	**4**	**Indicate whether a health economic analysis plan was developed and where available.**	**page 3**
Study population	5	Describe characteristics of the study population (such as age range, demographics, socioeconomic, or clinical characteristics).	table 1
Setting and location	6	Provide relevant contextual information that may influence findings.	page 3-4
Comparators	7	Describe the interventions or strategies being compared and why chosen.	page 5
Perspective	8	State the perspective(s) adopted by the study and why chosen.	page 4
Time horizon	9	State the time horizon for the study and why appropriate.	page 4

For peer review only - http://bmjopen.bmj.com/site/about/guidelines.xhtml

(continued)

Discount rate	10	Report the discount rate(s) and reason chosen.	page 6-7
Selection of outcomes	11	Describe what outcomes were used as the measure(s) of benefit(s) and harm(s).	page 6-7
Measurement of outcomes	12	Describe how outcomes used to capture benefit(s) and harm(s) were measured.	page 6-7
Valuation of outcomes	13	Describe the population and methods used to measure and value outcomes.	page 4, page 6-7
Measurement and valuation of resources and costs	14	Describe how costs were valued.	page 7
Currency, price date, and conversion	15	Report the dates of the estimated resource quantities and unit costs, plus the currency and year of conversion.	table 2
Rationale and description of model	16	If modelling is used, describe in detail and why used. Report if the model is publicly available and where it can be accessed.	ua
Analytics and assumptions	17	Describe any methods for analysing or statistically transforming data, any extrapolation methods, and approaches for validating any model used.	page 7-8
Characterising heterogeneity	18	Describe any methods used for estimating how the results of the study vary for subgroups.	ua
Characterising distributional effects	19	Describe how impacts are distributed across different individuals or adjustments made to reflect priority populations.	ua

For peer review only - http://bmjopen.bmj.com/site/about/guidelines.xhtml

(continued)

Characterising uncertainty	20	Describe methods to characterise any sources of uncertainty in the analysis.	page 7
Approach to engagement with patients and others affected by the study	21	Describe any approaches to engage patients or service recipients, the general public, communities, or stakeholders (such as clinicians or payers) in the design of the study.	page 4
**Results**			
Study parameters	22	Report all analytic inputs (such as values, ranges, references) including uncertainty or distributional assumptions.	table 3, figure 1
Summary of main results	23	Report the mean values for the main categories of costs and outcomes of interest and summarise them in the most appropriate overall measure.	page 8-9, table 3
Effect of uncertainty	24	Describe how uncertainty about analytic judgments, inputs, or projections affect findings. Report the effect of choice of discount rate and time horizon, if applicable.	page 9-11
Effect of engagement with patients and others affected by the study	25	Report on any difference patient/service recipient, general public, community, or stakeholder involvement made to the approach or findings of the study	page 4
Discussion			

For peer review only - http://bmjopen.bmj.com/site/about/guidelines.xhtml

(continued)

Study findings, limitations, generalisability, and current knowledge	26	Report key findings, limitations, ethical or equity considerations not captured, and how these could affect patients, policy, or practice.	page 9-11
Other relevant information			
Source of funding	27	Describe how the study was funded and any role of the funder in the identification, design, conduct, and reporting of the analysis	page 13
Conflicts of interest	28	Report authors conflicts of interest according to journal or International Committee of Medical Journal Editors requirements.	page 13

From: Husereau D, Drummond M, Augustovski F, et al. Consolidated Health Economic Evaluation Reporting Standards 2022 (CHEERS 2022) Explanation and Elaboration: A Report of the ISPOR CHEERS II Good Practices Task Force. Value Health 2022;25. doi:10.1016/j.jval.2021.10.008

For peer review only

Is multimodal occupational therapy in addition to usual care cost-effective in people with thumb carpometacarpal osteoarthritis? A cost-utility analysis of a randomized controlled trial

Journal:	*BMJ Open*
Manuscript ID	bmjopen-2022-063103.R3
Article Type:	Original research
Date Submitted by the Author:	04-May-2023
Complete List of Authors:	TVETER, Anne Therese; Diakonhjemmet Hospital Norwegian National Advisory Unit on Rehabilitation in Rheumatology, Center for treatment in rheumatic and musculoskeletal diseases (REMEDY), Department of Rheumatology and Research<br>Kleven, Linn; Norwegian Institute of Public Health<br>Osteras, Nina; Diakonhjemmet Hospital Norwegian National Advisory Unit on Rehabilitation in Rheumatology, Center for treatment in rheumatic and musculoskeletal diseases (REMEDY), Department of Rheumatology and Research<br>Nossum, Randi; St Olavs Hospital Trondheim University Hospital<br>Eide, Ruth Else Mehl; Haukeland University Hospital<br>Klokkeide, Åse; Haugesund Sanitary Association Rheumatism Hospital<br>Matre, Karin Hoegh; Haukeland University Hospital<br>Olsen, Monika; Haugesund Sanitary Association Rheumatism Hospital<br>Kjeken, Ingvild; Diakonhjemmet Hospital Norwegian National Advisory Unit on Rehabilitation in Rheumatology, Center for treatment in rheumatic and musculoskeletal diseases (REMEDY), Department of Rheumatology and Research
**Primary Subject Heading**:	Health economics
Secondary Subject Heading:	Rehabilitation medicine, Rheumatology
Keywords:	HEALTH ECONOMICS, Musculoskeletal disorders < ORTHOPAEDIC & TRAUMA SURGERY, Hand & wrist < ORTHOPAEDIC & TRAUMA SURGERY, RHEUMATOLOGY

I, the Submitting Author has the right to grant and does grant on behalf of all authors of the Work (as defined in the below author licence), an exclusive licence and/or a non-exclusive licence for contributions from authors who are: i) UK Crown employees; ii) where BMJ has agreed a CC-BY licence shall apply, and/or iii) in accordance with the terms applicable for US Federal Government officers or employees acting as part of their official duties; on a worldwide, perpetual, irrevocable, royalty-free basis to BMJ Publishing Group Ltd ("BMJ") its licensees and where the relevant Journal is co-owned by BMJ to the co-owners of the Journal, to publish the Work in this journal and any other BMJ products and to exploit all rights, as set out in our licence.

The Submitting Author accepts and understands that any supply made under these terms is made by BMJ to the Submitting Author unless you are acting as an employee on behalf of your employer or a postgraduate student of an affiliated institution which is paying any applicable article publishing charge ("APC") for Open Access articles. Where the Submitting Author wishes to make the Work available on an Open Access basis (and intends to pay the relevant APC), the terms of reuse of such Open Access shall be governed by a Creative Commons licence – details of these licences and which Creative Commons licence will apply to this Work are set out in our licence referred to above.

Other than as permitted in any relevant BMJ Author's Self Archiving Policies, I confirm this Work has not been accepted for publication elsewhere, is not being considered for publication elsewhere and does not duplicate material already published. I confirm all authors consent to publication of this Work and authorise the granting of this licence.

For peer review only

Is multimodal occupational therapy in addition to usual care cost-effective in people with thumb carpometacarpal osteoarthritis? A cost-utility analysis of a randomized controlled trial

Anne Therese Tveter[1], Linn Kleven[2], Nina Østerås[1], Randi Nossum[3], Ruth Else Mehl Eide[4], Åse Klokkeide[5], Karin Hoegh Matre[4], Monika Olsen[5], Ingvild Kjeken[1]

[1] Norwegian National Advisory Unit on Rehabilitation in Rheumatology, Center for treatment in rheumatic and musculoskeletal diseases (REMEDY), Department of Rheumatology and Research, Diakonhjemmet Hospital, Oslo, Norway

[2] Norwegian Institute of Public Health, Oslo, Norway

[3] St.Olavs Hospital, Trondheim University Hospital, Trondheim, Norway

[4] Haukeland University Hospital, Bergen, Norway

[5] Haugesund Sanitary Association Rheumatism Hospital, Haugesund, Norway

Corresponding author:

Anne Therese Tveter

Center for treatment of rheumatic and musculoskeletal diseases (REMEDY)

Diakonhjemmet Hospital

Postboks 23 Vinderen, 0319 Oslo, Norway

a.t.tveter@medisin.uio.no, https://orcid.org/0000-0003-1701-9835

For peer review only - http://bmjopen.bmj.com/site/about/guidelines.xhtml

ABSTRACT

Objective: The aim was to evaluate the cost-utility of a 3-month multimodal occupational therapy intervention in addition to usual care in patients with thumb carpometacarpal osteoarthritis (CMC1 OA).

Methods: A cost-utility analysis was performed alongside a multicentre randomized controlled trial including three rheumatology departments in Norway. A total of 180 patients referred to surgical consultation due to CMC1 OA were randomized to either multimodal occupational therapy including patient education, hand exercises, assistive devices, and orthoses (n=90), or usual care receiving only information on OA (n=90). The outcome measure was quality-adjusted life-years (QALYs) derived from the generic questionnaire EQ-5D-5L over a 2-year period. Resource use and health-related quality of life of the patients were prospectively collected at baseline, 4, 18, and 24 months. Costs were estimated by taking a healthcare and societal perspective. The results were expressed as incremental cost-effectiveness ratios, and a probabilistic sensitivity analysis with 1 000 replications following intention-to-treat principle was done to account for uncertainty in the analysis.

Results: During the 2-year follow-up period, patients receiving multimodal occupational therapy gained 0.06 more QALYs than patients receiving usual care. The mean (SD) direct costs were € 3227 (3546) in the intervention group and € 4378 (5487) in the usual care group, mean difference €-1151 (95% CI -2564, 262). The intervention was the dominant treatment with a probability of 94.5% of being cost-effective given the willingness-to-pay threshold of € 27 500.

Conclusions: The within-trial analysis demonstrated that the multimodal occupational therapy in addition to usual care was cost-effective at 2 years in patients with CMC1 OA.

Trial registration number: ClinicalTrials.gov (NCT01794754)

Keywords: hand osteoarthritis, occupational therapy, cost-effectiveness, cost-utility, quality-adjusted life-years (QALYs)

For peer review only - http://bmjopen.bmj.com/site/about/guidelines.xhtml

Strength and limitations of the study

- A strength of the study is the involvement of a patient research partner throughout the conduct of the study, although it may be considered a limitation that only one partner was involved.
- Self-reported costs combined with several months between assessments may have biased the total costs of the study.
- We were unable to include medication costs, travel expenses or purchase of technical or medical equipment in the analyses due to imprecise reporting.

For peer review only

INTRODUCTION

Hand osteoarthritis (OA) is the most common joint disease, [1] with the subcategory thumb carpometacarpal osteoarthritis (CMC1 OA) affecting the base of the thumb. [2] Approximately 22% of people aged ≥50 years have symptomatic CMC1 OA. [3] Compared to OA in the other finger joints, CMC1 OA is associated with more pain and functional limitations, negatively affecting work ability and quality of life. [2,4,5]

There is a common misconception that hand OA is a normal part of aging, and that there is 'nothing to be done'. [6] Although there is no cure for OA, the European League Against Rheumatism (EULAR) recommends that all people with hand OA should be offered a multimodal intervention consisting of information, hand exercises, and assistive devices as first-line treatment. [7] In addition, orthoses should be provided for patients with CMC1 OA. [7] Pharmacological therapy is recommended as a symptom relieving supplemental intervention, whereas surgery should only be considered when other treatments have failed to provide sufficient pain relief. [7] The multimodal intervention is shown to have a statistically significant beneficial short-term effect on pain[8,9] and hand function[9] compared to or in addition to usual care. However, there is an evidence-to-practice gap regarding this first-line multimodal intervention, with only 20% reporting to have received this treatment before being referred to surgical consultation. [10] This intervention also presents a tendency towards reduction and delay in surgery rate over a 2-year period. [11] Although not significant, this tendency may still be of clinical importance if shown to be cost-effective.

For peer review only - http://bmjopen.bmj.com/site/about/guidelines.xhtml

Oppong et al performed a trial-based economic evaluation on joint protection only, hand exercises only and a combination of joint protection and hand exercises compared to leaflet and advice over a 12-month period in patients with hand OA. [12] They found hand exercises to be the most cost-effective alternative. [12] In the study by Adams however, providing orthoses only was found to be neither effective nor cost-effective. [13] Thus, the results regarding the different components of recommended treatment are not conclusive. To the best of our knowledge, no studies have assessed the cost-utility of the different components combined in a multimodal intervention following the EULAR recommendations for the treatment of hand OA or CMC1 OA.

The aim of this study was to perform a cost-utility analysis of a 3-month multimodal occupational therapy intervention in addition to usual care in patients with CMC1 OA referred to surgical consultation. The outcome measure was quality adjusted life-years (QALYs), and the costs were estimated both as direct and indirect costs and societal costs over a 2-year period taking a healthcare and societal perspective.

METHODS

Study design and setting

A cost-utility analysis (CUA) was performed alongside a multicentre RCT including rheumatology departments in three Norwegian hospitals (St. Olav's Hospital in Trondheim, Haukeland University Hospital in Bergen, and Haugesund Rheumatism Hospital) and is described in the protocol article. [11,14] In Norway, most hospitals (including the three hospitals involved in this trial) are public and owned by the Norwegian government. The trial was approved by the Norwegian Regional Ethical Committee (2012/2265/REK sør-øst C).

Patient and Public Involvement

A patient research partner from the Patient Research Panel at Diakonhjemmet Hospital, who had lived with hand osteoarthritis for several years, was involved throughout the project. She participated in

For peer review only - http://bmjopen.bmj.com/site/about/guidelines.xhtml

project meetings where she gave input to the project plan, recruitment procedure, and information material as well as feedback on the interpretation and dissemination of the results.

Participants and randomization

Patients referred by their general practitioner for surgical consultation due to CMC1 OA at the three rheumatology departments between 2013 and 2015 were considered eligible for the study if they could speak Norwegian and had no cognitive deficits. Eligible patients received information about the study, a written consent form and a questionnaire by post. Patients who agreed to participate were scheduled for an appointment with an occupational therapist (OT) at the hospital within 2 weeks after referral. After a baseline assessment, the patients were randomized to either a 3-month multimodal occupational therapy intervention or usual care. In Norway, the waiting period between referral and the actual surgical consultation was at the time this study was conducted between 4 and 6 months, and the intervention was conducted during this waiting period. Patients were assessed again immediately before surgical consultation (approximately 4 months after baseline assessment), and 18 and 24 months after the baseline assessment (Supplementary figure A). The sample size of 180 participants was estimated based on an expected surgery rate of 70% during the 2-year follow-up, which was the primary endpoint of the RCT. [11,14]

Intervention

During the waiting period between referral and surgical consultation, the intervention group received a multimodal occupational therapy intervention in line with recommended first-line treatment. [7] In short, at the baseline assessment, the patients received written and oral information on hand OA, ergonomic principals, and the use of assistive devices by an OT. They were instructed in a hand exercise program to be performed at home three times per week for 12 weeks. They all received five common assistive devices (a bread knife and vegetable knife with built-up handles, an enlarged grip for opening bottles, a key for opening jars [the Jar Key], and a self-opening pair of scissors) for use when needed, and daytime and nighttime orthoses that they were encouraged to use as much as possible. [14] The assistive

For peer review only - http://bmjopen.bmj.com/site/about/guidelines.xhtml

devices were provided at no cost to the patients, while they had to pay a deductible for the orthoses. The patients in the intervention group consulted the OT twice during this intervention period: at the baseline assessment and again after 2 weeks to adjust the orthoses and exercise program, implying that the intervention was based mostly on self-management. Patients referred for bilateral hand OA received treatment for both hands. At the follow-up assessments (4, 18, and 24 months), the patients in the intervention group could receive new orthoses, or the OTs could adjust the ones they already had.

The control group received usual care, which generally means staying on the waiting list for consultation in specialist healthcare, i.e., receiving no treatment in specialist healthcare. For the purpose of this study, the control group were assessed at the hospital at baseline and all follow-up time points by an OT. They received general written and oral information about hand OA. The control group had the same possibility as the intervention group to ask questions and be given advice.

If necessary, patients in both groups could independently seek treatment in primary healthcare, such as a consultation with general practitioner or other healthcare personnel (this information was collected as part of this study). Both groups had a surgical consultation with a medical specialist approximately 4 months after the baseline assessment. All patients paid a deductible for the assessment consultations (a maximum of €259 per year).

Randomization and blinding

Patients were randomized using a computer-generated list with a block size of 10, stratified by hospital. Envelopes were opened by the patients after receiving information on hand OA and completing baseline assessment. The group affiliation was known to both the patients and the OTs.

Data collection

Health outcome

The health-related quality of life (HRQoL) was measured at baseline, 4, 18, and 24 months using the generic instrument EQ-5D-5L (EuroQol 5-dimension, www.euroqol.org).[15] The EQ-5D-5L is a preference weighted measure of HRQoL based on five dimensions: mobility, self-care, daily activities, pain/discomfort,

For peer review only - http://bmjopen.bmj.com/site/about/guidelines.xhtml

and anxiety/depression. For each dimensions the patient assesses five possible levels of problems, from no problems to severe. [15] Quality-adjusted life-years (QALYs) were derived from the EQ-5D-5L utility score, using the preference score from a UK population. Since there is no Norwegian tariff, the recommendation in Norway is to use the British tariff. [16] QALYs are weighted for time (years of life) with an index that express the HRQoL (utilities) on a scale anchored at 0 (health state equivalent to death) and 1 QALY (full health).

Resource use and costs

Healthcare utilization was collected from different sources at baseline, 4, 18 and 24 months. The OT reported the number of consultations related to the intervention at each assessment, whereas surgical procedures and post-operative treatment were collected from patients' medical record. In addition, the use of primary and specialist healthcare was self-reported by the patient. We have included the costs related to the OT assessment at baseline, 4, and 18 months for both groups (OT assessment at 24 months were not included as the usual care group then received the intervention). Although the OT assessment was done for study purposes, it was included in the cost estimates because we cannot rule out a possible positive effect of an appointment with an OT in specialist healthcare for the control group. We have not included medication costs, travelling expenses or purchase of technical or medical equipment (except assistive devices provided to patients in the intervention group) due to lack of or imprecise reporting. Many patients reported using medication when needed, making it impossible to calculate costs. Travelling expenses were not collected, and with regard to technical or medical equipment, it was not possible to determine if the patients had bought the equipment before or during the trial.

Data on sickness absence related to surgery were collected from patient medical records. The number of hours of informal help at home due to OA (from spouse, family, or friends) and the productivity loss (due to any reason of sickness absence and disability benefits, excluding sickness absence related to surgery) were self-reported. If not explicitly indicated by the patient, we expected that the productivity loss remained the same between assessment points, thus, if the patient reported sickness absence of 50% at 4 months and 100% at 18 months, this was calculated as 50% productivity loss between 4 and 18 months, and 100% between 18 and 24 months.

For peer review only - http://bmjopen.bmj.com/site/about/guidelines.xhtml

The costs of specialist healthcare visits and surgery were calculated using diagnosis-related group (DRG) weights from 2019. DRG is a coding system used for administration of both clinical and financial activity in specialist healthcare. The DRG costs were estimated by multiplying the cost weighting for the specific DRG group of the patient with the unit cost of 2019. [17] Sickness absence and disability benefits were valued in the same way for all patients. Costs related to absence from work were estimated from official statistics on the average wage by sex and age group in 2019. The average daily productivity loss was valued as € 336 including societal costs of 40% and was adjusted for the percentage of sickness absence/disability benefits. [18] The number of hours of informal help at home was valued at a minimum wage of € 18 per hour. [19] All costs are estimated in Euros, using the exchange rate 0.10 (€ 1 = NOK 10) from 2019.

Direct costs included costs related to specialist healthcare (medical specialist, occupational therapist, physical therapist, nurse, radiographs, surgery, assistive devices) and primary healthcare (general practitioner, physical therapist, manual therapist, acupuncturist, blood samples). Indirect costs included productivity loss due to sickness absence (due to surgery or for other reasons) and costs related to help with chores at home.

Statistics

Differences in QALYs between the two groups were estimated using the trapezoidal method (area under the curve). [20] To illustrate the statistical uncertainty surrounding the incremental cost-effectiveness ratio (ICER), a probabilistic sensitivity analysis with 1 000 replications was performed using the bootstrap resampling method. The bootstrapped costs and effects can be illustrated on a cost-effectiveness plane (CE plane). The CE plane (Figure 1) are divided into four quadrants; where a new treatment that is more effective and less costly is plotted in the southeast quadrant, while one more costly and less effective is plotted in the northwest quadrant. Figure 2 shows the cost-effectiveness acceptability curve (CEAC), which presents the probability that the intervention is cost-effective in addition to usual care for hand OA. The willingness-to-pay threshold was set to € 27 500 (275 000 NOK) due to the low severity of the condition. [21]

For peer review only - http://bmjopen.bmj.com/site/about/guidelines.xhtml

Between-group differences in mean costs and mean effects were analyzed using Independent sample t-test. Significance level was set to 0.05. Statistical analyses were performed using Excel, SPSS version 26 and Stata version 16.

RESULTS

Baseline characteristics

A total of 180 patients were randomized to either multimodal occupational therapy (n=90) or usual care (n=90). The mean age across both groups was 63 years (SD 7.6) and 79% of the patients were women. We found no between-group differences in any of the baseline characteristics including utility score, medication use, or percentage of patients working full- or part-time (Table 1).

For peer review only - http://bmjopen.bmj.com/site/about/guidelines.xhtml

Table 1 Demographic variables for patients referred to surgical consultation due to carpometacarpal osteoarthritis (n = 180). All information was collected at the baseline assessment

		Intervention group (n=90)	Control group (n=90)
Age, years, mean (SD)		62.8 (7.5)	63.3 (7.8)
Female sex, *n* (%)		73 (81)	69 (77)
Referred hand, *n* (%)			
	Right	31 (34)	17 (19)
	Left	24 (27)	29 (32)
	Both	35 (39)	44 (49)
Education level, *n* (%)			
	Primary school	14 (16)	20 (22)
	High school	44 (49)	39 (43)
	University/college ≤ 4 y	17 (19)	19 (21)
	University/college > 4 y	15 (16)	12 (13)
Living alone, *n* (%)		17 (19)	18 (20)
Working full/part-time, *n* (%)		48 (53)	43 (48)
Years with symptoms, median (IQR)		5 (2–10)	5 (2–10)
Presence of radiographic subluxation of the CMC joint		58 (64)	55 (61)
Comorbidity‡, *n* (%)		61 (68)	56 (62)
Analgesics use (anti-inflammatory or pain relief medication), *n* (%)		57 (63)	57 (63)
Pain at rest (NRS 0–10, 0 = no pain), median (IQR)		3 (1–4)	3 (2–5)
EQ-5D-5L utility score (1=best health), mean (SD)		0.74 (0.16)	0.73 (0.18)

SD= standard deviation; IQR= interquartile range (25th and 75th percentile); CMC= carpometacarpal joint; NRS= numeric rating scale; Pain at rest was reported for referred hand (the mean of both hands if bilateral referral); ‡ Comorbidities was categorized as present if they had any of 16 predefined comorbidities.

For peer review only - http://bmjopen.bmj.com/site/about/guidelines.xhtml

Missing data

A total of 18 patients (7 in the intervention group and 11 in the control group) had missing HRQoL utility score at one or more timepoints. For these missing values we used 'last observation carried forward'. Sensitivity analyses showed that the average HRQoL utility scores remained the same for both groups without imputation of these missing values. Missing self-reported costs were not imputed (n=16). The costs related to surgery were collected from patient medical records and were not encumbered by missing data.

Cost-utility analysis

The total between-group difference in QALYs was 0.06 (95% CI -0.02, 0.15) after 24 months, in favor of the intervention group. The distribution of EQ-5D-5L utility score across the different time points for the two groups are shown in Supplementary figure B. The mean healthcare costs and productivity loss for both treatment groups are presented in Table 2.

For peer review only - http://bmjopen.bmj.com/site/about/guidelines.xhtml

Table 2 Mean direct and indirect healthcare costs (mean and standard deviation) per patient for the intervention and control group (n=180). The table also show unit price and the resource use (n).

		Unit	Unit price, €	n	Intervention group Mean (SD) per patient, €	n	Control group Mean (SD) per patient, €	Mean difference (95% CI), €
Direct costs	***Specialist healthcare***							
	Medical specialist	Per visit	205[a]	136	309 (268)	169	384 (365)	-75 (-169, 19)
	Occupational therapist including adaptation of orthoses	Per visit	246[a]	209	536 (318)	65	158 (367)	377 (275, 478)
	Occupational / Physical therapist	Per visit	205[a]	193	428 (350)	333	837 (866)	-355 (-549, -160)
	Nurse	Per visit	205[a]	1	16 (98)	9	48 (189)	-32 (-76, 13)
	Inpatient surgery	Per surgery	8 435[a]	2	187 (1 250)	6	562 (2 465)	-375 (-951, 201)
	Day-bed surgery	Per surgery	2 251[a]	20	500 (941)	27	675 (1 190)	-175 (-491, 141)
	Radiographs	Per visit	149 [e]	90	149 (0)	90	149 (0)	-
	Assistive devices	Per person	67 [e]	90	67 (0)	0	-	-
	Primary healthcare							
	General practitioner	Per visit	32[b]	91	32 (94)	130	46 (104)	-14 (-43, 15)
	Physical therapist	Per visit	31[c]	187	64 (288)	158	54 (160)	10 (-59, 79)
	Manual therapist	Per visit	52[c]	0	-	16	10 (90)	-
	Acupuncturist	Per visit	47[e]	22	11 (64)	44	23 (132)	-11 (-42, 19)
	Blood samples	Per test	7[b]	14	1 (6)	24	2 (6)	-1 (-2, 1)
Indirect costs	Sickness absence due to surgery	Days	336[d]	65	1 212 (3 815)	64	1 199 (3 817)	186 (-1 104, 1 141)
	Costs related to help with chores at home	Hours	18[d]	3261	652 (1 722)	4612	922 (3 089)	-270 (-1 006, 465)
	Sickness absence and disability benefits (not sickness absence due to surgery)	Days	336[d]	10351	38 299 (63 029)	12 249	45 433 (66 320)	-7 133 (-26 167, 1 190)

[a] DRG (diagnosis-related group system); [b] List of reimbursement codes; [c] Rates for physiotherapists; [d] Official statistics of average wage; [e] Based on real costs. All costs are given in Euro (€1=NOK10) and adjusted to 2019.

For peer review only - http://bmjopen.bmj.com/site/about/guidelines.xhtml

The mean cost of multimodal occupational therapy intervention (baseline information on hand OA, instruction in hand exercises, assistive devices, and customization of orthoses, and adjustment of hand exercises and orthoses after 14 days) was approximately € 500 per patient. Surgery accounted for the largest between-group difference related to direct healthcare costs with 22 surgical procedures in the intervention group and 33 surgical procedures in the control group, resulting in a €550 difference in surgical cost per patient between the two groups. The use of physiotherapy/occupational therapy also constituted a high cost. However, almost one-third of these costs were related to the predetermined assessment points (4, 18 and 24 months) that were part of the study but not the actual OT intervention (€375 per patient in the control group and €307 per patient in the intervention group).

Productivity loss due to sickness absence or disability benefits was the largest contributor to the total costs in both groups. However, at baseline, only 7% of the sickness absence in both groups was reported to be due to hand OA, and the percentages at 4, 18, and 24 months were 6%, 2% and 0.5%, respectively. Similar numbers of patients in the two groups were retired at baseline (34% in the control group versus 32% in the intervention group), 4 months (36% versus 34%), and 24 months (42% versus 43%). However, more patients in the intervention group (43%) retired between 4 and 18 months than in the control group (36%), indicating that fewer patients contributed to productivity loss in the intervention group during this period.

Table 3 shows the mean incremental costs and incremental effects associated with direct and indirect healthcare costs. In the case of a negative ICER, the evaluated treatment is either dominant (more effective and less costly) or dominated (less effective and more costly). The intervention in this study was the dominant treatment (Figure 1) with a 94.5% probability of being cost-effective given the willingness-to-pay threshold (Figure 2).

For peer review only

Table 3 Cost-utility analysis of mean costs (SD), mean effects (SD), incremental differences in costs and effects (95% CI), and incremental cost-effectiveness ratio (ICER) for direct and total direct and indirect costs (n=180)

	Intervention group (n=90)		Control group (n=90)				
	**Mean cost (SD)**	**Mean effect* (SD)**	**Mean cost (SD)**	**Mean effect* (SD)**	**Incremental cost (95% CI)**	**Incremental effect (95% CI)**	**ICER**
Direct costs	3 227 (3 546)	1.58 (0.28)	4 378 (5 487)	1.51 (0.28)	-1 151 (-2 564, 262)	0.06 (-0.02, 0.15)	Dominant
Total costs (direct and indirect costs)	46 617 (65 499)	1.58 (0.28)	56 304 (68 872)	1.51 (0.28)	-9 688 (-9 949, 29 324)	0.06 (-0.02, 0.15)	Dominant

Direct costs, **€**: self-reported use of primary and specialist healthcare, including surgery;

Indirect costs, **€**: productivity loss from sickness absence due to surgery, costs related to informal help at home, productivity loss from sickness absence and disability benefits excluding sickness absence due to surgery

Total cost, **€**: direct costs + indirect costs

* Mean effect without imputations; Intervention group 1.58 (0.28), Control group 1.51 (0.28)

SD= standard deviation

For peer review only

For peer review only - http://bmjopen.bmj.com/site/about/guidelines.xhtml

DISCUSSION

The aim of this study was to evaluate the cost-utility of a 3-month multimodal occupational therapy intervention in patients with CMC1 OA in a 2-year period. Our results demonstrate that the intervention, consisting of patient education, hand exercises, orthoses, and assistive devices, has the highest probability of being cost-effective, given the willingness-to-pay threshold (€ 27 500/QALY). The results indicate that the intervention is both less costly and more effective when provided alongside usual care. This study is one of few studies to evaluate the cost-utility of conservative treatment in hand OA, and the only study to evaluate the cost-utility of the 2018 EULAR guidelines on first-line treatment for CMC1 OA. Our findings add to the evidence base by demonstrating that multimodal occupational therapy is a cost-effective alternative for the management of CMC1 OA.

The results of this study are in line with a previous study showing that hand exercises were more cost-effective than leaflet and advice in patients with hand OA. [12] In contrast, the study by Oppong et al. failed to show that the combination of hand exercises and use of assistive devices (joint protection classes[22]) were more cost-effective than hand exercises alone. [12] Similarly, the use of orthoses alone was shown to be neither effective nor cost-effective. [13] The results from the current study should, however, be replicated in new studies taking into account also travelling cost, medication, and technical and medical equipment.

In our study, we did not separately evaluate the different parts of the recommended first-line treatment. It is possible that we would have found the same results as Oppong et al., [12] given that hand exercises only entail costs related to the time used by health personnel to educate the patient, whereas both orthoses and assistive devices require an additional cost. On the other hand, the combination of the recommended first-line treatment was found to have a significant 3-month short-term effect on reducing pain and improving hand function in these patients, [8] and should be offered to all patients with CMC1 OA as an effective and cost-effective alternative. Due to the self-managing nature of the intervention, the mean cost related to the multimodal occupational therapy intervention was low, approximately €500 per patient. However, the current study was conducted in specialist healthcare, and there are other options for

For peer review only - http://bmjopen.bmj.com/site/about/guidelines.xhtml

delivering this intervention that may be even more cost-effective. The treatment of patients with OA should mainly be conducted in primary healthcare, possibly reducing the costs to some extent. However, due to the lack of availability of treatment options and long travel distances in parts of the country, more cost-effective options for delivery of treatment could be further explored, e.g. using a smart phone application as shown in a Swedish study on digital first-line treatment for hip and knee OA. [23] We are currently evaluating the effect and cost-utility of a newly developed smartphone app (the Happy Hands app) which is designed to make recommended treatment available to people with hand OA. [24]

Surgery constituted a large cost in this within-trial. Although we found a tendency towards reduction in surgical procedures in the intervention group compared to the control group, this result was not statistically significant. [11] Still, the between-group difference in mean surgical costs (€ 550) was higher than the mean cost of the multimodal occupational therapy intervention alone (€ 500). Thus, although the difference in surgical rates between the two groups were found to be non-significant, [11] the difference may still be regarded as important in a healthcare and societal perspective as surgical procedures may be prone to more adverse events compared to multimodal occupational therapy. [11] Most likely, the avoidance of surgery has the highest impact on the cost-effectiveness of the intervention.

Productivity loss represented the largest contributor to the total costs in both groups. The between-group difference may to some extent be attributed to a higher retirement rate in the intervention group. Although most participants were of retirement age, we do not know the reason for retirement and thsi should therefore be reported in future studies. Additionally, to avoid bias, register data should be used for more accurate reporting of sickness absence and disability benefits.

The patients reported to have OA complaints for a median of 5 years, and more than 60% showed radiographic signs of subluxation of the carpometacarpal joint, indicating advanced disease progression. However, only 21% of the patients had visited a physiotherapist or an OT before referral to surgical consultation. [10] Thus, referral to a multimodal occupational therapy intervention at an earlier stage in the disease course may be a possible step towards reducing the costs related to healthcare use.

For peer review only - http://bmjopen.bmj.com/site/about/guidelines.xhtml

The current study is the first study assessing the cost-utility of recommended multimodal treatment in patients with CMC1 OA. It is considered a strength that the study was conducted alongside an RCT. The study also has some limitations. Almost 80% of the sample were women, which represents a higher proportion of women than the usual gender distribution in hand OA. The results may therefore not be representative for men with hand OA. The self-reported costs and the 14-month time frame between the reporting may have biased the total costs. This has most likely contributed to lower overall costs, as patients may not remember to report all consultations with healthcare personnel, especially in the time period between 4 and 18 months. On the other hand, the costs related to surgery were collected from patient medical records and will not be encumbered by the same bias. A total of 16 patients had missing data throughout the study. There may have been systematic reasons for these patients being missing, which could have affected the outcome. However, there were more missing data in the control group than the intervention group, indicating that the between-group difference could have been larger if all patients had reported direct and indirect costs. The results may have been biased by the method of imputation of missing HRQoL utility values. However, due to low number of missing values and sensitivity analyses showing no difference in mean score whether using 'Last observation carried forward' or not, we did not go any further with more advanced methods like multiple imputation. We were unable to include medication costs, travel expenses or purchase of technical or medical equipment, except for the assistive devices provided as part on the intervention, in the analyses. This is not in line with the most recent recommendations for cost-utility analyses[25] and may have biased the results by possibly underestimating the total costs. However, the equal distribution of medication use across the two groups imply that this do not constitute a large bias in the comparison of results. As with all conservative treatments, the patients knew that they received the intervention, and this may be a potential bias improving the effect measure. Measurement properties of the EQ-5D-5L has not been assessed in patients with CMC OA. The HRQoL measure might also capture QoL related to other conditions than only CMC OA. Although the follow-up sessions were similar for both groups, the results may have been influenced by the fact that patients in the intervention group could receive adjustment of orthoses or new orthoses at these sessions. This may have influenced the results. The involvement of a patient research partner with lived experience on hand

For peer review only - http://bmjopen.bmj.com/site/about/guidelines.xhtml

osteoarthritis is a strength, however, the study would have benefited from input from more than one partner. Future studies should evaluate the cost-utility of an even longer follow-up time as there is a lack of knowledge on surgical rate of these patients in the long term.

CONCLUSION

The within-trial analysis demonstrated that multimodal occupational therapy was cost-effective in addition to usual care in patients with CMC1 OA, when taking a healthcare and societal perspective. The intervention was the dominant treatment with a 94.5% probability of being cost-effective at 2 years, given the willingness-to-pay threshold of € 27 500/QALY. The low cost related to the intervention supports the use of this first-line treatment in all patients with CMC1 OA.

Acknowledgements

We would like to acknowledge patient research partner Øyvor Andreassen for her contribution throughout the project. We would also like to thank professor Tore K. Kvien for reading and commenting on the manuscript. The project has been acknowledged at the EULAR Congress with the HPR Abstract Award in 2019 for the primary outcome results and the HPR Abstract Award in 2020 for the results presented in this article.

Author contribution

The authors have made substantial contributions to the concept or design of the work (IK, RN, REME, ÅK, KHM, MO, NØ), the acquisition of data (RN, REME, ÅK, KHM, MO), analysis (IK, ATT, NØ, LK) and interpretation of data (IK, ATT, NØ, LK). All authors have contributed to the drafting and critical revision of the work (IK, RN, REME, ÅK, KHM, MO, NØ, ATT, LK), final approval (IK, RN, REME, ÅK, KHM, MO, NØ, ATT, LK) and agree to be accountable for all aspects of the work (IK, RN, REME, ÅK, KHM, MO, NØ, ATT, LK).

Funding statement

For peer review only - http://bmjopen.bmj.com/site/about/guidelines.xhtml

This work was supported by the South-Eastern Norway Regional Health Authority (2015109) and Norges Forskningsråd (328657). The funding authorities had otherwise no involvement in the study. This research received no specific grant from any funding agency in the public, commercial or not-for-profit sector.

Conflict of interest

None of the authors have any financial or personal conflicts of interest.

Data sharing statement

Data are available upon reasonable request to the project manager (Ingvild Kjeken, Ingvild. kjeken@diakonsyk.no)

Ethics Statement

The trial was approved by the Norwegian Regional Ethical Committee (2012/2265/REK sør-øst C). All participants gave a written informed consent before taking part in the study.

For peer review only

Figure legends

Supplementary figure A Study flow diagram of a 2-year study period in patients referred to surgical consultation due to carpometacarpal osteoarthritis.

Supplementary figure B EQ-5D-5L utility score across the different timepoints, divided by group.

Figure 1 Cost-effectiveness (CE) plane of the ICERs of multimodal occupational therapy (intervention group) in addition to usual care (control group) were recalculated with 1 000 replications of the study data using the bootstrap resampling method. The intervention is at the origin.

Figure 2 shows a cost-effectiveness acceptability (CEA) curve for direct costs, representing the probability that the multimodal intervention is cost-effective in addition to usual care. At a willingness-to-pay threshold of € 27 500/QALY, the probability of multimodal intervention being cost-effective was 94.5%.

For peer review only

For peer review only - http://bmjopen.bmj.com/site/about/guidelines.xhtml

References

1. Marshall M, Watt FE, Vincent TL, Dziedzic K. Hand osteoarthritis: clinical phenotypes, molecular mechanisms and disease management. *Nature Reviews Rheumatology.* 2018;14(11):641-656:10.1038/s41584-018-0095-4
2. Bijsterbosch J, Visser W, Kroon HM, Stamm T, Meulenbelt I, Huizinga TW, et al. Thumb base involvement in symptomatic hand osteoarthritis is associated with more pain and functional disability. *Annals of the rheumatic diseases.* 2010;69(3):585-587:10.1136/ard.2009.104562
3. Marshall M, Peat G, Nicholls E, van der Windt D, Myers H, Dziedzic K. Subsets of symptomatic hand osteoarthritis in community-dwelling older adults in the United Kingdom: prevalence, inter-relationships, risk factor profiles and clinical characteristics at baseline and 3-years. *Osteoarthritis and cartilage.* 2013;21(11):1674-1684:10.1016/j.joca.2013.08.004
4. Spacek E, Poiraudeau S, Fayad F, Lefevre-Colau MM, Beaudreuil J, Rannou F, et al. Disability induced by hand osteoarthritis: are patients with more symptoms at digits 2-5 interphalangeal joints different from those with more symptoms at the base of the thumb? *Osteoarthritis and cartilage.* 2004;12(5):366-373:10.1016/j.joca.2004.01.005
5. Kwok WY, Vliet Vlieland TP, Rosendaal FR, Huizinga TW, Kloppenburg M. Limitations in daily activities are the major determinant of reduced health-related quality of life in patients with hand osteoarthritis. *Annals of the rheumatic diseases.* 2011;70(2):334-336:https://doi.org/10.1136/ard.2010.133603
6. Hill S, Dziedzic KS, Ong BN. The functional and psychological impact of hand osteoarthritis. *Chronic Illn.* 2010;6(2):101-110:10.1177/1742395309345614
7. Kloppenburg M, Kroon FP, Blanco FJ, Doherty M, Dziedzic KS, Greibrokk E, et al. 2018 update of the EULAR recommendations for the management of hand osteoarthritis. *Ann Rheum Dis.* 2018:10.1136/annrheumdis-2018-213826
8. Tveter AT, Østerås N, Nossum R, Eide REM, Klokkeide Å, Hoegh Matre K, et al. Short-term effects of occupational therapy on hand function and pain in patients with carpometacarpal osteoarthritis: secondary analyses from a randomized controlled trial. *Arthritis care & research.* 2020:10.1002/acr.24543
9. Deveza LA, Robbins SR, Duong V, Bennell KL, Vicenzino B, Hodges PW, et al. Efficacy of a Combination of Conservative Therapies vs an Education Comparator on Clinical Outcomes in Thumb Base Osteoarthritis: A Randomized Clinical Trial. *JAMA Intern Med.* 2021;181(4):429-438:10.1001/jamainternmed.2020.7101
10. Gravas EMH, Tveter AT, Nossum R, Eide REM, Klokkeide A, Matre KH, et al. Non-pharmacological treatment gap preceding surgical consultation in thumb carpometacarpal osteoarthritis - a cross-sectional study. *BMC musculoskeletal disorders.* 2019;20(1):180:10.1186/s12891-019-2567-3
11. Gravås EMH, Østerås N, Nossum R, Eide REM, Klokkeide Å, Matre KH, et al. Does occupational therapy delay or reduce the proportion of patients that receives thumb carpometacarpal joint surgery? A multicentre randomised controlled trial. *RMD Open.* 2019;5(2):e001046:10.1136/rmdopen-2019-001046
12. Oppong R, Jowett S, Nicholls E, Whitehurst DG, Hill S, Hammond A, et al. Joint protection and hand exercises for hand osteoarthritis: an economic evaluation comparing methods for the analysis of factorial trials. *Rheumatology (Oxford, England).* 2015;54(5):876-883:10.1093/rheumatology/keu389
13. Adams J, Barratt P, Rombach I, Arden N, Barbosa Bouças S, Bradley S, et al. The clinical and cost effectiveness of splints for thumb base osteoarthritis: a randomized controlled clinical trial. *Rheumatology (Oxford, England).* 2021;60(6):2862-2877:10.1093/rheumatology/keaa726
14. Kjeken I, Eide RE, Klokkeide A, Matre KH, Olsen M, Mowinckel P, et al. Does occupational therapy reduce the need for surgery in carpometacarpal osteoarthritis? Protocol for a randomized controlled trial. *BMC musculoskeletal disorders.* 2016;17(1):473:10.1186/s12891-016-1321-3
15. Herdman M, Gudex C, Lloyd A, Janssen M, Kind P, Parkin D, et al. Development and preliminary testing of the new five-level version of EQ-5D (EQ-5D-5L). *Qual Life Res.* 2011;20(10):1727-1736:10.1007/s11136-011-9903-x

For peer review only - http://bmjopen.bmj.com/site/about/guidelines.xhtml

16. Statens legemiddelverk. Guidelines for the submission of documentation for single technology assessment (STA) of pharmaceuticals. https://legemiddelverket.no/Documents/English/Public%20funding%20and%20pricing/Documentation%20for%20STA/Guidelines%2020.05.2020.pdf. Published 2018. Accessed 8.6.21, 2021.
17. Helsedirektoratet. [DRG-systemet]. https://www.helsedirektoratet.no/tema/finansiering/innsatsstyrt-finansiering-og-drg-systemet/drg-systemet#omdrgsystemet%E2%80%8B. Accessed 8.6.21, 2021.
18. Statistics Norway. [*Salary*]. https://www.ssb.no/arbeid-og-lonn/statistikker/lonnansatt. Accessed 20.08.2020.
19. Arbeidstilsynet. [*Minimum wage*]. https://www.arbeidstilsynet.no/arbeidsforhold/lonn/minstelonn/. Accessed 20.08.2020.
20. Drummond MF, Sculpher MJ, Claxton K, Stoddart GL, Torrance GW. *Methods for the Economic Evaluation of Health Care Programmes.* Fourth edition ed. Oxford: Oxford University Press; 2015.
21. Norwegian Ministry of Health and Care Services. Principles for priority setting in health care. Summary of a white paper on priority setting in the Norwegian health care sector. In. https://www.regjeringen.no/contentassets/439a420e01914a18b21f351143ccc6af/en-gb/pdfs/stm201520160034000engpdfs.pdf2017.
22. Dziedzic K, Nicholls E, Hill S, Hammond A, Handy J, Thomas E, et al. Self-management approaches for osteoarthritis in the hand: a 2x2 factorial randomised trial. *Annals of the rheumatic diseases.* 2015;74(1):108-118:10.1136/annrheumdis-2013-203938
23. Ekman B, Nero H, Lohmander LS, Dahlberg LE. Costing analysis of a digital first-line treatment platform for patients with knee and hip osteoarthritis in Sweden. *PLoS One.* 2020;15(8):e0236342:10.1371/journal.pone.0236342
24. Tveter A, Blanck T, Nyheim S, Maarnes M, Christensen B, Pedersen S, et al. OP0122-HPR DEVELOPMENT OF A SMARTPHONE APPLICATION FOR TREATMENT OF HAND OSTEOARTHRITIS – HAPPY HANDS *Ann Rheum Dis.* 2022;81:79:DOI: 10.1136/annrheumdis-2022-eular.4343
25. Sanders GD, Neumann PJ, Basu A, Brock DW, Feeny D, Krahn M, et al. Recommendations for Conduct, Methodological Practices, and Reporting of Cost-effectiveness Analyses: Second Panel on Cost-Effectiveness in Health and Medicine. *JAMA.* 2016;316(10):1093-1103:10.1001/jama.2016.12195

For peer review only

For peer review only - http://bmjopen.bmj.com/site/about/guidelines.xhtml

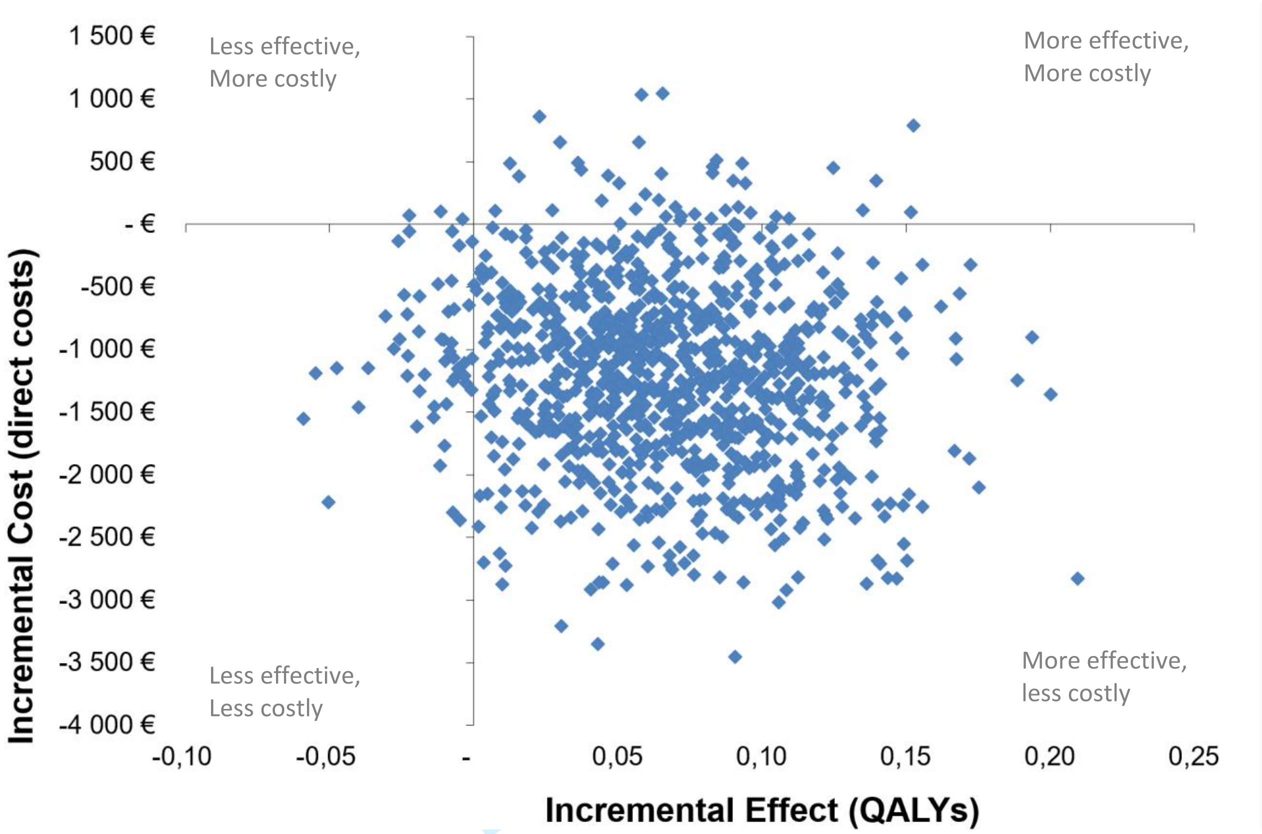
Less effective,
More costly
More effective,
More costly
Less effective,
Less costly
More effective,
less costly
1 500 €
1 000 €
500 €
- €
-500 €
-1 000 €
-1 500 €
-2 000 €
-2 500 €
-3 000 €
-3 500 €
-4 000 €
-0,10
-0,05
-
0,05
0,10
0,15
0,20
0,25
Incremental Cost (direct costs)
Incremental Effect (QALYs)

Figure 1

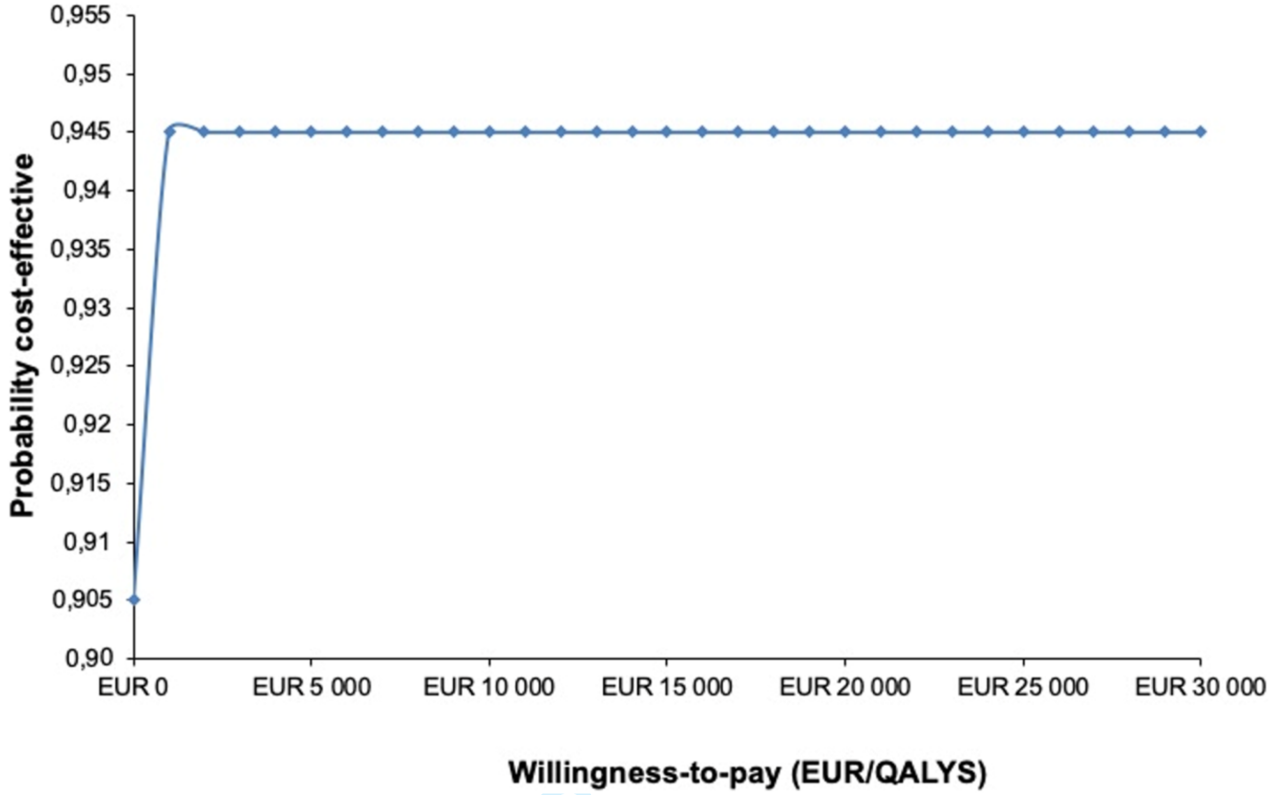
0,955
0,95
0,945
0,94
0,935
0,93
0,925
0,92
0,915
0,91
0,905
0,90
Probability cost-effective
EUR 0
EUR 5 000
EUR 10 000
EUR 15 000
EUR 20 000
EUR 25 000
EUR 30 000
Willingness-to-pay (EUR/QALYS)

Figure 2

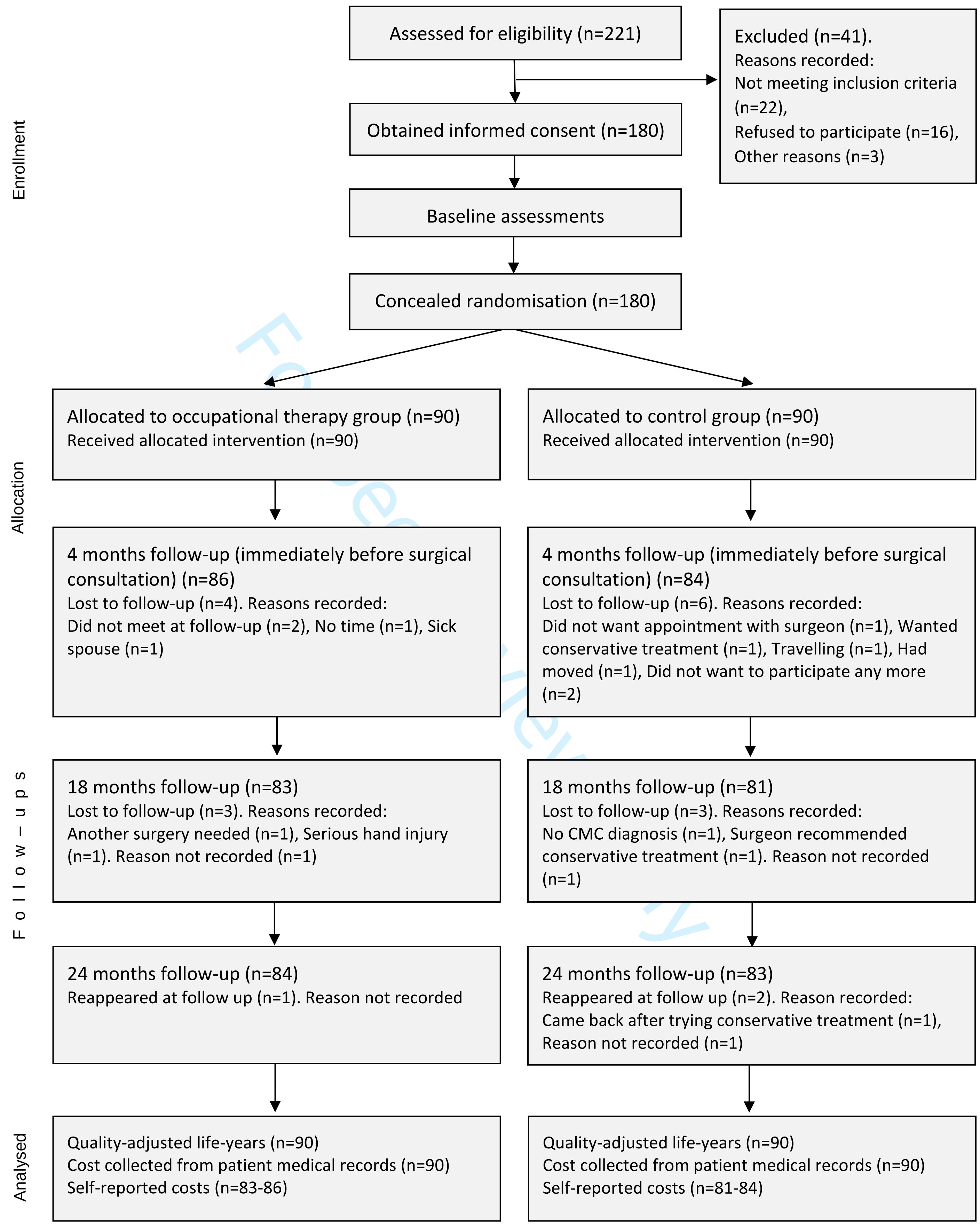

Supplementary figure A

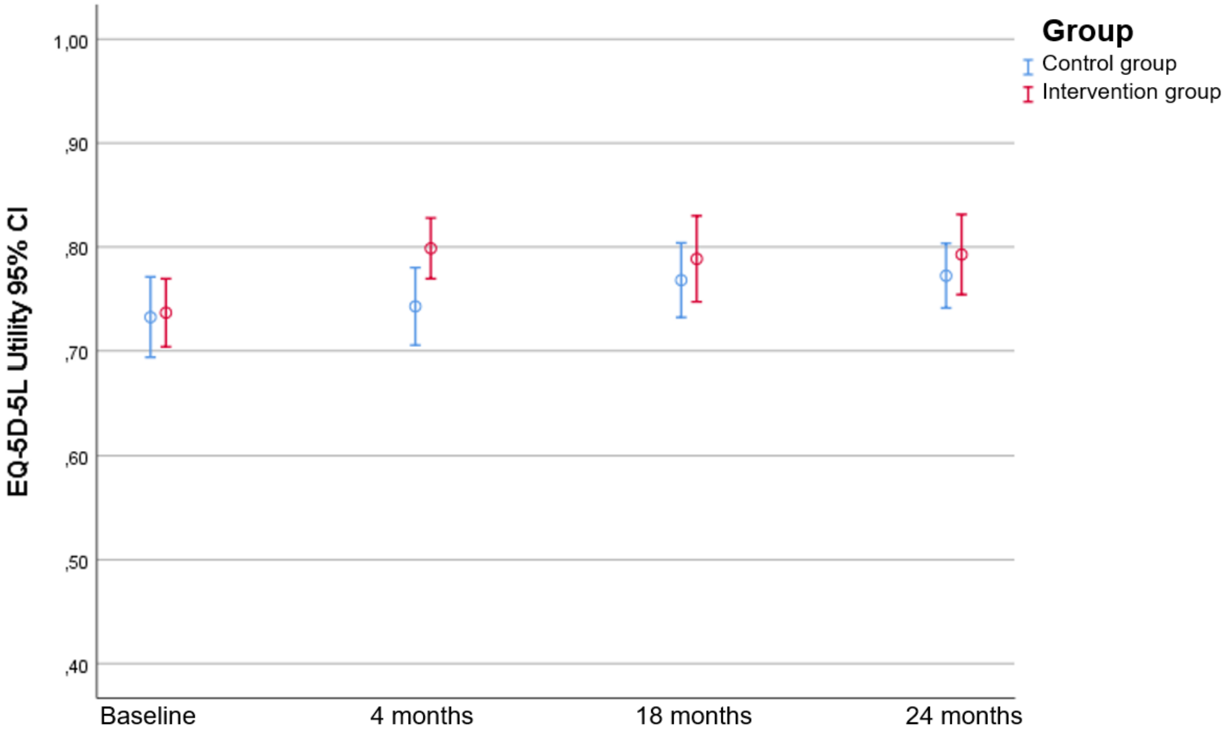

Supplementary figure B

For peer review only

CHEERS 2022 Checklist

**Title**			
Title	1	Identify the study as an economic evaluation and specify the interventions being compared.	page 1
**Abstract**			
Abstract	2	Provide a structured summary that highlights context, key methods, results, and alternative analyses.	pag 2
**Introduction**			
Background and objectives	3	Give the context for the study, the study question, and its practical relevance for decision making in policy or practice.	page 3-4
Methods			
**Health economic analysis plan**	**4**	**Indicate whether a health economic analysis plan was developed and where available.**	**page 3**
Study population	5	Describe characteristics of the study population (such as age range, demographics, socioeconomic, or clinical characteristics).	table 1
Setting and location	6	Provide relevant contextual information that may influence findings.	page 3-4
Comparators	7	Describe the interventions or strategies being compared and why chosen.	page 5
Perspective	8	State the perspective(s) adopted by the study and why chosen.	page 4
Time horizon	9	State the time horizon for the study and why appropriate.	page 4

For peer review only

(continued)

Discount rate	10	Report the discount rate(s) and reason chosen.	page 6-7
Selection of outcomes	11	Describe what outcomes were used as the measure(s) of benefit(s) and harm(s).	page 6-7
Measurement of outcomes	12	Describe how outcomes used to capture benefit(s) and harm(s) were measured.	page 6-7
Valuation of outcomes	13	Describe the population and methods used to measure and value outcomes.	page 4, page 6-7
Measurement and valuation of resources and costs	14	Describe how costs were valued.	page 7
Currency, price date, and conversion	15	Report the dates of the estimated resource quantities and unit costs, plus the currency and year of conversion.	table 2
Rationale and description of model	16	If modelling is used, describe in detail and why used. Report if the model is publicly available and where it can be accessed.	ua
Analytics and assumptions	17	Describe any methods for analysing or statistically transforming data, any extrapolation methods, and approaches for validating any model used.	page 7-8
Characterising heterogeneity	18	Describe any methods used for estimating how the results of the study vary for subgroups.	ua
Characterising distributional effects	19	Describe how impacts are distributed across different individuals or adjustments made to reflect priority populations.	ua

For peer review only - http://bmjopen.bmj.com/site/about/guidelines.xhtml

(continued)

Characterising uncertainty	20	Describe methods to characterise any sources of uncertainty in the analysis.	page 7
Approach to engagement with patients and others affected by the study	21	Describe any approaches to engage patients or service recipients, the general public, communities, or stakeholders (such as clinicians or payers) in the design of the study.	page 4
**Results**			
Study parameters	22	Report all analytic inputs (such as values, ranges, references) including uncertainty or distributional assumptions.	table 3, figure 1
Summary of main results	23	Report the mean values for the main categories of costs and outcomes of interest and summarise them in the most appropriate overall measure.	page 8-9, table 3
Effect of uncertainty	24	Describe how uncertainty about analytic judgments, inputs, or projections affect findings. Report the effect of choice of discount rate and time horizon, if applicable.	page 9-11
Effect of engagement with patients and others affected by the study	25	Report on any difference patient/service recipient, general public, community, or stakeholder involvement made to the approach or findings of the study	page 4
Discussion			

For peer review only - http://bmjopen.bmj.com/site/about/guidelines.xhtml

(continued)

Study findings, limitations, generalisability, and current knowledge	26	Report key findings, limitations, ethical or equity considerations not captured, and how these could affect patients, policy, or practice.	page 9-11
Other relevant information			
Source of funding	27	Describe how the study was funded and any role of the funder in the identification, design, conduct, and reporting of the analysis	page 13
Conflicts of interest	28	Report authors conflicts of interest according to journal or International Committee of Medical Journal Editors requirements.	page 13

From: Husereau D, Drummond M, Augustovski F, et al. Consolidated Health Economic Evaluation Reporting Standards 2022 (CHEERS 2022) Explanation and Elaboration: A Report of the ISPOR CHEERS II Good Practices Task Force. Value Health 2022;25. doi:10.1016/j.jval.2021.10.008

For peer review only
